# Phytochemical Profile, Vasodilatory and Biphasic Effects on Intestinal Motility, and Toxicological Evaluation of the Methanol and Dichloromethane Extracts from the Aerial Parts of *Ipomoea purpurea* Used in Traditional Mexican Medicine

**DOI:** 10.3390/ph18081134

**Published:** 2025-07-30

**Authors:** Valeria Sánchez-Hernández, Francisco J. Luna-Vázquez, María Antonieta Carbajo-Mata, César Ibarra-Alvarado, Alejandra Rojas-Molina, Beatriz Maruri-Aguilar, Pedro A. Vázquez-Landaverde, Isela Rojas-Molina

**Affiliations:** 1 Programa de Maestría en Ciencias Químico Biológicas, Facultad de Química, Universidad Autónoma de Querétaro, Cerro de las Campanas S/N, Santiago de Queretaro 76017, Querétaro, Mexico; vsanchez25@alumnos.uaq.mx; 2 Laboratorio de Investigación Química y Farmacológica de Productos Naturales, Facultad de Química, Universidad Autónoma de Querétaro, Cerro de las Campanas S/N, Santiago de Queretaro 76017, Querétaro, Mexico; francisco.luna@uaq.mx (F.J.L.-V.); cibarra@uaq.mx (C.I.-A.); rojasa@uaq.mx (A.R.-M.); 3 Laboratorio Universitario Bioterio, Instituto de Neurobiología, Universidad Nacional Autónoma de México, Campus Juriquilla, Santiago de Queretaro 76230, Querétaro, Mexico; mariacarbajomata@comunidad.unam.mx; 4 Jardín Botánico Regional de Cadereyta “Ing. Manuel González de Cosío” del Consejo de Ciencia y Tecnología del Estado de Querétaro, Cadereyta 76500, Querétaro, Mexico; bmaruri@concyteq.edu.mx; 5 Centro de Investigación en Ciencia Aplicada y Tecnología Avanzada del Instituto Politécnico Nacional, Unidad Querétaro, Cerro Blanco No. 141, Col. Colinas del Cimatario, Santiago de Queretaro 76090, Querétaro, Mexico; pavazquez@ipn.mx

**Keywords:** *Ipomoea purpurea*, phytochemical profile, smooth muscle, biphasic effect, vasodilatory effect, traditional Mexican medicine, plant extracts, phytotherapy, toxicological study

## Abstract

**Background:** Cardiovascular diseases, particularly hypertension, and gastrointestinal disorders represent major public health concerns in Mexico. Although a range of pharmacological treatments exists, their use is associated with adverse effects, highlighting the need for safer therapeutic alternatives. Species of the *Ipomoea* genus are widely employed in Mexican traditional medicine (MTM) for their purgative, anti-inflammatory, analgesic, and sedative properties. Particularly, *Ipomoea purpurea* is traditionally used as a diuretic and purgative; its leaves and stems are applied topically for their anti-inflammatory and soothing effects. This study aimed to determine their phytochemical composition and to evaluate the associated vasodilatory activity, modulatory effects on intestinal smooth-muscle motility, and toxicological effects of the methanolic (ME-Ip) and dichloromethane (DE-Ip) extracts obtained from the aerial parts of *I. purpurea*. **Methods:** The phytochemical composition of the ME-Ip and DE-Ip extracts of *I. purpurea* was assessed using UPLC-QTOF-MS and GC-MS, respectively. For both extracts, the vasodilatory activity and effects on intestinal smooth muscle were investigated using ex vivo models incorporating isolated rat aorta and ileum, respectively, whereas acute toxicity was evaluated in vivo. **Results:** Phytochemical analysis revealed, for the first time, the presence of two glycosylated flavonoids within the *Ipomoea* genus; likewise, constituents with potential anti-inflammatory activity were detected. The identified compounds in *I. purpurea* extracts may contribute to the vasodilatory, biphasic, and purgative effects observed in this species. The EC_50_ values for the vasodilatory effects of the methanolic (ME-Ip) and dichloromethane (DE-Ip) extracts were 0.80 and 0.72 mg/mL, respectively. In the initial phase of the experiments on isolated ileal tissues, both extracts induced a spasmodic (contractile) effect on basal motility, with ME-Ip exhibiting higher potency (EC_50_ = 27.11 μg/mL) compared to DE-Ip (EC_50_ = 1765 μg/mL). In contrast, during the final phase of the experiments, both extracts demonstrated a spasmolytic effect, with EC_50_ values of 0.43 mg/mL for ME-Ip and 0.34 mg/mL for DE-Ip. In addition, both extracts exhibited low levels of acute toxicity. **Conclusions:** The phytochemical profile and the vasodilatory and biphasic effects of the *I. purpurea* extracts explain, in part, the use of *I. purpurea* in MTM. The absence of acute toxic effects constitutes a preliminary step in the toxicological safety assessment of *I. purpurea* extracts and demonstrates their potential for the development of phytopharmaceutic agents as adjuvants for the treatment of cardiovascular and gastrointestinal disorders.

## 1. Introduction

In Mexico, the numerous health challenges demand a coordinated response from the health sector [1]. These challenges largely stem from socioeconomic inequalities, which manifest in disparate health outcomes among different social strata. For instance, marginalized and indigenous populations experience disproportionately high rates of morbidity associated with unsafe water supplies, infectious gastrointestinal diseases, respiratory illnesses, and other conditions [2].

In contrast, according to the National Institute of Public Health, the Mexican population has undergone a significant nutritional transition over recent decades, characterized by a shift in dietary patterns and a decline in physical activity. These changes have contributed to a rise in chronic non-communicable diseases (NCDs), including obesity, hypertension, and diabetes—conditions collectively responsible for approximately 41 million deaths annually worldwide [3].

Arterial hypertension (AH) is a leading risk factor for the development of cardiovascular diseases. In Mexico, an estimated 30 million individuals are affected by AH, which constitutes the primary cause of mortality in the country, accounting for approximately 50,000 deaths annually [4,5]. Management strategies for AH typically involve the administration of vasodilator drugs, such as calcium-channel blockers, nitrovasodilators, potassium channel activators, and α1-adrenergic blockers [6]. However, these medications are often associated with adverse effects—including tachyarrhythmias and fluid retention—as well as the development of pharmacological tolerance. As a result, they are frequently prescribed in combination with β-blockers and diuretics to mitigate these side effects [7]. However, polypharmacy contributes to poor patient adherence to treatment regimens [8].

Gastrointestinal diseases (GDs) also pose a major public health concern in Mexico. They are the leading cause of death among children, the elderly, and immunocompromised individuals, and represent the most common reason for seeking medical care [9,10]. GDs are multifactorial in origin, and their pharmacological management primarily targets symptomatic relief of dyspepsia, dysphagia, nausea, vomiting, heartburn, aerophagia, constipation, intestinal spasms, and diarrhea [11]. Intestinal spasms—characterized by painful and involuntary contractions of the intestinal musculature—are commonly treated with antispasmodic agents, including anticholinergics, calcium-channel blockers, and direct smooth-muscle relaxants [12]. Nevertheless, these drugs can cause adverse effects such as urinary retention, constipation, drowsiness, and tachycardia, which limits their use in vulnerable populations such as children, the elderly, and pregnant women [13].

Moreover, both hypertension and gastrointestinal disorders have been implicated in the initiation of inflammatory processes—an immune response that can lead to tissue damage in affected organs [14,15]. While pharmacological agents remain the first-line treatments for AH and GD symptoms, their use is often associated with significant limitations. Consequently, medicinal plants are increasingly considered viable therapeutic alternatives [16], especially in developing countries, in which approximately 90% of the population relies on traditional medicine [17].

Mexico is recognized for its rich floristic diversity; notably, though, only around 5% of the endemic plant species used in traditional medicine have undergone chemical and pharmacological validation [18,19]. The Convolvulaceae family, which includes about 50 genera and 1650 species, is well represented in Mexico, with approximately 313 species documented. Particularly, the genera *Ipomoea*, *Convolvulus*, *Exogonium*, and *Operculina* are of particular importance for their medicinal properties [20,21].

Among these, the genus *Ipomoea* has received attention for its potential pharmacological activities. Aqueous and organic extracts from the aerial parts and roots of various *Ipomoea* species, including *I. hederacea*, *I. pes-caprae*, *I. tyrianthina*, *I. acanthocarpa*, *I. stans*, and *I. imperati*, have been evaluated in vitro and in vivo for vasodilatory, antihypertensive, and/or antispasmodic effects. Several studies have also suggested possible mechanisms of action underlying these pharmacological properties [22,23,24,25,26].

In various traditional medical systems across the Americas, Asia, and Africa, different parts of *Ipomoea* species such as *I. imperati*, *I. pes-caprae*, *I. hederacea*, *I. asarifolia*, *I. carnea*, *I. involucrata*, *I. stolonifera*, *I. obscura*, and *I. batatas* are used to treat inflammatory conditions, including rheumatism, rheumatoid arthritis, dermatitis, cancer, and type 2 diabetes mellitus [27,28]. In the case of *Ipomoea purpurea*, its seeds have been employed in ancestral rituals for their hallucinogenic properties, while infusions of its aerial parts are traditionally used as diuretics and purgatives. Poultices made from the leaves and stems are also applied for their soothing and anti-inflammatory effects on the skin [29,30]. Several *Ipomoea* species exhibit pharmacological activities attributed to phenolic acids, flavonoids, and resin glycosides. These compounds act via mechanisms such as calcium-channel blockade, nitric oxide (NO) modulation, and NF-κB inhibition, contributing to spasmolytic, vasorelaxant, antioxidant, and anti-inflammatory effects [23,31,32,33].

*Ipomoea purpurea* is a resilient species, capable of withstanding diverse climatic and soil conditions. However, it is considered an invasive agricultural weed, and its biomass is often discarded without being utilized [34,35]. *Ipomoea purpurea*, like other species of the *Ipomoea* genus, exhibits resistance to both chemical herbicides and biological control agents [23]. Consequently, the utilization of this species provides an opportunity to render its regular harvesting economically viable, thereby contributing to its population management. In this context, various efforts have been made to exploit *Ipomoea* species as sources of volatile fatty acids to generate clean energy [36], biosorbents [37], chemical compounds [38], and pharmaceutical agents [39], among others. Despite the traditional uses of the plant, pharmacological research on *I. purpurea* remains limited, with existing studies primarily focused on its phytochemical profile and antioxidant capacity, and the anticancer properties of its leaf extracts [40,41].

Therefore, the present study aims to characterize the phytochemical composition and assess the vasodilatory and gastrointestinal smooth-muscle effects, as well as the acute toxicity, of methanolic and dichloromethane extracts derived from the aerial parts of *Ipomoea purpurea*, using ex vivo and in vivo models. We hypothesize that these extracts exhibit significant pharmacological effects, namely vasodilatory activity and alterations in intestinal motility, without inducing acute toxicity. These effects are presumed to be mediated by the presence of bioactive secondary metabolites such as bicyclic sesquiterpenes, norisoprenoids, triterpenes, diterpenes, steroidal glycosides, phenylpropanoids, flavonoid glycosides, and polyphenols.

## 2. Results and Discussion

### 2.1. Phytochemical Tests of Methanolic Extract (ME-Ip) and Dichloromethane Extract (DE-Ip) of Aerial Parts of Ipomoea purpurea for the Presence of Secondary Metabolites

Table 1 shows the qualitative analysis of secondary metabolites detected in the methanolic (ME-Ip) and dichloromethane (DE-Ip) extracts of the aerial parts of *Ipomoea purpurea*. These results are consistent with those obtained by Beheshti et al. [41], who conducted the most recent phytochemical study of the same species, using specimens collected in Iran. These authors used ethyl alcohol as the extraction solvent and identified flavonoids, steroids, and tannins. Likewise, these authors studied the chloroformic extract of *I. purpurea* and detected the presence of flavonoids and steroids. Unlike the previously cited study, the analysis of the secondary metabolites of ME-Ip and DE-Ip presented in Table 1 was complemented by additional analysis of UPLC-MS and CG-MSP, the results of which are shown later, in Section 2.3 and 2.4, respectively.

### 2.2. Total Phenolic Compounds and Antioxidant Capacity of Methanolic Extract of Aerial Parts of Ipomoea purpurea (ME-Ip)

Given the importance of phenolic compounds and flavonoids in the prevention of cardiovascular diseases [42], the content levels of these bioactive compounds were quantified in the methanolic extract of *Ipomoea purpurea* (ME-Ip). The results are shown in Table 2, and they indicate higher concentrations than those previously reported by Beheshti et al. [41], who documented values of 28.78 ± 0.1 mg GAE (gallic acid equivalents)/g dry extract for total phenolics and 19.9 ± 0.3 mg CAE (catechin equivalents)/g dry extract for flavonoids. These discrepancies may be attributed to variations in environmental and edaphic conditions affecting the biosynthesis of secondary metabolites [43].

Regarding antioxidant capacity, *I. purpurea* demonstrated lower activity compared to aqueous extracts of *Ipomoea batatas* (25.16 ± 0.13 μmol Trolox/g dry extract; 68.63 ± 0.34% inhibition) and *Camellia sinensis* leaves (25.63 ± 0.58 μmol Trolox/g dry extract; 69.88 ± 1.55% inhibition), both recognized for their potent antioxidant profiles [44]. Nevertheless, *I. purpurea* exhibited a higher phenolic content than other species within the genus, such as *I. mauritiana*, which has been reported to contain 8.2 ± 0.16 mg/g dry extract [45]. Interestingly, the methanolic extract of *I. purpurea* leaves has demonstrated antiproliferative and pro-apoptotic effects in lung and breast cancer cell lines, effects that have been associated with its flavonoid and phenolic compound contents [40,41].

### 2.3. UPLC-QTOF-MS Analysis of Methanolic Extract of Aerial Parts of Ipomoea purpurea (ME-Ip)

The chemical profile of the methanolic extract of *Ipomoea purpurea* (ME-Ip) via UPLC-QTOF-MS is shown in Table 3. The analysis revealed a metabolite composition predominantly comprising phenolic acids and flavonoids, many of which have been previously documented in other species of the *Ipomoea* genus. Compounds such as caffeic acid, chlorogenic acid, ferulic acid, 3,4-di-O-caffeoylquinic acid, N-cis-feruloylthiamine, (−)-arctigenin, and betulinic acid have been identified in species including *I. batatas*, *I. muricata*, *I. aquatica*, *I. cairica*, and *I. pes-caprae*, and are known to exhibit a broad spectrum of pharmacological properties, including antioxidant, anti-inflammatory, hepatoprotective, and anticancer activities [46,47,48,49]. Categorically speaking, the phenolic acids detected in the extract are recognized for their capacity to scavenge free radicals and modulate the pro-inflammatory signaling pathways such as NF-κB and COX-2. Likewise, lignans like arctigenin, and triterpenes such as betulinic acid, have demonstrated antiviral and pro-apoptotic effects in various cellular models [47,48,49,50].

Furthermore, the UPLC-QTOF-MS analysis (Table 3) identified metabolites that, to the best of our knowledge, have not been previously reported in the *Ipomoea* genus. These include apigenin-7-glucuronide and diosmetin 7-O-β-D-glucopyranoside (flavonoids well-known for their anti-inflammatory, anxiolytic, and neuroprotective properties). These compounds in *I. purpurea* suggest a potential species-specific biosynthetic pathway or an adaptive response to distinct environmental factors, such as climatic conditions or soil composition [46].

Alkaloids were not detected in the UPLC-QTOF-MS analysis; this result is consistent with both the absence of these compounds in the phytochemical screening of ME-Ip (Table 1) and with indications in the prior literature that ergoline alkaloids, such as lysergic acid and its derivatives, which are characteristic of the *Ipomoea* genus, are predominantly confined to the seeds of *I. purpurea*, and not found in its aerial parts [29,30]. This finding reinforces the hypothesis that the observed biological activities of the extract are likely attributable to its phenolic and flavonoid constituents rather than to ergoline alkaloids. Fragmentation patterns used to confirm the identity of the compounds detected by UPLC-QTOF-MS analysis are provided in the Supplementary Material (Table S1).

### 2.4. GC-MS Analysis of Dichloromethane Extract of Aerial Parts of Ipomoea purpurea (DE-Ip)

Table 4 shows the phytochemical profile of the dichloromethane extract of *Ipomoea purpurea* (DE-Ip), indicating the presence of 49 volatile metabolites.

The most recent GC-MS-based report on the chemical composition of *I. purpurea* was published by Beheshti et al. [41], focusing on a species native to Iran. In that study, 14 metabolites were identified, predominantly consisting of alkanes and alcohols. Additionally, Ono et al. [51] reported the occurrence of n-octanoic acid, operculinic acid E, methyl n-octanoate, and methyl n-decanoate, which are derived from the hydrolysis of the ether-soluble resinous compound (Jalapino) characteristic of the Convolvulaceae family.

As shown in Table 4, the major chemical compounds identified include carboxylic acids (11), terpenoids (11), esters (8), and alkanes (8). Among these, n-octanoic acid was detected with a relative abundance of 1.42%. This medium-chain fatty acid is recognized for its antimicrobial, antifungal, and anti-inflammatory activities [52]. In addition, this compound has been previously reported in other *Ipomoea* species and in various medicinal plants, where its presence is considered to contribute significantly to the biological activity of plant-derived extracts [53,54].

The most abundant compounds identified in the DE-Ip were tetracosane (12.75%) and heneicosane (8.68%); both compounds are long-chain alkanes associated with anti-inflammatory, analgesic, and antipyretic properties [53]. The third-most-abundant metabolite was n-hexadecanoic acid (7.80%), a saturated fatty acid widely known for its anti-inflammatory, antioxidant, and antimicrobial effects [55,56].

To date, studies focused on the chemical composition of extracts from the aerial parts of *I. purpurea* are scarce. Variations in metabolite profiles are largely influenced by factors such as the extraction methodology, solvent polarity, and the plant’s geographical origin, which encompasses specific environmental conditions [41,43]. For the most part, several metabolites identified in the DE-Ip extract, including caryophyllene, n-hexadecanoic acid, and phytol, have also been previously reported in other *Ipomoea* species [25,57]. These findings are consistent with the results of the qualitative phytochemical screening of DE-Ip (Table 1), which detected terpenoids. Their presence aligns with their chemical nature, as terpenoids exhibit greater solubility in non-polar solvents such as dichloromethane, compared to polar solvents like methanol.

### 2.5. Vasodilator Effects of ME-Ip and DE-Ip on Isolated Rat Aorta Assay

**Figure 1:**
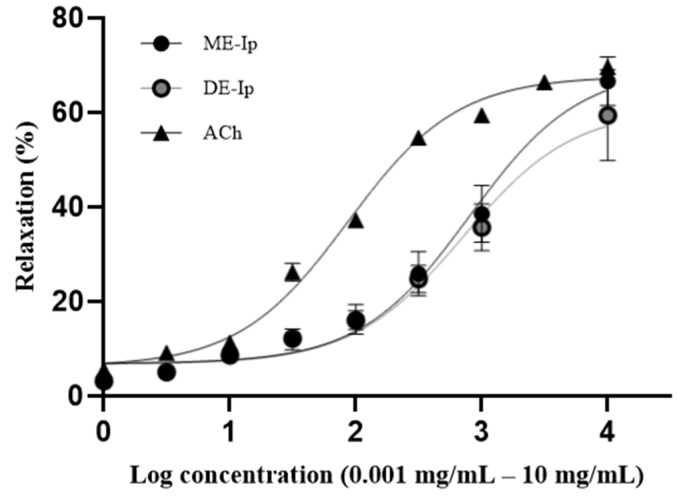
Concentration–response curves showing the vasodilatory effects of the methanolic (ME-Ip) and dichloromethane (DE-Ip) extracts from the aerial parts of *Ipomoea purpurea*, as well as those of acetylcholine (ACh), on isolated rat aortic rings. Data are presented as mean ± SEM (*n* = 6 per group). Statistically significant differences in EC_50_ values were observed for both ME-Ip and DE-Ip when compared to ACh (*p* < 0.05). No significant difference was found between ME-Ip and DE-Ip (*p* > 0.05). Significant differences were observed between E_max_ of ME-Ip and DE-Ip, and between E_max_ of DE-Ip and ACh (*p* < 0.05), no significant difference was found between E_max_ of ME-Ip and ACh (*p* > 0.05).

The dichloromethane (DE-Ip) and methanolic (ME-Ip) extracts obtained from the aerial parts of *Ipomoea purpurea* induced a concentration-dependent relaxation of isolated aortic rings. Figure 1 illustrates the concentration–response curves ass determined for both extracts, as well as for acetylcholine (ACh), which served as a vasodilator compound. The ME-Ip extract exhibited an EC_50_ of 802.3 ± 62.60 µg/mL, whereas the DE-Ip extract displayed an EC_50_ of 718.9 ± 53.90 µg/mL. Both values indicate an approximately 10-fold lower potency in comparison to ACh (EC_50_ = 87.01 ± 1.85 µg/mL). The EC_50_ is a standard pharmacological parameter used to quantify the potency of bioactive compounds; lower EC_50_ values correspond to higher potency [58]. Consequently, the observed EC_50_ values suggest that both extracts are substantially less potent than ACh, a compound frequently used as a reference in smooth-muscle studies due to its well-characterized vasorelaxant effect, mediated primarily through M_3_ muscarinic receptors [59]. Statistically significant differences in EC_50_ values were observed for both ME-Ip and DE-Ip compared to ACh (*p* = 0.0111 and *p* = 0.0073, respectively; *p* < 0.05). However, no significant difference was found between the EC_50_ values of the two extracts (*p* = 0.3342; *p* > 0.05). Regarding efficacy, the ME-Ip extract achieved an E_max_ = 69.40 ± 2.61%, while the DE-Ip extract reached an E_max_ = 60.8 ± 2.14%. Statistically significant differences in E_max_ values were observed between ME-Ip and DE-Ip (*p* = 0.005; *p* < 0.05), as well as between DE-Ip and ACh (E_max_ = 68.81 ± 1.3%) (*p* = 0.011; *p* < 0.05). In contrast, no significant difference was found in E_max_ between ME-Ip and ACh (*p* = 0.788; *p* > 0.05).

Several metabolites identified by UPLC-QTOF-MS, such as caffeic acid, astragalin, betulinic acid, chlorogenic acid, ferulic acid, and arctigenin, as well as compounds detected via GC-MS, including caryophyllene, phytol, and squalene, have previously demonstrated vasodilatory activity in experimental models involving isolated aortic tissue [60,61,62,63,64,65,66,67,68]. Nonetheless, although these compounds were detected in the tested extracts, their concentrations may be insufficient to elicit a vasodilatory response comparable to that of acetylcholine. Therefore, further studies are necessary to elucidate additional mechanisms that contribute to the observed vasodilatory activity. These mechanisms could include angiotensin-converting enzyme (ACE) inhibition or diuretic effects [60,61,62,63,64,65,66,67,68], especially considering that *I. purpurea* has been traditionally used as a diuretic agent, as documented in various herbal compilations [29,30].

The vasodilatory effect of carbachol, a well-established cholinergic agonist that primarily acts on M_3_ muscarinic receptors, has been reported to induce arterial smooth-muscle vasodilation with an EC_50_ = 0.031 ± 0.0029 µg/mL and an E_max_ = 86.8 ± 3.6%, under ex vivo conditions comparable to those used in the present study [69]. Comparing these values with the EC_50_ = 802.3 ± 62.60 µg/mL for ME-Ip and EC_50_ = 718.9 ± 53.90 µg/mL for DE-Ip, it is evident that both *I. purpurea* extracts exhibit a lower potency than that of carbachol.

Definitely, pharmacokinetic studies are critical components in the evaluation of the pharmacological effects of plant extracts and their active constituents, as limited absorption can significantly hinder both their therapeutic application and the development of phytopharmaceuticals. In this context, caffeic acid—identified as one of the most abundant phenolic compounds in ME-Ip—has been reported to exhibit poor oral bioavailability (14.7%) in Sprague Dawley rats [70]. Similarly, in a study by Godugu et al. [71], oral administration of betulinic acid, also detected in ME-Ip (Table 3), to Sprague Dawley rats resulted in a maximum plasma concentration (C_max_) of 1.16 µg/mL after 2.36 h of exposure. This finding highlights the extremely low bioavailability of this pentacyclic triterpene, which has been attributed to its poor aqueous solubility (~0.02 µg/mL) [72]. Consequently, numerous studies have focused on the development of advanced drug delivery systems aimed at improving the bioavailability and stability of such bioactive compounds, while minimizing potential side effects [73].

### 2.6. Spasmodic and Spasmolytic Effects of ME-Ip and DE-Ip on Isolated Rat Ileum Assay

**Figure 2:**
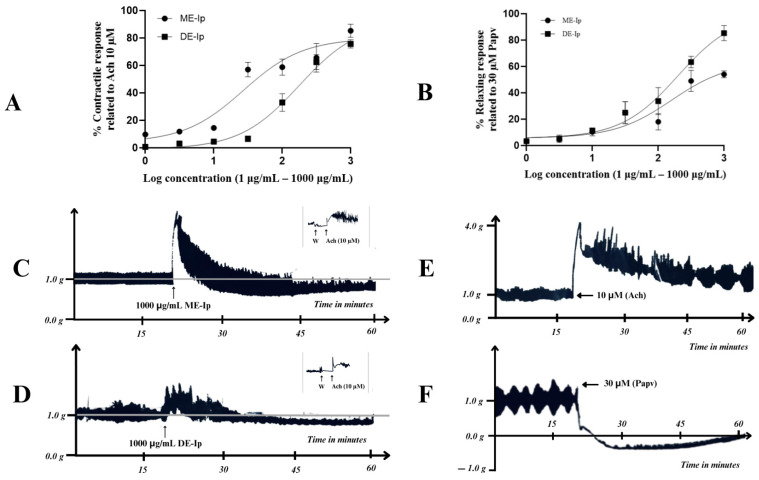
(**A**) Concentration–response curves of the contractile effects of the methanolic (ME-Ip) and dichloromethane (DE-Ip) extracts from the aerial parts of *I. purpurea* on isolated rat ileum; contraction is expressed as the percentage of the contractile response induced by acetylcholine (Ach, 10 µM). Data are presented as mean ± SEM (*n* = 6), No statistically significant differences were observed (*p* value > 0.05, *p* value = 0.333). (**B**) Concentration–response curves showing the relaxant effects of ME-Ip and DE-Ip on isolated rat ileum preincubated for 30 min; relaxation is expressed as a percentage of the maximal effect induced by papaverine (Papv, 30 μM). Data are presented as mean ± SEM (*n* = 6); no statistically significant differences were observed (*p* value > 0.05, *p* value = 0.5365). (**C**) Representative tracing of smooth-muscle activity in isolated rat ileum, recorded over a 60 min period following administration of ME-Ip (1000 μg/mL). (**D**) Representative tracing of smooth-muscle activity in isolated rat ileum recorded over a 60 min period, following the administration of DE-Ip (1000 μg/mL). The inserts in Figure 2C,D confirm the functional viability of the tissue after 60 min of experimentation. (**E**) Representative response to acetylcholine (ACh, 10 μM) in isolated rat ileum. (**F**) Representative response to papaverine (Papv, 30 μM) in isolated rat ileum.

The effects of the methanolic (ME-Ip) and dichloromethane (DE-Ip) extracts of *Ipomoea purpurea* aerial parts on the spontaneous motility of isolated rat ileum are presented in Figure 2. In the concentration–response curves (Figure 2A), both extracts exhibited a spasmodic (contractile) effect on the basal motility of ileal tissue, with ME-Ip demonstrating greater potency (EC_50_ = 27.11 ± 1.44 μg/mL) and efficacy (E_max_ = 85.33 ± 4.81%) compared to DE-Ip (EC_50_ = 176.0 ± 2.25 μg/mL; E_max_ = 75.71 ± 2.98%). Statistically significant differences were observed between the EC_50_ values (*p* < 0.05; *p* = 0.0001) and E_max_ values (*p* < 0.05; *p* = 0.0126). However, approximately 35 min after administration, a progressive decrease in both the amplitude and frequency of peristaltic contractions was observed, along with a sustained reduction in the initial contractile force (~1 g) for both extracts (Figure 2B). This inhibitory effect persisted up to 60 min of recording. Under these conditions, ME-Ip showed an EC_50_ = 437.1 ± 11.44 μg/mL and an E_max_ = 52.5 ± 5.04%, whereas DE-Ip exhibited an EC_50_ = 339.5 ± 10.10 μg/mL and an E_max_ = 85.32 ± 7.73%. Although no statistically significant differences were found between the EC_50_ values (*p* > 0.05; *p* = 0.5365), the difference in E_max_ values was statistically significant (*p* < 0.05; *p* = 0.0081).

Based on the above findings, it can be inferred that both extracts exhibit a biphasic effect (Figure 2C,D), characterized by an initial excitatory phase resembling that induced by acetylcholine (Figure 2E), followed by a sustained inhibitory phase consistent with a spasmolytic effect similar to that of papaverine (Figure 2F). At the conclusion of both experimental protocols, prior to removing the tissue from the organ bath, a wash with Krebs solution was performed to eliminate residual extracts. Subsequently, acetylcholine (10 μM) was added to confirm the functional viability of the tissue, which was verified by the presence of characteristic contractile responses to this cholinergic agonist (Figure 2C, insert; Figure 2D, insert). This biphasic response may be attributed to the complex phytochemical composition of the extracts, which likely contain multiple metabolites with opposing mechanisms of action. Certain constituents may activate muscarinic receptors, thereby promoting contraction, while others may interfere with calcium influx or modulate ion channels, resulting in smooth-muscle relaxation. The coexistence of such metabolites within the same extract could explain the temporal transition between excitatory and inhibitory phases.

Among the compounds identified in the methanolic extract (ME-Ip) by UPLC-QTOF-MS are caffeic acid (3,4-dihydroxycinnamic acid), which has been shown to exert inhibitory effects on muscarinic receptors in the ileum and may also contribute to L-type calcium-channel blockade [74], and chlorogenic acid, which has demonstrated antispasmodic activity [75]. In addition, apigenin-7-O-glucoside and luteolin have been reported to inhibit KCl-induced contractions in rat ileum in a concentration-dependent manner [76]. The compound (−)-arctigenin has also been shown to relax intestinal smooth muscle, potentially through L-type calcium-channel inhibition [77]. Although the remaining metabolites detected by UPLC-QTOF-MS have not been specifically evaluated in isolated intestinal tissues, it is noteworthy that several species within the Convolvulaceae family are known to contain glycosidic resins with spasmogenic properties. For example, tricolorin A, a glycolipid isolated from *Ipomoea tricolor*, has been reported to induce spontaneous contractions in guinea pig ileum [32].

Among the metabolites identified by GC-MS analysis, heptadecanoic acid and stearic acid have been reported to induce contractions in colonic smooth muscle in rats and to increase the frequency of bowel movements [78]. Additionally, decanoic acid has been shown to dose-dependently induce muscle contraction, as characterized by contractions in isolated guinea pig duodenum and jejunum tissues [79]. Conversely, the essential oil of *Pterodon polygalaeflorus*, the main constituent of which is β-caryophyllene, has demonstrated antispasmodic activity in isolated rat ileum preparations [80]. Molecular docking studies have further suggested that γ-sitosterol possesses high binding affinity for M3 muscarinic receptors, indicating a potential antidiarrheal effect [81]. Moreover, phytol—a metabolite also isolated from *Ipomoea pes-caprae*—has exhibited antispasmodic properties in vascular smooth-muscle models [57].

Biphasic pharmacological responses, as observed in this study, have also been documented in other plant species [82,83,84,85,86]. To further substantiate the biphasic behavior of the extracts—characterized by an initial excitatory phase followed by a relaxation phase, in recordings from isolated rat ileum—additional pharmacological assays were conducted. These assays involved evaluating contractile activity in the presence of atropine (Atro, 1 μM), a selective M3 muscarinic receptor antagonist, as a control. To confirm the spasmolytic activity, both extracts were also tested on ileal tissue precontracted with KCl (38 mM). The results of these assays are presented below.

**Figure 3:**
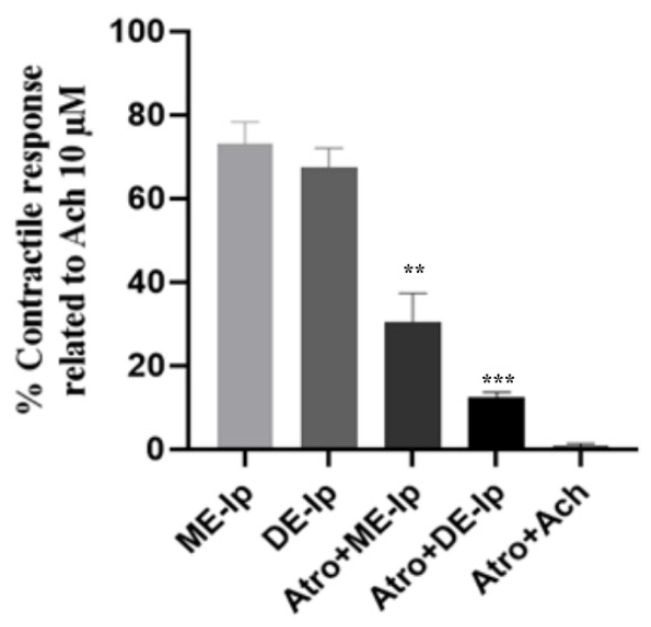
Contractile effects of ME-Ip and DE-Ip (1000 μg/mL) on the spontaneous activity of isolated rat ileum. Each bar represents the percentage of contractile response to the extract relative to that obtained with acetylcholine (Ach, 10 µM). ME-Ip: methanolic extract of aerial parts of *I. purpurea*. DE-Ip: dichloromethane extract of aerial parts of *I. purpurea*. Bars are means ± SEM, based on 3–6 experiments. Significant difference, ** *p* < 0.01, *** *p* < 0.001. Atropine (Atro, 1 μM) was used as spasmolytic control.

The co-administration of atropine with either ME-Ip or DE-Ip resulted in statistically significant reductions (*p* = 0.0049 and *p* = 0.000001, respectively) in contractility compared to treatment with the respective extracts alone. The pre-treatment with atropine significantly attenuated the contractile responses elicited by ME-Ip and DE-Ip, suggesting a potential involvement of cholinergic pathways in the mechanisms of action of the two extracts (Figure 3). These findings support the hypothesis that the observed contractile activity of ME-Ip and DE-Ip may be mediated, at least in part, through modulation of the cholinergic system [87].

**Figure 4:**
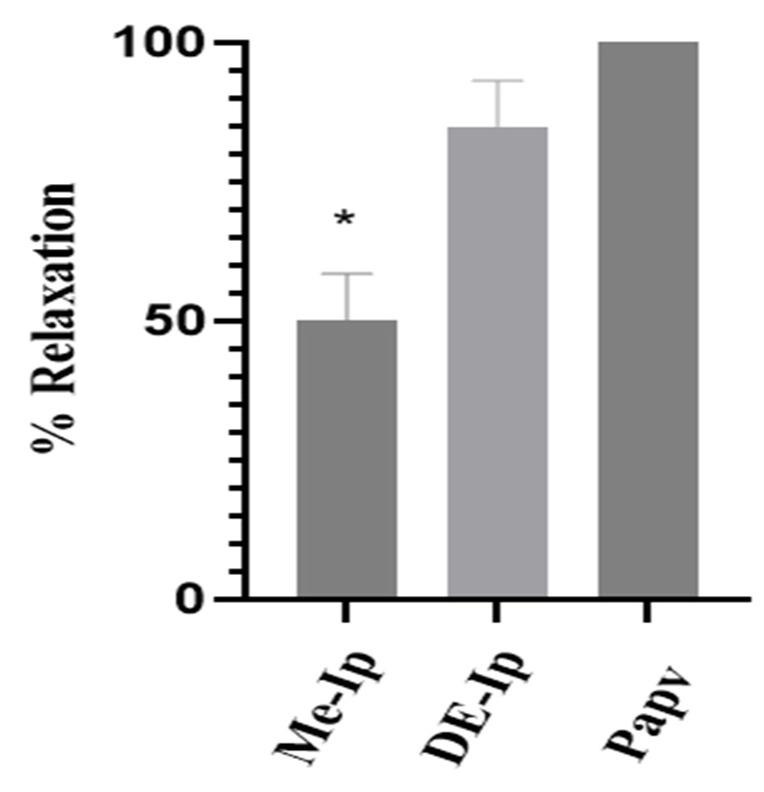
Relaxant effects of ME-Ip and DE-Ip (1000 μg/mL) on KCl (32 mM)-induced contractions. Each bar represents the percentage of relaxant response to the extract relative to KCl-induced contractions. The bars represent the mean ± SEM of 6 experiments. Papaverine (Papv, 30 µM) was used as spasmolytic control. ME-Ip: methanolic extract of aerial parts of *I. purpurea*. DE-Ip: dichloromethane extract of aerial parts of *I. purpurea*. * Significant differences (*p* < 0.05).

Additionally, the potential spasmolytic activity of the extracts was assessed using KCl-induced contractions, a model that activates voltage-dependent Ca^2+^ channels [76]. As shown in Figure 4, DE-Ip exhibited a significantly greater relaxant effect than ME-Ip (*p* < 0.05), indicating a more pronounced antispasmodic potential under the experimental conditions employed. Furthermore, the relaxant effect of ME-Ip was significantly lower (*p* < 0.05) than that of papaverine (a reference compound with well-established smooth-muscle relaxant properties), suggesting a limited spasmolytic capacity for ME-Ip. In contrast, DE-Ip demonstrated no statistically significant difference (*p* < 0.05) in relaxant activity compared to papaverine, indicating that its antispasmodic effect may be comparable to that of the positive control, at least within the evaluated concentrations and model system [88].

By comparing the antispasmodic effects of ME-Ip (EC_50_ = 0.44 mg/mL) and DE-Ip (EC_50_ = 0.34 mg/mL) with the spasmolytic activity reported for aqueous-methanolic extracts of *Matricaria chamomilla* (EC_50_ = 0.89 mg/mL) [89] and *Mentha longifolia* (EC_50_ = 1.80 mg/mL) [90] on spontaneous contractions of isolated rabbit jejunum—two plant species widely recognized for their antispasmodic properties—a comparison of relative potency reveals that ME-Ip is approximately 2- and 4-fold more potent than *M. chamomilla* and *M. longifolia* extracts, respectively. Likewise, DE-Ip exhibits a potency approximately 3- and 5-fold greater than that of *M. chamomilla* and *M. longifolia*, respectively. However, it is important to note that, according to the aforementioned studies, the aqueous-methanolic extracts of *M. chamomilla* and *M. longifolia* inhibited spontaneous contractions of isolated rabbit jejunum by approximately 100%, whereas ME-Ip and DE-Ip achieved maximum inhibitory effects (E_max_) of only 52.5% and 85.32%, respectively. These findings indicate that, despite the higher potency observed for the *I. purpurea* extracts, the aqueous-methanolic extracts of *M. chamomilla* and *M. longifolia* are more effective than those of *I. purpurea*. On the other hand, hyoscine butylbromide (also known as scopolamine butylbromide) is an antispasmodic agent widely used in the treatment of gastrointestinal spasms [91]. In isolated segments of guinea pig ileum, the application of 4–6 µg/mL to the serosal side of the tissue was sufficient to abolish peristaltic activity [92]. Based on this, the relative potency values of ME-Ip and DE-Ip were estimated to be approximately 0.014 and 0.018, respectively, when compared to scopolamine butylbromide. Accordingly, scopolamine butylbromide is between 56- and 73-fold more potent than the ME-Ip and DE-Ip extracts, respectively.

In the case of *Ipomoea purpurea*, the species has been previously reported to facilitate bowel movements (suggesting purgative activity), while also exhibiting analgesic effects [29,30]. Osmotic laxatives function by retaining water within the intestinal lumen through non-absorbable ions or molecules, whereas stimulant laxatives promote peristalsis or increase colonic secretion of water and electrolytes. For instance, species of the *Senna genus* exert a laxative effect primarily due to the presence of sennosides A and B [93,94,95,96]. Gastrointestinal disorders characterized by symptoms such as constipation and abdominal pain are frequently managed with laxative agents, reducing patient discomfort.

Mearin et al. [93] developed a Clinical Practice Guideline for Irritable Bowel Syndrome with Constipation and Functional Constipation in Adults, in which it is stated that while laxatives enhance intestinal motility, antispasmodics are employed to alleviate visceral pain [97]. Accordingly, the dual modulatory effect of *I. purpurea* extracts on intestinal smooth muscle (capable of both stimulating and relaxing activity), positions this plant species as a promising candidate for the development of phytopharmaceutical formulations proposed as adjunctive treatments for gastrointestinal disorders which combine constipation and abdominal discomfort [94,95].

### 2.7. Acute Oral Toxicity Study

The most commonly used methodologies for assessing acute toxicity are primarily aligned with the 3Rs principle (replacement, reduction, and refinement) of animal experimentation [98]. Regarding this, the Lorke method is based on the progressive evaluation of toxicity through the sequential administration of varying doses. This method comprises two distinct phases. In the first phase, the test extract is administered to three groups of animals (*n* = 3 per group) at ascending dose levels of 10, 100, and 1000 mg/kg. The animals are observed over a 24 h period for signs of toxicity or mortality. The second phase involves administering higher doses, selected according to the number of deaths observed, generally within the range identified as critical in phase one. These doses are administered to individual animals, one per dose, to improve the estimation of the median lethal dose (LD_50_), allowing preliminary information on the acute effects of a compound while minimizing animal use [99]. The LD_50_ values for ME-Ip and DE-Ip were greater than 5000 mg/kg (Table 5), indicating low levels of acute toxicity.

In both phases of the study (Table 5), piloerection was observed in animals treated with ME-Ip or DE-Ip. This physiological response may be associated with activation of the sympathetic nervous system under conditions of stress or physiological imbalance, such as the early stages of dehydration [100]. A similar response was noted in the group treated with sennosides A and B, compounds commonly used as laxatives due to their ability to enhance intestinal motility by stimulating nerve endings in the intestinal mucosa [94,95]. The stool characteristics observed after administration of ME-Ip or DE-Ip, at all doses evaluated using the Lorke method, only lasted for the first 6 h post-administration. Nevertheless, animals treated with sennosides A and B maintained these fecal characteristics for up to 24 h afterward. This coincidence suggests that ME-Ip and DE-Ip extracts may induce similar but less intense effects, compared to sennosides [95,96,97].

In addition, it has been documented that sennosides A and B can cause abdominal pain and diarrhea, decrease colonic tone, and promote alteration of natural peristalsis and electrolyte imbalances, and some studies suggest that prolonged use can stimulate epithelial hyperplasia and potentially promote early neoplastic lesions. Hence, the search for new and better pharmacological alternatives for the management of gastrointestinal disorders continues [95].

The effects of ME-Ip and DE-Ip on stool characteristics correlate with the results of the ileum assay, in which contractions were evident after exposure of the tissue to different concentrations of both extracts (Figure 4). These contractions indicate a stimulating effect on intestinal musculature, which could accelerate intestinal transit and reduce water reabsorption time, resulting in softer or moister stools. This supports the hypothesis of a purgative effect of the extracts, suggesting a potential application as a phytotherapeutic agent for cases of constipation [96,97].

No statistically significant reductions in body weight (*p* > 0.05) were observed in any of the experimental groups throughout the study duration (Table 6). These findings suggest that treatment with ME-Ip and DE-Ip extracts did not adversely affect the animals’ overall health or metabolic status.

### 2.8. Hematological Cell Profile and Biochemical Profile of Experimental Subjects Treated with the ME-Ip and DE-Ip

The hematological profiles of the experimental groups administered ME-Ip and DE-Ip revealed no evidence of cytopenias, significant alterations in red blood cell indices (*p* > 0.05), or morphological abnormalities in peripheral blood cells compared to the control group (Table 7). The absence of such changes indicates that ME-Ip and DE-Ip do not elicit acute hematopoietic toxicity.

Within the white blood cell parameters, no statistically significant differences (*p* > 0.05) were observed between the experimental and control groups, except in the lymphocyte count (Table 8). Specifically, the group treated with ME-Ip at a dose of 1000 mg/kg exhibited a statistically significant increase (*p* value = 0.005, *p* < 0.05) in lymphocyte count compared to the control group. Nonetheless, the lymphocyte values remained within the physiological range reported for the CD-1 mouse strain, and no clinical signs of toxicity were observed. Furthermore, the leukocyte differential count did not reveal any abnormalities indicative of systemic toxicity [101]. These findings suggest that, under the conditions of the present study, the administration of ME-Ip and DE-Ip does not induce a systemic inflammatory response or acute hematological alterations.

The biochemical profiles of the groups administered ME-Ip and DE-Ip showed no statistically significant differences (*p* > 0.05) compared to the control group (Table 9). Specifically, no elevations were observed in hepatic biomarkers, i.e., alanine aminotransferase (ALT) and aspartate aminotransferase (AST), or in renal function parameters, including urea and creatinine. These findings indicate that administration of ME-Ip and DE-Ip at a dose of 1000 mg/kg does not acutely compromise liver or kidney function. Typically, a toxic effect would be reflected by elevated levels of these biomarkers, which are indicative of hepatocellular injury or impaired glomerular filtration [102]. Moreover, no significant alterations were detected in serum cholesterol or total protein levels. Particularly, abnormal cholesterol levels may signal hepatic dysfunction, particularly disturbances in lipid metabolism, while a reduction in total protein concentration could suggest hepatic impairment or protein loss due to renal or gastrointestinal toxicity [103].

### 2.9. Histopathological Assessment of Experimental Subjects Treated with the ME-Ip or DE-Ip

Finally, histopathological analysis of liver and kidney tissues from experimental subjects administered ME-Ip or DE-Ip revealed no evidence of tissue lesions, necrosis, inflammatory infiltrates, or structural alterations when compared to the control group (Table 10). These findings confirm the structural and functional integrity of these primary target organs commonly assessed in acute toxicity studies [104,105,106].

From a pharmacological point of view, the absence of significant alterations in hematological, biochemical, and histopathological parameters, along with the apparent tolerance to ME-Ip and DE-Ip even at high doses, supports the safety profile of *Ipomoea purpurea* as a potential candidate for phytopharmaceutical development. The lack of adverse effects on blood chemistry and key organ histology in the treated groups suggests that these extracts do not induce acute toxicity under the conditions of this study.

Nevertheless, it is presently necessary to conduct subacute and chronic toxicity assessments to evaluate the potential effects of prolonged exposure. These studies are essential to determine whether the extracts exhibit delayed or cumulative toxic effects that may not be detectable in an acute toxicity model.

To provide a concise overview of the principal findings, a summary table (Table 11) is presented below. This table highlights the key phytochemical analyses, pharmacological effects, and relevant toxicological outcomes of the evaluated extracts, thereby facilitating an integrated interpretation of the results discussed above.

## 3. Materials and Methods

### 3.1. Reagents

Methanol (Cat. No. 9093-3), dichloromethane (Cat. No. 9324-03), sodium bicarbonate (Cat. No. 3506-01), dibasic potassium phosphate (Cat. No. 3506-05), mercuric chloride (Cat. No. 2594-04), sodium hydroxide (Cat. No. 6697), sulfuric acid (Cat. No. 9681-05), sodium molybdate (Cat. No. 3764-01), phosphoric acid reagent grade (Cat. No. 0260-03), and acetonitrile HPLC grade (Cat. No. 9012-03) were obtained from J.T. Baker (Radnor, PA, USA).

Phenylephrine (Cat. No. P1250000), acetylcholine (Cat. No. A6625), atropine (Cat. No. A0132), gallic acid (Cat. No. G7384), tannic acid (Cat. No. W304204), and 6-hydroxy- 2,5,7,8-tetramethylchromane-2-carboxylic acid (Cat. No.238813) were obtained from Sigma-Aldrich (St. Louis, MO, USA)

Bismuth nitrate (Cat. No. 101878), sodium acetate (Cat. No. 128201), ferric chloride (Cat. No. 103943), aluminum chloride (Cat. No. 237051), sodium tungstate (Cat. No. 106673), formic acid (Cat. No. 533002), 2,4,6-tris(2-pyridyl)-s-triazine (TPTZ) (Cat. No. 93285), 2,2′ -diphenil-1-picrilhidrazil (Cat. No. D9132), the Folin–Ciocalteau reagent (Cat. No. F9252), and polysorbate 80 (Cat. No. Y0002469) were obtained from Merck (Darmstadt, Germany).

Glucose (Cat. No. 1445), sodium chloride (Cat. No. 2365), potassium chloride (Cat. No. 0455), hydrochloric acid (Cat. No. 0095), nitric acid (Cat. No. 0195), and acetic anhydride (Cat. No. 0652) reagent grade were purchased from Meyer (Mexico City, Mexico)

Magnesium sulphate (Cat. No. 50961), potassium iodide (Cat. No. 28586), chloroform (Cat. No. 50930), cholesterol (Cat. No. 24913), and sodium carbonate (Cat. No. 50887) were purchased from CTR Scientific (Monterrey, Mexico).

### 3.2. Vegetal Material

Aerial parts of *Ipomoea purpurea* were collected in Cadereyta de Montes, Querétaro México, in September 2023 by Biologist Beatriz Maruri-Aguilar and M.Sc. Yazmin Hailen Ugalde de la Cruz. Botanical identification was carried out in the Jerzy Rzedowsky Herbarium, Natural Sciences Department, Autonomous University of Querétaro (voucher number QMEX00007403, *Ipomoea purpurea* L. Roth).

### 3.3. Experimental Animals

All experimental procedures were conducted in accordance with the guidelines established by the Mexican Official Standard NOM-062-ZOO-1999 [107], and were approved by the Bioethics Committee of the Chemistry Department, Autonomous University of Querétaro. The protocol received ethical approval from the same committee under the approval code CBQ24/020. Male Wistar rats (250–300 g) and CD-1 male mice (25–30 g) were obtained from the Institute of Neurobiology, National Autonomous University of Mexico, Juriquilla Campus.

Animals were housed in standard cages under controlled temperature conditions (22 °C) with a 12:12 h light–dark cycle. Food and water were provided ad libitum. Waste disposal was carried out in accordance with the Mexican Official Standard NOM-087-SEMARNAT-SSA1-2002 [108].

### 3.4. Preparation of Dichloromethane and Methanol Extracts of I. purpurea

Plant samples were air dried at room temperature and ground to a powder with a hand mill (Gilson, Co., Inc., LC-80, Lewis Center, OH, USA). Soxhlet extraction was carried out under standardized conditions, with the operating temperature adjusted according to the boiling point of the selected solvents (65 °C for methanol and 39–40 °C for dichloromethane). A solvent-to-plant material ratio range of 10:1 (*v*/*w*) was employed, using dried plant material with a particle size of approximately 0.5–2 mm to enhance extraction efficiency. The extraction process was conducted over a period of 4 h, allowing a solvent recirculation rate of one cycle every 10 min, which resulted in approximately 24 extraction cycles in total. Then, the solvents were removed with a rotatory evaporator (Heidolph^®^, VV 2000, SCHB, Schwabach, Germany), at temperatures below 45 °C to avoid thermal degradation of heat-sensitive phytoconstituents, in order to obtain dry extracts.

### 3.5. Phytochemical Screening

Qualitative assays were carried out on the methanol extract of *I. purpurea* (ME-Ip) and dichloromethane extract of *I. purpurea* (DE-Ip) in order to identify the presence of diverse groups of metabolites such as alkaloids, phenolic compounds, flavonoids, tannins, terpenoids, saponins, glycosides, anthraquinones, and carbohydrates, according to the methods described previously [109,110,111,112].

### 3.6. Phenolic Compound Content in Methanolic Extract

Total phenolic compounds were assessed using the Folin–Ciocalteu method according to the description in [113]. The content of phenolic compounds was calculated using a standard curve of gallic acid in a concentration range of 5 to 100 µg/mL, and the results are expressed as gallic acid equivalents, as mg of AG/g of extract.

### 3.7. Flavonoid Content in Methanolic Extract

Flavonoid content was evaluated by Zhishen’s method [113]. The calibration curve was prepared from 20 to 200 µg/mL with a standard curve of (+) catechin, and the content of flavonoids in the extracts was expressed in (+) catechin equivalent, as mg of CA/g of extract.

### 3.8. Determination of Antioxidant Capacity

The antioxidant capacity (AOC) was carried out by two assays: the 1,1-diphenyl-2-picrylhydrazyl (DPPH) radical scavenging method and iron-reducing antioxidant power (FRAP) assay [114]. The AOCs obtained from both methods are expressed as µmol Trolox equivalents/g of extract.

### 3.9. UPLC–QTOF-MS Analysis of Methanolic Extract

In order to identify the metabolites in the ME-Ip, an ultra-high-performance liquid chromatography quadrupole time-of-flight mass spectrometry study was carried out. The sample was dissolved in methanol (1.5 mg/mL), filtered using a syringe filter (0.45 µm), and 6 µL was injected into the UPLC system. This analysis was performed using an Acquity UPLC I-class (Waters Co., Milford, CT, USA) with a diode array detector (DAD) set at 210 to 600 nm, and an XBride BEH amide C_18_ reverse phase column (2.1 mm × 150 mm, 2.5 µm; Waters Co., Milford, CT, USA). The mobile phase was a mixture of 0.1% formic acid in water and acetonitrile at a flow rate of 0.4 mL/min and 35 °C. The UPLC was coupled to a mass spectrometer detector equipped with an electrospray ionization (ESI) interphase source operating in the negative ionization mode (Vion; Waters Co, Milford, USA). The analysis mode was MSE, and the low collision energy was 6 eV, with a ramp from 15 to 45 eV at high energy. The mass range was considered from 50 to 1800 *m*/*z*. The capillary voltage used was 2 kV for negative; the source temperature, 120 °C; the desolvation temperature, 450 °C; argon was utilized as the collision and desolvation gas, with flow rates of 50 L/h and 800 L/h, respectively; and cone voltage was 40 V. Leucine enkephalin was used at a concentration of 200 pg/µL as a reference for mass correction, with a flow rate of 10 µL/min.

Data analysis was performed using Unifi 1.9 SR 4 software with libraries from the Specialized Food Analysis Laboratory of the Chemistry Department of the Autonomous University of Queretaro, the University of Mississippi Botanical Library, and the University of Ottawa Phytochemical Library. The target match tolerance was set at 5 ppm. Fragment identification was performed by comparing fragmentation patterns reported in PubChem, FooDB version 1.0, HMDB version 5.0, and Mass Bank of North America, as well as those from previous analyses performed in the laboratory. For compounds without a reported fragmentation pattern, an in silico fragmentation pattern was generated by the software, with a tolerance of 10 mDa.

### 3.10. GC-MS Analysis of Dichloromethane Extract of Ipomoea purpurea

Gas chromatography separation and quantification was carried out using an Agilent GC 7890A series instrument (Agilent Technologies, Inc., Santa Clara, CA, USA) coupled with an Agilent 5975C Mass Spectrometer in electron impact mode (EI) and a quadrupole analyzer. The injector was operated in 1:5 split mode; the injection port and detector temperatures were 280 °C. The capillary columns used were an HP-5MS (60 m × 250 um i.d., 0.25 μm film thickness; Agilent Technologies, Inc., Santa Clara, CA, USA). The oven was programmed to produce an initial temperature of 40 °C for 10 min, then raise the temperature at 3 °C/min to 140 °C and hold for 20 min, then raise it at 3 °C/min to 220 °C and hold for 5 min, and finally to raise it at 10 °C/min to 270 °C and hold for 15 min. Helium was used as the carrier gas, at a constant flow rate of 1 mL/min. The Agilent 5975C Mass Spectrometer detector (Agilent Technologies, Inc., Santa Clara, CA, USA) was used in electron impact mode (EI), with an ionization voltage of 70 eV and gain factor of 1. The temperatures of the transfer line, ion source, and quadrupole were 280 °C, 230 °C, and 150 °C, respectively. Full scan mode was used in a range of 33–600 uma. Identification of volatile compounds was performed by comparing their mass spectra with those in the NIST Mass Spectral Library (National Institute of Standards and Technology, Gaithersburg, MD, USA, 2023 version).

### 3.11. Isolated Rat Aorta Assay

The isolated rat aorta assay was employed to evaluate the vasodilatory activity of both extracts obtained from *Ipomoea purpurea*, following previously described methods [115,116,117].

Rats were euthanized by decapitation in accordance with NOM-062-ZOO-1999 (Section 3.3). The thoracic aorta was excised and immediately placed in cold Krebs–Henseleit solution composed of (in mM) 126.8 NaCl, 5.9 KCl, 1.2 KH_2_PO_4_, 1.2 MgSO_4_, 5.0 D-glucose, 30 NaHCO_3_, and 2.5 CaCl_2_ (pH 7.4), and continuously bubbled with carbogen (95% O_2_ and 5% CO_2_). The lumen of the aorta was flushed with Krebs solution to prevent clot formation and surrounding adipose and connective tissues were carefully removed. Aortic rings measuring 4–5 mm in length were then prepared and mounted in 5 mL organ baths containing pre-warmed Krebs–Henseleit solution maintained at 37 °C and aerated with carbogen. Tissues were equilibrated for 30 min under tension of 1.5 g, with the bathing solution replaced every 10 min. To assess contractile responsiveness, tissues were first stimulated with 100 mM KCl. Once a stable contraction was observed, the solution was replaced to restore basal tone. Subsequently, tissues were challenged with 1µM L-phenylephrine. The contractile response induced by L-phenylephrine was defined as 100%, and once a stable contractile plateau was reached, the extracts were tested cumulatively (1, 3.16, 10, 31.6, 100, 316, 1000, 3600 10,000 µg/mL). Acetylcholine was used as a positive control.

Changes in vascular tension were measured using Grass FT03 force transducers connected to a Grass 7D Polygraph (Grass Instrument Co., Quincy, MA, USA). Vasorelaxation responses induced by the extracts were expressed as percentages of relaxation relative to the maximum contraction elicited by L-phenylephrine [115,116,117].

### 3.12. Isolated Rat Ileum Assay

Rats were euthanized by decapitation in accordance with the guidelines established in NOM-062-ZOO-1999 (Section 3.3). Ileum segments were immediately excised and placed in an organ isolation chamber containing Krebs solution maintained at 37 °C and continuously aerated with carbogen (95% O_2_, 5% CO_2_). Following an initial stabilization period of 10 min, the tissues were incubated for an additional 10 min with the test substances (extracts and controls). Changes in isometric tension in response to increasing concentrations of the test substances (1, 3.16, 10, 31.6, 100, 316 and 1000 µg/mL) were recorded using Grass FT03 force transducers connected to a Grass 7D polygraph (Grass Instrument Co., Quincy, MA, USA) [118]. At the end of each recording, the tissue was washed with Krebs–Henseleit solution; this was followed by the addition of acetylcholine to verify tissue viability.

To assess the spasmogenic activity of *Ipomoea purpurea* extracts, the contractile responses were compared to the maximal contraction induced by acetylcholine (10 µM), which was defined as 100% [119]. To investigate the potential involvement of muscarinic receptors in the observed spasmogenic effects, a separate set of experiments was conducted in the presence of atropine (1 µM) [119]. The potential spasmolytic effect of the extracts (1000 µg/mL) was evaluated in tissues precontracted with 32 mM KCl for at least five minutes. Relaxant responses were assessed over a five-minute period, or until the maximum relaxation was maintained for a minimum of 10 s. Papaverine (30 µM) was used as a positive control [118,119].

### 3.13. Acute Oral Toxicity Study

This study was conducted in accordance with the acute toxicity assay described by Lorke (1983) [99], using CD-1 mice. Prior to treatment, animals were fasted overnight and provided access to food one hour pre-administration. In the acute toxicity assessment following the Lorke method, key clinical signs were monitored in the rodents, including ocular changes (such as secretion, opacity, or mydriasis), piloerection, lethargy, somnolence, tremors, excessive salivation, alterations in fecal consistency and appearance, mucosal conditions (pallor or erythema), and neurological signs such as coma. These observations were conducted at regular intervals during the first 24 h and daily over a 14-day period to identify sublethal or systemic adverse effects.

The methanolic extract of *Ipomoea purpurea* (ME-Ip) was dissolved in water, while the dichloromethane extract (DE-Ip) was dissolved in a mixture of Tween 80 and water. Mice were subjected to a two-phase treatment protocol and body weights were recorded on days 1, 7, and 14.

Phase I:

Three dose levels (10, 100, and 1000 mg/kg) of ME-Ip and DE-Ip, as well as saline solution (negative control), were administered orally via gavage to groups of nine mice per dose. An additional group of three mice received only saline solution as the control group. After a single administration, animals were monitored for signs of toxicity and mortality during the first 24 h, and subsequently observed daily for 14 days. Clinical signs such as changes in skin, fur, eyes, respiratory function, and behavior were recorded. At the end of the observation period, all animals were euthanized by decapitation and their organs were dissected for gross pathological examination to identify potential signs of toxicity [120].

Phase II:

Higher dose levels (1600, 2900, and 5000 mg/kg) of ME-Ip and DE-Ip, along with saline solution, were administered by gavage to groups of four mice per dose. Animals were observed for toxicological signs during the first 24 h following administration. At the end of the observation period, all animals were euthanized by decapitation (Lorke, 1983) [99] and their organs were dissected for macroscopic evaluation of possible toxic effects [121].

Lethal dose media (LD_50_) was calculated as follows:

$${\mathrm{LD}}_{50}\;=\;\sqrt{\left(D_{0\;}\times \;D_{100}\right)}$$

D_0_ = Highest dose that does not produce mortality

D_100_ = Lowest dose that produce mortality

### 3.14. Fecal Assessment

An assessment of fecal deposition was conducted to evaluate whether *Ipomoea purpurea* extracts exert a purgative effect in experimental animals. Fecal output was monitored during four consecutive 2 h intervals (0–2 h, 2–4 h, 4–6 h, and 6–8 h) following administration of the two extracts—methanolic extract of *I. purpurea* (ME-Ip) and dichloromethane extract of *I. purpurea* (DE-Ip)—at the concentrations specified in the Phase 1 acute toxicity study. A saline solution was used as the negative control, while sennosides A and B (2 mg/mL, 1 mL/kg) served as the positive control. Fecal consistency was classified based on stool morphology: normal stools were defined as brown, rugby ball-shaped pellets, whereas abnormal stools were characterized as loose, cloudy, or watery. Observations were conducted by a veterinary evaluator blinded to the treatment groups [96].

### 3.15. Hematological Cell Profile, Biochemical Profile and Histopathological Analysis

After a 14-day observation period, blood, kidney, and liver samples were collected from animals administered the highest doses in both experimental phases—Phase 1: 1000 mg/kg and Phase 2: 5000 mg/kg—as well as from the control group. Prior to tissue collection, the animals were anesthetized using carbon dioxide (CO_2_), and blood was obtained via cardiac puncture. The collected blood was divided into two tubes: one without additives and the other containing EDTA K_2_ as an anticoagulant. The liver and kidneys were excised, weighed, and immediately fixed in 10% neutral buffered formalin. A macroscopic examination was performed to assess the presence, in comparison with the control group, of gross pathological alterations, such as lesion development, attributable to exposure to ME-Ip and DE-Ip extracts. Following fixation, the tissues were processed using standard paraffin-embedding techniques. A complete blood count (CBC) was conducted using an automated hematology analyzer (Gilson Co. Inc., LC-80, Lewis Center, OH, USA). Serum was obtained by centrifuging the additive-free blood samples at 5000 rpm for 5 min. Biochemical parameters, including urea, creatinine, alanine aminotransferase (ALT), aspartate aminotransferase (AST), alkaline phosphatase (ALP), cholesterol, and total protein, were measured spectrophotometrically using a biochemistry analyzer (Mindray BS-360E, Shenzhen, China).

### 3.16. Statistical Analysis

Total phenolic content, flavonoid concentration, and antioxidant capacity were determined in triplicate, and the results are expressed as the mean ± standard deviation (SD). Ex vivo assays were performed in sextuplicate, and the results are reported as the mean ± standard error of the mean (SEM). Experimental data were fitted to a sigmoidal equation, plotted, and analyzed to calculate EC_50_ and E_max_ values using GraphPad Prism 7.02 (GraphPad Software, San Diego, CA, USA). For the analysis of isolated rat aorta, statistical comparisons were conducted using one-way ANOVA followed by Tukey’s post hoc test. Statistical significance was accepted at the 95% confidence level (*p* < 0.05). For the evaluation of basal contractions of isolated rat aorta, the investigation of spasmogenic and antispasmodic mechanisms, animal and organ weight, hematological parameters (red and white blood cell profiles), and biochemical profiles, an unpaired Student’s t-test was used. Statistical significance was set at *p* < 0.05. All statistical analyses were performed using the aforementioned software.

## 4. Conclusions

This study proved the presence of secondary metabolites in the methanolic and dichloromethane extracts of aerial parts of *I. purpurea*, similar to those detected in other species of genus *Ipomoea*, as expected due to the chemotaxonomic relationships. The ME-Ip showed important levels of total phenolic content and antioxidant capacity in comparison to other species of genus *Ipomoea*; this can be attributed mainly to presence of phenolic acids and flavonoids. The presence of apigenin-7-glucuronide and diosmetin 7-O-β-D-glucopyranoside is reported for the first time in *I. purpurea*. The major chemical compounds identified in DE-Ip include carboxylic acids, terpenoids, esters, and alkanes. The Me-Ip and DE-Ip both exhibited a slight vasodilatory effect on the isolated rat aorta assay; both extracts elicited a biphasic effect (contractile and relaxant) on intestinal smooth muscle. The absence of significant alterations in hematological, biochemical, and histopathological parameters resulting from the acute toxicological evaluation constitutes a preliminary step in the toxicological safety assessment of *I. purpurea* extracts and partly supports the safety of *I. purpurea* as a potential candidate for phytopharmaceutical development. Further studies are needed to evaluate subacute and chronic toxicity, and to explore additional pharmacological effects of *Ipomoea purpurea*. Bioactivity-guided fractionation and mechanistic studies are also recommended in order to identify active compounds and elucidate their vasodilatory and spasmolytic mechanisms.

## Figures and Tables

**Table 1:** Phytochemical tests of methanolic extract (ME-Ip) and dichloromethane extract (DE-Ip) of *Ipomoea purpurea* for the presence of secondary metabolites.

Type of Phytochemical Constituent	Name of the Test	Methanolic Extract of *Ipomoea purpurea* (ME-Ip)	Dichloromethane Extract of *Ipomoea purpurea* (DE-Ip)
Alkaloids	Mayer’s test	Absent	Absent
	Wagner’s test	Absent	Absent
	Dragendorff’s test	Absent	Absent
Phenolic compounds	FeCl_3_ test	Present	Absent
Flavonoids	NaOH Test	Present	Absent
	Shinoda’s test	Present	Absent
Terpene compounds and phytosterols	Liebermann–Burchard test	Absent	Present
	Salkowski’s test	Absent	Present
Tannins	FeCl_3_ test	Present	Absent
Saponins	Foam test	Present	Present
Glycosides	Kiliani Keller’s test	Present	Present
	Borntager’s test	Present	Present
Carbohydrates	Molish’s test	Present	Present

**Table 2:** Total phenolic content and antioxidant capacity of methanolic extract of *Ipomoea purpurea* (ME-Ip).

Method	Result
Determination of total phenolic compounds (Folin-Ciocalteau method)	38.69 ± 0.01 mg GAE/g ^1^ dry extract
Determination of total flavonoids (Zhishen method)	17.86 ± 0.01 mg CAE/g ^2^ dry extract
FRAP method ^3^	12.90 ± 0.12 μmol TE/g ^4^ dry extract
DPPH method ^5^	19.2 ± 2.85% ^6^

^1^ Total phenolic compounds, expressed as gallic acid equivalents. ^2^ Total flavonoids, expressed as catechin equivalents. ^3^ Iron-reducing antioxidant power. ^4^ Antioxidant capacity, expressed as Trolox equivalents. ^5^ 1,1-diphenyl-2-picryl-hydrazyl. ^6^ Antioxidant capacity, expressed as percentage of inhibition. All values are mean ± SD.

**Table 3:** Identification of compounds in methanolic extract of aerial parts of *Ipomoea purpurea* (ME-Ip), by UPLC-MS analysis.

Proposed Compound	R_t_ (min)	[M–H]^−^ (Parent Ion)	Theoretical Neutral Mass (Da)	Experimental Neutral Mass (Da)	Error (ppm)	MS/MS Fragments (*m*/*z*)	Relative Response
Caffeic acid 4-O-glucoside	4.09	341.08774	342.0951	342.0950	0.2	297.03008, 235.05643	3931
Astragalin	7.00	447.0933	448.1006	448.1006	0.0	283.02514, 255.02728, 446.08195	1456
Caffeic acid	4.45	179.03447	180.04226	179.0345	2.9	135.04485, 133.02856, 93.03396	10772
Chlorogenic acid	6.63	353.06627	354.09508	354.0952	0.2	133.02923, 191.05538, 135.04495	12682
Ferulic acid	4.01	193.05000	194.05791	193.0504	1.4	177.01853, 93.03412, 137.0236	1190
N-cis-feruloyltyramine	5.02	312.12398	313.13141	312.1240	0.5	135.04415, 178.05017, 297.1091	13242
Apigenin 7-O-glucoside	6.01	431.0987	432.10565	432.1080	0.8	311.05594, 283.06081, 269.04455	92404
Apigenin 7-glucuronide	5.47	445.0773	446.08401	445.0773	0.7	427.21744, 327.05087, 269.04547	2342
3,4-Di-O-caffeoylquinic acid	7.72	515.11909	530.14243	529.1353	0.2	191.05538, 375.07003,353.08788	3879
5Z-Caffeoylquinic acid	4.45	353.08827	354.09508	353.0883	1.3	135.04485, 179.03447	52663
Diosmetin 7-O-β-D-glucopyranoside	7.95	461.10852	462.1162	462.1160	0.9	311.05632, 341.10154, 446.16078	1052
(−)-Arctigenin	23.25	371.14895	372.15729	371.1490	2.9	269.24585, 339.15959	1081
Betulinic acid	18.43	455.35226	456.36035	455.3523	0.8	275.19835, 407.17605, 425.169	3025

**Table 4:** Identification of compounds in dichloromethane extract of aerial parts of *Ipomoea purpurea* (DE-Ip), by GC-MS analysis.

Type of Metabolite	Metabolite	Condensed Formula	Area (%)	R_t_ ^1^	RI_e_ ^2^	RI_b_ ^3^	Reference
Carboxylic acid	Acetic acid	C_2_H_4_O_2_	0.05	5.784	601.1	610	a,b
Carboxylic acid	Octanoic acid	C_8_H_16_O_2_	1.42	38.456	1041	1182	a,b
Carboxylic acid	N-decanoic acid	C_10_H_20_O_2_	0.75	47.860	1163	1376	a,b
Carboxylic acid	Tetradecanoic acid	C_14_H_28_O_2_	0.08	79.902	1901	1787	a,b
Carboxylic acid	n-Hexadecanoic acid	C_16_H_32_O_2_	7.80	89.042	1955	1981	a,b
Carboxylic acid	Heptadecanoic acid	C_17_H_34_O_2_	0.05	92.453	1972	2022	a,b
Carboxylic acid	8,11-Octadecadienic acid	C_18_H_32_O_2_	0.12	93.638	1977	2159	a,b
Carboxylic acid	Linolenic acid	C_19_H_32_O_2_	0.13	93.901	1977	2107	a,c
Carboxylic acid	Linoleic acid	C_18_H_32_O_2_	2.55	95.749	1989	2095	a,c
Carboxylic acid	Stearic acid	C_18_H_36_O_2_	1.99	96.734	1996	2157	a,b
Carboxylic acid	Arachidic acid	C_20_H_40_O_2_	0.78	101.194	2015	2359	a,b
Ester	Methyl palmitate	C_17_H_34_O_2_	0.07	87.156	1943	1913	a,b
Ester	Methyl 7,10,13-hexadecatrienoate	C_17_H_28_O_2_	5.23	96.006	1989	2352	a,c
Ester	Methyl eicosanoate	C_21_H_42_O_2_	0.13	100.499	2013	2332	a,b
Ester	Di-n-octyl phthalate	C_24_H_38_O_4_	0.17	104.052	2030	2507	a,b
Ester	Methyl hexacosanoate	C_27_H_54_O_2_	0.51	110.997	2061	2904	a,c
Ester	Hexadecyl hexadecanoate	C_32_H_64_O_2_	0.19	122.364	2110	2188	a,b
Ester	4,8,12,16-Tetramethylheptadecan-4-olide	C_21_H_40_O_2_	0.35	100.911	2013	2364	a,c
Ketone	2-Heptacosanone	C_27_H_54_O	0.79	110.798	2059	-	a,e
Ketone	2-Nonacosanone	C_29_H_58_O	0.36	115.072	2080	-	a,e
Amide	Hexadecanamide	C_16_H_33_NO	0.15	97.274	1997	2182	a,c
Amide	Tetradecanamide	C_14_H_29_NO	0.12	101.632	2018	1921	a,c
Amide	Erucamide	C_22_H_43_NO	0.35	108.545	2051	2625	a,c
Terpenoid	Caryophyllene	C_15_H_24_	0.04	51.445	1684	1464	a,b
Terpenoid	Dihydroactinidiolide	C_11_H_16_O_2_	0.15	61.795	1775	1519	a,c
Terpenoid	Spathulenol	C_15_H_24_O	0.24	66.990	1815	-	a,e
Terpenoid	Caryophyllene oxide	C_15_H_24_O	0.45	67.479	1817	-	a,e
Terpenoid	Isophytol	C_20_H_40_O	0.04	87.954	1947	1945	a,c
Terpenoid	Phytane	C_20_H_42_	0.05	89.969	1960	1791	a,c
Terpenoid	Phytol	C_20_H_40_O	0.35	94.506	1982	2119	a,c
Terpenoid	Squalene	C_30_H_50_	0.70	108.982	2052	2819	a,c
Terpenoid	γ-Tocopherol	C_28_H_48_O_2_	1.47	113.643	2074	2987	a,c
Terpenoid	α-Tocopherol	C_29_H_50_O_2_	0.42	115.477	2953	3111	a,c
Terpenoid	Campesterol	C_28_H_48_O	0.24	118.663	2095	-	a,e
Terpenoid	γ-Sitosterol	C_29_H_50_O	2.36	121.424	2104	3320	a.c
Alkene	1-Decene	C_10_H_20_	0.05	80.893	1907	982	a.b
Alkene	3,7,11,15-Tetramethyl-2-hexadecene	C_20_H_40_	0.18	83.024	1920	1830	a,b
Alkene	2,6,10,14-Tetramethyl-2-hexadecene	C_20_H_40_	0.61	83.622	1923	1855	a,c
Alkane	Eicosane	C_20_H_42_	0.83	99.958	2010	2000	a,b
Alkane	Tetracosane	C_24_H_50_	12.75	102.790	2024	2400	a,b
Alkane	Heptacosane	C_27_H_56_	0.67	107.090	2045	2700	a,d
Alkane	Octacosane	C_28_H_58_	0.41	108.706	2051	2800	a,d
Alkane	Docosane	C_22_H_46_	0.37	109.748	2055	2200	a.b
Alkane	Hentriacontane	C_31_H _64_	3.15	110.431	2058	-	a,e
Alkane	Tetratriacontane	C_34_H_70_	0.95	112.297	2067	3400	a,c
Alkane	Heneicosane	C_21_H_44_	8.68	120.214	2100	2100	a,b
Alcohol	2-Methyl-3-buten-2-ol	C_5_H_10_O	0.05	5.964	626	614	a,c
Phenol	2-Methoxy-4-vinylphenol	C_9_H_10_O_2_	0.11	44.822	1615	1325	a,c
Phenol	3-[(2-Metil-5-nitro-fenilimino)-metil]-fenol	C_14_H_12_N_2_O_3_	0.07	90.831	1963	-	a,e

a. Retention index data reported by The NIST Mass Spectral Search Program for the NIST/EPA/NIH EI and NIST Tandem Mass Spectral Library, Version 2.3, build 4 May 2017, prepared by the NIST Mass Spectrometry Data Center, Gaithersburg, MD, USA. b. Retention index data reported by the National Center for Biotechnology Information, PubChem database, National Library of Medicine, Bethesda, MD, USA. c. Retention index data reported by Database of Insect Pheromones and Semiochemicals. d. Retention index of bibliographic references. e. Unreported retention index data. ^1^ Retention time. ^2^ Experimental retention index. ^3^ Bibliographic retention index.

**Table 5:** Summary of general observations and behavioral responses in mice administered ME-Ip and DE-Ip, in comparison with the control group, during the acute toxicity assessment.

Group	Time	Eye Changes ^1^	Bristling Skin and Fur	Lethargy	Sleep	Stool	Coma	Tremors	Mucous Membrane	Salivation
Negative control	0–2 h	NC	NO	NO	Normal	Firm, brown, stringy stools.	NO	NO	NC	NO
Negative control (water)	2–4 h	NC	NO	NO	Normal	Firm, brown, stringy stools.	NO	NO	NC	NO
Negative control (water)	4–6 h	NC	NO	NO	Normal	Firm, brown, stringy stools.	NO	NO	NC	NO
Negative control (water)	24 h	NC	NO	NO	Normal	Firm, brown, stringy stools.	NO	NO	NC	NO
Positive control (Sennosides A and B)	0–2 h	NC	O	NO	Normal	Soft, moist, light brown, semi-pasty stools.	NO	NO	NC	NO
Positive control (Sennosides A and B)	2–4 h	NC	O	NO	Normal	Soft, moist, light brown, semi-pasty stools.	NO	NO	NC	NO
Positive control (Sennosides A and B)	4–6 h	NC	O	NO	Normal	Soft, moist, light brown, semi-pasty stools.	NO	NO	NC	NO
Positive control (Sennosides A and B)	24 h	NC	O	NO	Normal	Soft, moist, light brown, semi-pasty stools.	NO	NO	NC	NO
ME-Ip 10 mg/Kg	0–2 h	NC	O	NO	Normal	Soft, moist, light brown, semi-pasty stools.	NO	NO	NC	NO
ME-Ip 10 mg/Kg	2–4 h	NC	O	NO	Normal	Soft, moist, light brown, semi-pasty stools.	NO	NO	NC	NO
ME-Ip 10 mg/Kg	4–6 h	NC	O	NO	Normal	Soft, moist, light brown, semi-pasty stools.	NO	NO	NC	NO
ME-Ip 10 mg/Kg	24 h	NC	NO	NO	Normal	Firm, brown, stringy stools.	NO	NO	NC	NO
ME-Ip 100 mg/Kg	0–2 h	NC	O	NO	Normal	Soft, moist, light brown, semi-pasty stools.	NO	NO	NC	NO
ME-Ip 100 mg/Kg	2–4 h	NC	O	NO	Normal	Soft, moist, light brown, semi-pasty stools.	NO	NO	NC	NO
ME-Ip 100 mg/Kg	4–6 h	NC	O	NO	Normal	Soft, moist, light brown, semi-pasty stools.	NO	NO	NC	NO
ME-Ip 100 mg/Kg	24 h	NC	NO	NO	Normal	Firm, brown, stringy stools.	NO	NO	NC	NO
ME-Ip 1000 mg/Kg	0–2 h	NC	O	NO	Normal	Soft, moist, light brown, semi-pasty stools.	NO	NO	NC	NO
ME-Ip 1000 mg/Kg	2–4 h	NC	O	NO	Normal	Soft, moist, light brown, semi-pasty stools.	NO	NO	NC	NO
ME-Ip 1000 mg/Kg	4–6 h	NC	O	NO	Normal	Soft, moist, light brown, semi-pasty stools.	NO	NO	NC	NO
ME-Ip 1000 mg/Kg	24 h	NC	NO	NO	Normal	Firm, brown, stringy stools.	NO	NO	NC	NO
ME-Ip 5000 mg/Kg	0–2 h	NC	O	NO	Normal	Soft, moist, light brown, semi-pasty stools.	NO	NO	NC	NO
ME-Ip 5000 mg/Kg	2–4 h	NC	O	NO	Normal	Soft, moist, light brown, semi-pasty stools.	NO	NO	NC	NO
ME-Ip 5000 mg/Kg	4–6 h	NC	O	NO	Normal	Soft, moist, light brown, semi-pasty stools.	NO	NO	NC	NO
ME-Ip 5000 mg/Kg	24 h	NC	NO	NO	Normal	Firm, brown, stringy stools.	NO	NO	NC	NO
DE-Ip 10 mg/Kg	0–2 h	NC	O	NO	Normal	Soft, moist, light brown, semi-pasty stools.	NO	NO	NC	NO
DE-Ip 10 mg/Kg	2–4 h	NC	O	NO	Normal	Soft, moist, light brown, semi-pasty stools.	NO	NO	NC	NO
DE-Ip 10 mg/Kg	4–6 h	NC	O	NO	Normal	Soft, moist, light brown, semi-pasty stools.	NO	NO	NC	NO
DE-Ip 10 mg/Kg	24 h	NC	NO	NO	Normal	Firm, brown, stringy stools.	NO	NO	NC	NO
DE-Ip 100 mg/Kg	0–2 h	NC	O	NO	Normal	Soft, moist, light brown, semi-pasty stools.	NO	NO	NC	NO
DE-Ip 100 mg/Kg	2–4 h	NC	O	NO	Normal	Soft, moist, light brown, semi-pasty stools.	NO	NO	NC	NO
DE-Ip 100 mg/Kg	4–6 h	NC	O	NO	Normal	Soft, moist, light brown, semi-pasty stools.	NO	NO	NC	NO
DE-Ip 100 mg/Kg	24 h	NC	NO	NO	Normal	Firm, brown, stringy stools.	NO	NO	NC	NO
DE-Ip 1000 mg/Kg	0–2 h	NC	O	NO	Normal	Soft, moist, light brown, semi-pasty stools.	NO	NO	NC	NO
DE-Ip 1000 mg/Kg	2–4 h	NC	O	NO	Normal	Soft, moist, light brown, semi-pasty stools.	NO	NO	NC	NO
DE-Ip 1000 mg/Kg	4–6 h	NC	O	NO	Normal	Soft, moist, light brown, semi-pasty stools.	NO	NO	NC	NO
DE-Ip 1000 mg/Kg	24 h	NC	NO	NO	Normal	Firm, brown, stringy stools.	NO	NO	NC	NO
DE-Ip 5000 mg/Kg	0–2 h	NC	O	NO	Normal	Soft, moist, light brown, semi-pasty stools.	NO	NO	NC	NO
DE-Ip 5000 mg/Kg	2–4 h	NC	O	NO	Normal	Soft, moist, light brown, semi-pasty stools.	NO	NO	NC	NO
DE-Ip 5000 mg/Kg	4–6 h	NC	O	NO	Normal	Soft, moist, light brown, semi-pasty stools.	NO	NO	NC	NO
DE-Ip 5000 mg/Kg	24 h	NC	NO	NO	Normal	Firm, brown, stringy stools.	NO	NO	NC	NO

ME-Ip: Methanolic extract of aerial parts of *I. purpurea.* DE-Ip: Dichloromethane extract of aerial parts of *I. purpurea.* ^1^ Eye changes such as burning, itching, or watering of the eyes. O: Observed. NO: Not observed. NC: No change. *n* = 3 per group in ME-Ip, DE-Ip, and controls (saline solution and sennosides A and B); *n* = 1 in ME-IP 5000 mg/kg and DE-IP 5000 mg/kg, according to the Lorke method.

**Table 6:** Average body weight gain (g) of mice treated with different doses of ME-Ip and DE-Ip, compared with control group, during the acute toxicity study.

Group	Dose (mg/kg)	Day 0	Day 7	Day 14
Control	0	28.33 ± 0.58	33.67 ± 2.52	34.67 ± 2.52
ME-Ip	10	31.33 ± 1.15	35.33 ± 0.58	37.33 ± 0.58
ME-Ip	100	31.33 ± 1.15	33.67 ± 1.15	37.00 ± 1.73
ME-Ip	1000	30.67 ± 0.58	32.33 ± 1.15	34.67 ± 2.31
DE-Ip	10	29.67 ± 1.15	32.33 ± 2.31	33.67 ± 2.08
DE-Ip	100	30.00 ± 3.46	33.00 ± 2.65	34.33 ± 2.52
DE-Ip	1000	30.67 ± 2.08	33.67 ± 2.52	35.33 ± 2.08

ME-Ip: Methanolic extract from aerial parts of *I. purpurea.* DE-Ip: Dichloromethane extract from aerial parts of *I. purpurea.* Values expressed as mean ± standard deviation (*n* = 3 per group). The weights (g) of the mice in the treatment groups at days 0, 7, and 14 did not reveal significant differences (*p* > 0.05) compared to the control group treated with saline solution.

**Table 7:** Hematological results for red blood cell profile (including platelets) of mice treated at different doses of ME-Ip or DE-Ip during the acute toxicity study.

	Group	Control	ME-Ip (1000 mg/kg)	DE-Ip (1000 mg/kg)
Parameter				
Hematocrit (L/L)		0.470 ± 0.07	0.460 ± 0.01	0.463 ± 0.04
Hemoglobin (g/L)		140.5 ± 18.53	139.5 ± 4.50	145.67 ± 8.66
Erythrocytes (×10^12^/L)		9.1 ± 1.40	9.0 ± 0.30	9.3 ± 0.53
MCV (fL)		51.5 ± 0.50	51.0 ± 1.00	49.3 ± 1.15
MCHC (g/L)		299.5 ± 5.50	303.0 ± 3.00	314.3 ± 7.50
Reticulocytes (%)		–	–	–
Platelets (×10^9^/L)		335.50 ± 135.50	412.00 ± 118.30	440.00 ± 197.17
Total solids (g/L)		66.00 ± 2.00	68.00 ± 3.46	69.33 ± 1.15

ME-Ip: Methanolic extract of aerial parts of *I. purpurea*. DE-Ip: Dichloromethane extract of aerial parts of *I. purpurea.* Values expressed as mean ± standard deviation, (*n* = 3 per group). No significant differences (*p* > 0.05) were found compared to control animals. MCV: Mean corpuscular volume. MCHC: Mean corpuscular hemoglobin concentration. The control group was treated with saline solution.

**Table 8:** Hematological results for the white blood cell differential profiles of mice treated at different doses of ME-Ip or DE-Ip during the acute toxicity study.

	Group	Control	ME-Ip (1000 mg/kg)	DE-Ip (1000 mg/kg)
Parameter				
Leukocytes (×10^9^/L)		6.63 ± 2.87	10.53 ± 0.83	9.50 ± 0.33
Neutrophils (×10^9^/L)		2.16 ± 0.58	2.20 ± 0.31	2.57 ± 1.15
Band neutrophils (×10^9^/L)		-	-	-
Myelocytes (×10^9^/L)		-	-	-
Lymphocytes (×10^9^/L)		3.68 ± 1.92	8.87 ± 1.30 *	6.17 ± 1.10
Monocytes (×10^9^/L)		0.41 ± 0.19	0.35 ± 0.05	0.27 ± 0.15
Eosinophils (×10^9^/L)		0.05 ± 0.05	0.05 ± 0.05	0.20 ± 0.20
Basophils (×10^9^/L)		-	-	-

ME-Ip: Methanolic extract of aerial parts of *I. purpurea*. DE-Ip: Dichloromethane extract of aerial parts of *I. purpurea*. The control group was treated with saline solution. Values expressed as mean ± standard deviation, (*n* = 3 per group). * Significant differences (*p* < 0.05) compared to the control group.

**Table 9:** Biochemical profiles of mice treated at different doses of ME-Ip or DE-Ip during the acute toxicity study.

	Group	Control	ME-Ip (1000 mg/kg)	DE-Ip (1000 mg/kg)
Parameter				
Urea (mmol/L)		11.24 ± 2.33	10.72 ± 0.05	11.78 ± 2.34
Creatinine (μmol/L)		34.33 ± 5.13	31.00 ± 7.81	37.00 ± 11.36
ALT (U/L)		428.25 ± 97.23	449.0 ± 97.58	491.25 ± 12.37
AST (U/L)		491.75 ± 9.57	476.5 ± 85.61	438.75 ± 52.74
ALP (U/L)		66.5 ± 20.51	64.0 ± 2.00	96.5 ± 21.76
Cholesterol (mmol/L)		2.94 ± 0.61	2.87 ± 0.19	2.75 ± 0.22
Total protein (g/L)		59.0 ± 3.00	59.0 ± 7.21	59.0 ± 2.65

ME-Ip: Methanolic extract of aerial parts of *I. purpurea.* DE-Ip: Dichloromethane extract of aerial parts of *I. purpurea.* ALT: Alanine Transaminase. AST: Aspartate Aminotransferase. ALP: ALP Alkaline Phosphatase. The control group was treated with saline solution. (*n* = 3 per group). Blood biochemistry values of mice in the treatment groups did not reveal significant differences (*p* > 0.05) compared to control animals.

**Table 10:** Histopathology of kidney and liver of mice treated at different doses of ME-Ip and DE-Ip during acute toxicity study.

Group	Kidney	Liver	Histological Sectionof Kidneys and Liver
Negative Control	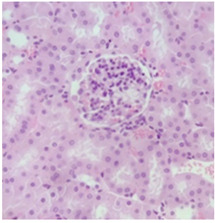	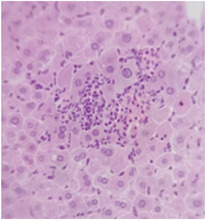	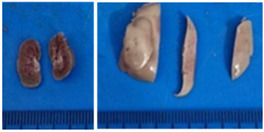
ME-Ip 1000 mg/Kg	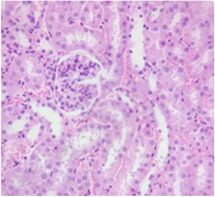	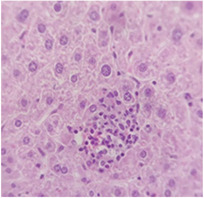	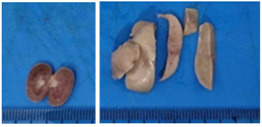
DE-Ip 1000 mg/Kg	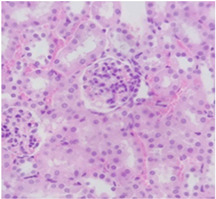	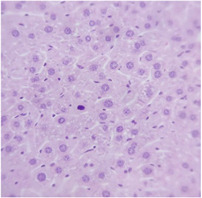	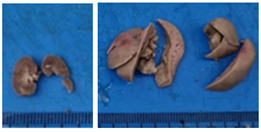
ME-Ip 5000 mg/Kg	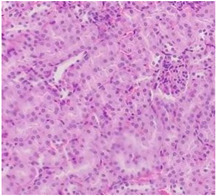	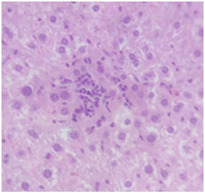	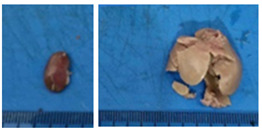
DE-Ip 5000 mg/Kg	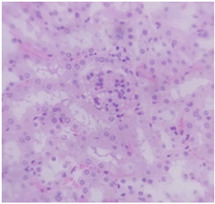	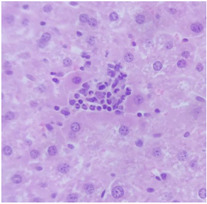	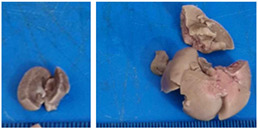

**Table 11:** Phytochemical, pharmacological, and toxicological evaluation of *Ipomoea purpurea* extracts.

Category	Details
Plant Studied	*Ipomoea purpurea* (L.) Roth (aerial parts)
Traditional Uses	Diuretic, purgative, and topical anti-inflammatory and soothing agent in Mexican traditional medicine
Extracts Evaluated	Methanol (MeOH) and Dichloromethane (DCM) extracts
Phytochemical Analysis	- Methanol extract of *I. purpurea* (ME-Ip): UPLC-QTOF-MS - Dichloromethane extract of *I. purpurea* (DE-Ip): GC-MS - First report of glycosylated flavonoids in *Ipomoea* genus - Compounds with potential anti-inflammatory activity identified
Pharmacological Assays	- Vasodilatory activity: ex vivo model - Spasmodic activity: ex vivo model
Toxicological Assessment	- Acute toxicity evaluated in vivo - Neither extract showed acute toxicity
Key Findings	- Glycosylated flavonoids may contribute to biological activity - Extracts exhibit vasodilatory, spasmodic, and purgative properties - Support for traditional uses in cardiovascular and GI disorders
Conclusions	- Findings support the ethnomedical use of *I. purpurea* - ME-Ip and DE-Ip extracts exhibited a biphasic effect (both contractile and spasmolytic) on the spontaneous contractions of isolated rat ileum - Low levels of acute toxicity suggest safety - Potential for phytopharmaceutical development as adjuvants in cardiovascular and gastrointestinal therapies - Further studies are needed to evaluate subacute and chronic toxicity, and to explore additional pharmacological effects of *Ipomoea purpurea*. - Bioactivity-guided fractionation and mechanistic studies are also recommended to identify active compounds and elucidate their vasodilatory and spasmolytic mechanisms.

All tissue samples were stained with hematoxylin and eosin (H/E) and examined using a photonic microscope. A 40× objective with a 10× eyepiece was used, giving a total magnification of 400× (40 × 10). Images were taken using TCapture (version 3.9.0.605), a software used for image acquisition, handling, and digital processing. The sizes of the organs were determined using a millimeter ruler placed next to the tissue; each visible line represents 1 mm. The tissues each comprise a sample from a single animal for a concentration of 1000 mg/kg of either ME-Ip (methanolic extract of aerial parts of *I. purpurea*) or DE-Ip (dichloromethane extract of aerial parts of *I. purpurea*), *n* = 3 per group. The final tissues are samples from single animals for a concentration of 5000 mg/kg of ME-Ip or DE-Ip (*n* = 1 per group, according to the Lorke method).

## Data Availability

The data presented in this study are available on request from the corresponding author. The data are not publicly available due to privacy and ethical restrictions.

## Supplementary Materials

The following supporting information can be downloaded at: https://www.mdpi.com/article/10.3390/ph18081134/s1, Table S1. Fragmentation Pattern Analysis of Proposed Compounds by UPLC-QTOF-MS.

## Abbreviations

The following abbreviations are used in this manuscript:

AH	Arterial hypertension
GDs	Gastrointestinal diseases
NCD’s	Non-communicable diseases
ME-Ip	Methanolic extract of Ipomoea purpurea
DE-Ip	Dichloromethane extract of Ipomoea purpurea
AOC	Antioxidant capacity
DPPH	1,1-diphenyl-2-picrylhydrazyl
FRAP	Ferric reducing antioxidant power
ALT	Alanine aminotransferase
ACE	Angiotensin-converting enzyme
AST	Aspartate aminotransferase
Ach	Acetylcholine
GAE	Gallic acid equivalents
CAE	Catechin equivalents
NF-κB	Nuclear factor kappa B
COX-2	Cyclooxygenase-2
ALP	Alkaline phosphatase
UPLC-QTOF-MS	Ultra-performance liquid chromatography coupled with quadrupole time-of-flight mass spectrometry
GC-MS	Gas Chromatography–Mass Spectrometry
Papv	Papaverine
EC50	Half maximal effective concentration
Emax	Maximum effect

## References

[1] Barquera S., Rivera J. (2020). Obesity in Mexico: Rapid epidemiological transition and food industry interference in health policies. Lancet Diabetes Endocrinol..

[2] Jimenez M.P., Barrientos G.T., Soria-Contreras D.C., Magid H.S., Kaufman S.J. (2022). Increasing representation of epidemiologists from around the world in the society for epidemiologic research: The case of Mexico. Am. J. Epidemiol..

[3] Barquera S., Hernández-Barrera L., Trejo B., Shamah T., Campos-Nonato I., Rivera-Dommarco J. (2020). Obesidad en México, prevalencia y tendencias en adultos. Ensanut 2018-19. Salud Publica Mex..

[4] Pavía A.A., Alcocer M.A., Ruiz E.D., Mayorga J.L., Mehta R., Díaz F.A., Aldrete J.A., López N., Cruz I., Chávez A. (2022). Guía de práctica clínica mexicana para el diagnóstico y tratamiento de las dislipidemias y enfermedad cardiovascular aterosclerótica. Arch. Cardiol. Mex..

[5] Secretaría de Salud en México, más de 30 Millones de Personas Padecen Hipertensión Arterial: Secretaría de Salud. https://www.gob.mx/salud/articulos/en-mexico-mas-de-30-millones-de-personas-padecen-hipertension-arterial-secretaria-de-salud.

[6] Gopar R., Ezquerra A., Chávez N.L., Manzur D., Raymundo G.I. (2021). ¿Cómo tratar la hipertensión arterial sistémica? Estrategias de Tratamiento Actuales. Arch. Cardiol. Mex..

[7] Carey R.M., Moran A.E., Whelton P.K. (2022). Treatment of hypertension: A review. J. Am. Med. Assoc..

[8] Lynch S.S., Adherencia al Tratamiento Farmacológico Manual MSD Versión para Público General. https://www.msdmanuals.com/es/hogar/f%C3%A1rmacos-o-sustancias/factores-que-influyen-en-la-respuesta-del-organismo-a-los-f%C3%A1rmacos/adherencia-al-tratamiento-farmacol%C3%B3gico.

[9] Olaiz G.A., Gómez E.G., Juárez A., Anda F.J., Morales J.E., Carrasco O. (2022). Panorama histórico de la enfermedad diarreica aguda en México y el futuro de su prevención. Salud Publica Mex..

[10] Gotfried J. Generalidades Sobre los síntomas Gastrointestinales. Manual MSD Versión para Profesionales. https://www.msdmanuals.com/es/professional/trastornos-gastrointestinales/s%C3%ADntomas-de-los-trastornos-gastrointestinales/generalidades-sobre-los-s%C3%ADntomas-gastrointestinales.

[11] Hani A. (2014). Antiespasmódicos. Acta Gastroenterol. Latinoam..

[12] Homedes N. (2020). Antiespasmódicos durante el embarazo, en resumen. Prescrire.

[13] Hicks G.A., Taylor J.F., Triggle D.J. (2007). Irritable Bowel Syndrome. Comprehensive Medicinal Chemistry II.

[14] Guzik T.J., Nosalski R., Maffia P., Drummond G.R. (2024). Immune and inflammatory mechanisms in hypertension. Nat. Rev. Cardiol..

[15] Oz H.S. (2017). Nutrients, Infectious and Inflammatory Diseases. Nutrients.

[16] SEMARNAT Plantas Medicinales de México. https://www.gob.mx/semarnat/articulos/plantas-medicinales-de-mexico.

[17] World Health Organization Traditional Medicine. https://www.who.int/es/news-room/questions-and-answers/item/traditional-medicine.

[18] Comisión Nacional de Áreas Naturales Protegidas (CONANP) México Megadiverso. https://www.gob.mx/conanp/articulos/mexico-megadiverso-173682.

[19] Loraine S., Mendoza J.A. (2010). Las plantas medicinales en la lucha contra el cáncer: Relevancia para México. Rev. Mex. Cienc. Farm..

[20] Rzedowski J., Carranza E. (2023). Sinopsis de la familia Convolvulaceae en México. J. Bot. Res. Inst. Texas.

[21] Carranza G.E., Hernández L.P. (2008). Diversidad del género *Ipomoea* L. (Convolvulaceae) en el estado de Michoacán, México. Flora del Bajío y de Regiones Adyacentes.

[22] Ahmad M., Alamgeer N., Ali I., Mohamed A. (2020). *Ipomoea hederacea* Jacq.: A plant with promising antihypertensive and cardio-protective effects. J. Ethnopharmacol..

[23] Jansakul C., Chairuk P., Zia-Ul-Haq M., Imran I. (2018). Effect of *Ipomoea hederacea* Jacq. methanolic extract on blood pressure and relaxation of rat thoracic aorta: Evidence indicated the release of NO and H_2_S. Thai J. Pharm. Sci..

[24] Paula A.B., Hayashi L.S., Freitas J.C. (2003). Anti-inflammatory and antispasmodic activity of *Ipomoea imperati* (Vahl) Griseb (Convolvulaceae). Braz. J. Med. Biol. Res..

[25] Javaid A., Ferdosi M., Manzoor M., Haider I. (2023). Medically important compounds in *Ipomoea carnea* flowers. Pak. J. Weed Sci. Res..

[26] Manhães F., Chaves A., Da Silva M., De Souza Q., Chaves C., Antunes F., Rodrigues R. (2020). Pharmacognosy, phytochemical analysis, and hypotensive activity of *Ipomoea pes-caprae* on blood pressure of normotensive rats. Rodriguésia.

[27] Hamsa T.P., Kuttan G. (2010). Evaluation of the anti-inflammatory and anti-tumor effect of *Ipomoea obscura* (L.) and its mode of action through the inhibition of pro-inflammatory cytokines, nitric oxide and COX-2. Inflammation.

[28] Cai C., Chen Y., Zhong S., Ji B., Wang J., Bai X., Shi G. (2014). Anti-inflammatory activity of n-butanol extract from *Ipomoea stolonifera* in vivo and in vitro. PLoS ONE.

[29] Monsalvo M., Fortunato R., Wagner M., Ricco R. (2018). Estudio farmacobotánico de *Ipomoea purpurea* (L.) Roth (Convolvulaceae). Dominguezia.

[30] Srivastava D., Rauniyar N. (2020). Medicinal Plants of Genus Ipomoea.

[31] Srivastava D. (2017). Medicinal plants of the genus *Ipomoea* found in Uttar Pradesh, India. Res. J. Recent Sci..

[32] León-Rivera I., Herrera-Ruiz M., Estrada-Soto S., Gutiérrez M.C., Martínez-Duncker I., Navarrete-Vázquez G., Rios M.Y., Aguilar B., Castillo-España P., Aguirre-Moreno A. (2011). Sedative, vasorelaxant, and cytotoxic effects of convolvulin from *Ipomoea tyrianthina*. J. Ethnopharmacol..

[33] Perusquía M., Mendoza S., Bye R., Linares E., Mata R. (1995). Vasoactive effects of aqueous extracts from five Mexican medicinal plants on isolated rat aorta. J Ethnopharmacol..

[34] Arias-Ortíz H.M., López-Bedoya A., Bernal-Vera M.E., Castaño-Ramirez E. (2011). Caracterización ecológica y fitoquímica de la batatilla *Ipomoea purpurea* L. Roth (Solanales, Convolvulaceae) en el municipio de Manizales. Bol. Cient. Cent. Mus. Mus. Hist. Nat..

[35] Cao S., Zhao B., Zou Y., Sun Z., Zhang H., Wei S., Ji M. (2022). P450s mediated enhanced herbicide metabolism involved in the thifensulfuron-methyl resistance in *Ipomoea purpurea* L.. Biochem. Physiol..

[36] Rafiq-Kumar M., Tauseef S.M., Abbasi T., Abbasi S.A. (2015). Control of amphibious weed *Ipomoea carnea* by utilizing it for the extraction of volatile fatty acids as energy precursors. J. Adv. Res..

[37] Sharma A., Bachheti R.K. (2013). A review on *Ipomoea carnea*. Int. J. Pharma Bio Sci..

[38] Abbasi T., Anuradha J., Ganaie S.U., Abbasi S.A. (2015). Gainful utilization of the highly intransigent weed *Ipomoea* in the synthesis of gold nanoparticles. J. King Saud Univ. Sci..

[39] Rout S.K., Kar D.M. (2013). Sedative, anxiolytic and anticonvulsant effects of different extracts from the leaves of *Ipomoea carnea* in experimental animals. Int. J. Drug Dev. Res..

[40] Beheshti F., Shabani A.A., Akbari M.R., Kokhaei P., Vazirian M., Safavi M. (2021). Anticancer activity of *Ipomoea purpurea* leaves extracts in monolayer and three-dimensional cell culture. Evid. Based Complement. Alternat. Med..

[41] Beheshti F.M., Safavi M., Eidgahi M.R., Kokhaei P., Vazirian M., Shabani A.A. (2023). Phytochemical screening and in vitro antioxidant activity of extracts of *Ipomoea purpurea* leaves from Iran. Biologia.

[42] Vázquez-Ruiz Z., Toledo E., Vitelli-Storelli F., Goni L., de la O V., Bes-Rastrollo M., Martínez-González M.Á. (2022). Effect of dietary phenolic compounds on incidence of cardiovascular disease in the SUN Project; 10 years of follow-up. Antioxidants.

[43] Negri S., Pietrolucci F., Andreatta S., Njoku R.C., Ramos C.A.S.N., Crimi M., Commisso M., Guzzo F., Avesani L. (2024). Bioprospecting of *Artemisia* genus: From artemisinin to other potentially bioactive compounds. Sci. Rep..

[44] Cervantes R., Barragán M., Chaquilla G. (2019). Antioxidant evaluation in the sweet potato purple (*Ipomoea batatas* L.) leaf tea. Tecnol. Marcha.

[45] Sulaiman C., Geetha S.P., Indira B. (2014). Identification of phenolic antioxidants in *Ipomoea mauritiana* Jacq. using spectrophotometric and mass spectroscopic studies. Avicenna J. Phytomed..

[46] Meira M., Silva E.P., David J.M., David J.P. (2012). Review of the genus *Ipomoea*: Traditional uses, chemistry and biological activities. Rev. Bras. Farmacogn..

[47] Sousa E.O., Costa J.G. (2012). Genus Lantana: Chemical aspects and biological activities. Rev. Bras. Farmacogn..

[48] Liang D., Li H., Pan Y., Liu Z., Xiang H. (2025). Transcriptome analysis of *Ipomoea cairica* algicidal mechanism against *Phaeocystis globosa*. Front. Mar. Sci..

[49] Wang H., Zhang X., Liu Y., Zhang Y., Wang Y., Peng Y., Ding Y. (2023). Diosmetin-7-O-β-D-glucopyranoside suppresses endothelial-mesenchymal transformation through endoplasmic reticulum stress in cardiac fibrosis. Clin. Exp. Pharmacol. Physiol..

[50] Hordyjewska A., Ostapiuk A., Horecka A., Kurzepa J. (2019). Betulin and betulinic acid: Triterpenoids derivatives with a powerful biological potential. Phytochem. Rev..

[51] Ono M., Ueguchi T., Murata H., Kawasaki T., Miyahara K. (1992). Resin glycosides, XVI: Marubajalapins I–VII, new ether-soluble resin glycosides from *Pharbitis purpurea*. Chem. Pharm. Bull..

[52] Lin W.C., Hsu K.C., You M.F., Lee K.H., Chi C.H., Chen J.Y. (2023). Octanoic acid promotes clearance of antibiotic-tolerant cells and eradicates biofilms of *Staphylococcus aureus* isolated from recurrent bovine mastitis. Biofilm.

[53] Boukhers I., Morel S., Kongolo J., Domingo R., Servent A., Ollier L., Kodja H., Petit T., Poucheret P. (2023). Immunomodulatory and Antioxidant Properties of *Ipomoea batatas* Flour and Extracts Obtained by Green Extraction. Curr. Issues Mol. Biol..

[54] Rosas-Ramírez D.G., Escandón-Rivera S., Lazcano-Pérez F., Arreguín-Espinosa R. (2025). El género *Ipomoea*: Desde la época prehispánica hasta la actualidad. Epistemus.

[55] Okechukwu P.N. (2020). Evaluation of anti-inflammatory, analgesic, antipyretic effect of eicosane, pentadecane, octacosane, and heneicosane. Asian J. Pharm. Clin. Res..

[56] Aparna V., Dileep K.V., Mandal P.K., Karthe P., Sadasivan C., Haridas M. (2012). Anti-inflammatory property of n-hexadecanoic acid: Structural evidence and kinetic assessment. Chem. Biol. Drug Des..

[57] Pongprayoon U., Baeckström P., Jacobsson U., Lindström L., Bohlin L. (1992). Antispasmodic Activity of β-Damascenone and E-Phytol Isolated from *Ipomoea pes-caprae*. Planta Med..

[58] Kenakin T. (2016). A Pharmacology Primer: Techniques for More Effective and Strategic Drug Discovery.

[59] Rang H.P., Dale M.M., Ritter J.M., Flower R.J., Henderson G. (2012). Rang & Dale’s Pharmacology.

[60] Sakurai N., Iizuka T., Nakayama S., Funayama H., Noguchi M., Nagai M. (2003). Vasorelaxant activity of caffeic acid derivatives from *Cichorium intybus* and *Equisetum arvense*. Yakugaku Zasshi.

[61] Astragalus Extracts Against Paraoxon-Induced Endothelial Dysfunction in Rat Aorta. CABI Digital Library. https://www.cabidigitallibrary.org/doi/full/10.5555/20113230155.

[62] Qian L.B., Fu J.Y., Cai X., Xia M.L. (2012). Betulinic acid inhibits superoxide anion-mediated endothelial dysfunction in rat aorta. Indian J. Pharmacol..

[63] Oboh G., Agunloye O.M., Adefegha S.A., Akinyemi A.J., Ademiluyi A.O. (2015). Caffeic and chlorogenic acids inhibit key enzymes linked to type 2 diabetes (in vitro): A comparative study. J. Basic Clin. Physiol. Pharmacol..

[64] Suzuki A., Yamamoto M., Jokura H., Yamamoto S., Fujii A., Tokimitsu I., Saito I. (2007). Ferulic acid restores endothelium-dependent vasodilation in aortas of spontaneously hypertensive rats. Am. J. Hypertens..

[65] Arctigenin’s Calcium Antagonist Action on Smooth Muscle BVS Biblioteca Virtual en Salud. https://pesquisa.bvsalud.org/portal/resource/pt/wpr-412222.

[66] Javaid A., Shafique S., Shafique S. (2010). Herbicidal effects of extracts and residue incorporation of Datura metel against parthenium weed. Nat. Prod. Res..

[67] García M. (2018). Evaluación del Efecto Citotóxico de Extractos de *Tagetes lucida* Sobre Líneas Celulares Tumorales. Master’s Thesis.

[68] Pongprayoon U., Bohlin L., Soonthornsaratune P., Wasuwat S. (1991). Anti-inflammatory activity of *Ipomoea pes-caprae* (L.) R. Br. Phytoter. Res..

[69] Jiang F., Li C.G., Rand M.J. (2000). Mechanisms of nitric oxide-independent relaxations induced by carbachol and acetylcholine in rat isolated renal arteries. Br. J. Pharmacol..

[70] Wang S., Zeng J., Yang B., Zhong Y.M. (2014). Bioavailability of caffeic acid in rats and its absorption properties in the Caco-2 cell model. Pharm. Biol..

[71] Godugu C., Patel A.R., Doddapaneni R., Somagoni J., Singh M. (2014). Approaches to improve the oral bioavailability and effects of novel anticancer drugs berberine and betulinic acid. PLoS ONE.

[72] Wang H.M., Şoica C.M., Wenz G.A. (2012). Comparison investigation on the solubilization of betulin and betulinic acid in cyclodextrin derivatives. Nat. Prod. Commun..

[73] Losada-Barreiro S., Celik S., Sezgin-Bayindir Z., Bravo-Fernández S., Bravo-Díaz C. (2024). Carrier systems for advanced drug delivery: Improving drug solubility/bioavailability and administration routes. Pharmaceutics.

[74] de Alencar Silva A., Pereira-de-Morais L., Rodrigues da Silva R.E., de Menezes Dantas D., Brito Milfont C.G., Gomes M.F., Araújo I.M., Kerntopf M.R., de Menezes I.R.A., Barbosa R. (2020). Pharmacological screening of the phenolic compound caffeic acid using rat aorta, uterus and ileum smooth muscle. Chem. Biol. Interact..

[75] Duangjai A., Rawangkan A., Yosboonruang A., Ontawong A., Saokaew S., Goh B.H., Suganuma M., Phisalprapa P. (2024). Antispasmodic activity of light-roasted coffee extract and its potential use in gastrointestinal motility disorders. Foods.

[76] Sadraei H., Ghanadian M., Asghari G., Sekhavati N. (2018). Antispasmodic activity of apigenin and luteolin, two components of *Dracocephalum kotschyi* extract, on rat ileum contractions. J. Herbmed. Pharmacol..

[77] Koech P.K., Boldizsar I., Dobolyi Á., Varro P. (2022). Effects of dibenzylbutyrolactone lignans arctigenin and trachelogenin on the motility of isolated rat ileum. Toxicol. Rep..

[78] Zhao L., Huang Y., Lu L., Yang W., Huang T., Lin Z., Lin C., Kwan H., Wong H.L.X., Chen Y. (2018). Saturated long-chain fatty acid-producing bacteria contribute to enhanced colonic motility in rats. Microbiome.

[79] Gwynne R.M., Thomas E.A., Goh S.M., Sjövall H., Bornstein J.C. (2004). Segmentation induced by intraluminal fatty acid in isolated guinea-pig duodenum and jejunum. J. Physiol..

[80] Leonhardt V., Leal-Cardoso J.H., Lahlou S., Albuquerque A.A., Porto R.S., Celedônio N.R., Oliveira A.C., Pereira R.F., Silva L.P., Garcia-Teófilo T.M. (2010). Antispasmodic effects of essential oil of *Pterodon polygalaeflorus* and its main constituent β-caryophyllene on rat isolated ileum. Fundam. Clin. Pharmacol..

[81] Wu J., Zhang X., Guo L., Sheng Z. (2024). Bioactivity-guided isolation of potential antidiarrheal constituents from *Euphorbia hirta* L. and molecular docking evaluation. Front. Vet. Sci..

[82] Todorova V., Ivanova S., Yotov V., Zaytseva E., Ardasheva R., Turiyski V., Prissadova N., Ivanov K. (2024). Phytoecdysteroids: Quantification in Selected Plant Species and Evaluation of Some Effects on Gastric Smooth Muscles. Molecules.

[83] Jodynis-Liebert J., Kujawska M. (2020). Dosis-respuesta bifásica inducida por fitoquímicos: Evidencia experimental. J. Clin. Med..

[84] Zevallos Escobar L.E., Arroyo Acebedo J.L. (2013). Efecto sobre el músculo liso intestinal y toxicidad aguda oral de un extracto de chilca (*Baccharis latifolia*). Crescendo.

[85] Xi X., Wang J., Qin Y., You Y., Huang W., Zhan J. (2022). The Biphasic Effect of Flavonoids on Oxidative Stress and Cell Proliferation in Breast Cancer Cells. Antioxidants.

[86] Albulescu L., Suciu A., Neagu M., Tanase C., Pop S. (2024). Differential Biological Effects of *Trifolium pratense* Extracts—In Vitro Studies on Breast Cancer Models. Antioxidants.

[87] McLendon K., Preuss C.V. (2025). Atropine. StatPearls. https://www.ncbi.nlm.nih.gov/books/NBK470551/.

[88] Wong M.Y.W., Hebbard G., Gibson P.R., Burgell R.E. (2020). Chronic constipation and abdominal pain: Independent or closely interrelated symptoms. J. Gastroenterol. Hepatol..

[89] Mehmood M.H., Munir S., Khalid U.A., Asrar M., Gilani A.H. (2015). Antidiarrheal, antisecretory and antispasmodic activities of *Matricaria chamomilla* are mediated predominantly through K^+^-channels activation. BMC Complement. Med. Ther..

[90] Shah A.J., Bhulani N.N., Khan S.H., Rehman N.U., Gilani A.H. (2010). Calcium-channel-blocking activity of *Mentha longifolia* L. explains its medicinal use in diarrhea and gut spasm. Phytother. Res..

[91] Corsetti M., Forestier S., Jiménez M. (2023). Hyoscine butylbromide mode of action on bowel motility: From pharmacology to clinical practice. Neurogastroenterol. Motil..

[92] Pomeroy A.R., Rand M.J. (1969). Anticholinergic effects and passage through the intestinal wall of N-butylhyoscine bromide. J. Pharm. Pharmacol..

[93] Mearin F., Ciriza C., Mínguez M., Rey E., Mascort J.J., Balboa A., Díaz-Rubio M. (2016). Guía de práctica clínica: Síndrome del intestino irritable con estreñimiento y estreñimiento funcional en adultos. Rev. Esp. Enferm. Dig..

[94] Bylund D.B. (2014). Senna. Reference Module in Biomedical Sciences.

[95] Le J., Ji H., Zhou X., Wei X., Chen Y., Fu Y., Ma Y., Han Q., Sun Y., Gao Y. (2021). Pharmacology, Toxicology, and Metabolism of Sennoside A, A Medicinal Plant-Derived Natural Compound. Front. Pharmacol..

[96] Hara H., Ise Y., Morimoto N., Shimazawa M., Ichihashi K., Ohyama M., Iinuma M. (2008). Laxative effect of agarwood leaves and its mechanism. Biosci. Biotechnol. Biochem..

[97] García K.R., Remes J.M. (2021). Constipación crónica. Conceptos actuales desde la fisiopatología hasta el tratamiento. Acta Gastroenterol. Latinoam..

[98] Vinardell M.P. (2021). Are there alternatives to animal experimentation?. RBD.

[99] Lorke D. (1983). A new approach to practical acute toxicity testing. Arch. Toxicol..

[100] Burkholder T., Foltz C., Karlsson E., Linton C.G., Smith J.M. (2012). Health Evaluation of Experimental Laboratory Mice. Curr. Protoc. Mouse Biol..

[101] Charles River Laboratories CD-1® Mouse Hematology Data. https://www.criver.com/resources/cd-1-mouse-clinical-pathology-data.

[102] Liu F., Liu Y., Peng Q., Wang G., Tan Q., Ou Z., Xu Q., Liu C., Zuo D., Zhao J. (2022). Creatinine accelerates APAP-induced liver damage by increasing oxidative stress through ROS/JNK signaling pathway. Front. Pharmacol..

[103] Bahadar H., Maqbool F., Niaz K., Abdollahi M. (2016). Toxicity of Nanoparticles and an Overview of Current Experimental Models. Iran J. Pharm. Res..

[104] El Hilaly J., Israili Z.H., Lyoussi B. (2004). Acute and chronic toxicological studies of *Ajuga iva* in experimental animals. J. Ethnopharmacol..

[105] Agbaje E.O., Adeneye A.A., Daramola A.O. (2009). Biochemical and toxicological studies of aqueous extract of *Syzygium aromaticum* (L.) Merr. & Perry (Myrtaceae) in rodents. Afr. J. Trad. CAM.

[106] Ukwuani A.N., Abubakar M.G., Hassan S.W., Agaie B.M. (2012). Toxicological studies of hydromethanolic leaves extract of *Grewia crenata*. Int. J. Pharm. Sci. Drug Res..

[107] (2001). Especificaciones Técnicas para la Producción, Cuidado y uso de los Animales de Laboratorio.

[108] (2002). Protección Ambiental–Salud Ambiental–Residuos Peligrosos Biológico-Infecciosos–Clasificación y Especificaciones de Manejo.

[109] Hinojosa Dávalos J., Gutiérrez Lomelí M., Siller López F., Rodríguez Sahagún A., Morales Del Río J.A., Guerrero Medina P.J., Del-Toro-Sánchez C.L. (2012). Screening fitoquímico y capacidad antiinflamatoria de hojas de *Tithonia tubaeformis*. Rev. Cienc. Biol. Salud.

[110] Kashyap S., Rani L. (2021). Phytochemical screening of primary and secondary metabolites in four species of *Ipomoea* (*I. aquatica*, *I. batata*, *I. carnea*, *I. palmata*)-an underutilized ethnomedicinal weeds of Jharkhand. Biospectra.

[111] Muhamad M., Ai Sze W., Zulkifli N.S., Ab-Rahim S. (2023). Qualitative Analysis on the Phytochemical Compounds and Total Phenolic Content of *Cissus hastata* (Semperai) Leaf Extract. Int. J. Plant Biol..

[112] Pérez M., Dominguez-López I., Lamuela-Raventós R.M. (2023). The Chemistry Behind the Folin–Ciocalteu Method for the Estimation of (Poly)phenol Content in Food: Total Phenolic Intake in a Mediterranean Dietary Pattern. J. Agric. Food Chem..

[113] Cantin C.M., Moreno M.A., Gogorcena Y. (2009). Evaluation of the antioxidant capacity, phenolic compounds, and vitamin C content of different peach and nectarine [*Prunus persica* (L.) Batsch] breeding progenies. J. Agric. Food Chem..

[114] Kumari D., Singh D., Meena M., Janmeda P., Siddiqui M.H. (2024). Qualitative, Quantitative, In Vitro Antioxidant Activity and Chemical Profiling of *Leptadenia pyrotechnica* (Forssk.) Decne Using Advanced Analytical Techniques. Antioxidants.

[115] Castro-Ruiz J.E., Rojas-Molina A., Luna-Vázquez F.J., Rivero-Cruz F., Garcia-Gasca T., Ibarra-Alvarado C. (2017). Affinin (Spilanthol), isolated from *Heliopsis longipes*, induces vasodilation via activation of gasotransmitters and prostacyclin signaling pathways. Int. J. Mol. Sci..

[116] Ibarra-Alvarado C., Rojas A., Mendoza S., Bah M., Gutiérrez D.M., Hernández-Sandoval L., Martínez M. (2010). Vasoactive and antioxidant activities of plants used in Mexican traditional medicine for the treatment of cardiovascular diseases. Pharm. Biol..

[117] Luna-Vázquez F.J., Ibarra-Alvarado C., Camacho-Corona M.D.R., Rojas-Molina A., Rojas-Molina J.I., García A., Bah M. (2018). Vasodilator activity of compounds isolated from plants used in Mexican traditional medicine. Molecules.

[118] Ventura-Martínez R., Rodríguez R., González-Trujano M.E., Ángeles-López G.E., Déciga-Campos M., Gómez C. (2017). Spasmogenic and spasmolytic activities of *Agastache mexicana* ssp. mexicana and *A. mexicana* ssp. xolocotziana methanolic extracts on the guinea pig ileum. J. Ethnopharmacol..

[119] Nasu T., Murase H., Shibata H. (1994). Manganese ions induce tonic contraction after relaxation in a high-K^+^ medium in ileal longitudinal smooth muscle of guinea-pig. J. Pharm. Pharmacol..

[120] Chinedu E., Arome D., Ameh F.S. (2013). A new method for determining acute toxicity in animal models. Int. J. Toxicol..

[121] Watafua M., Ejiofor J.I., Musa A., Ahmad M.H. (2022). Toxicological study on methanol root bark extract of *Acacia sieberiana* (Fabaceae) in Wistar rats. bioRxiv.

