# Supplementary material for: Phytochemical Profile, Vasodilatory and Biphasic Effects on Intestinal Motility, and Toxicological Evaluation of the Methanol and Dichloromethane Extracts from the Aerial Parts of Ipomoea purpurea Used in Traditional Mexican Medicine
Source: Pharmaceuticals (Basel). 2025 Jul 30;18(8):1134. doi: 10.3390/ph18081134 (PMC12389191; doi:10.3390/ph18081134)
Supplement: Supplementary file 1 [file pharmaceuticals-18-01134-s001.zip › pharmaceuticals-3731597-supplementary.pdf]

Table S1. Fragmentation Pattern Analysis of Proposed Compounds by UPLC-QTOF-MS

**Proposed Compound:** Caffeic acid 4-O-glucoside
**[M–H]⁻**<br>**(parent ion observed)**<br>341.08774<br>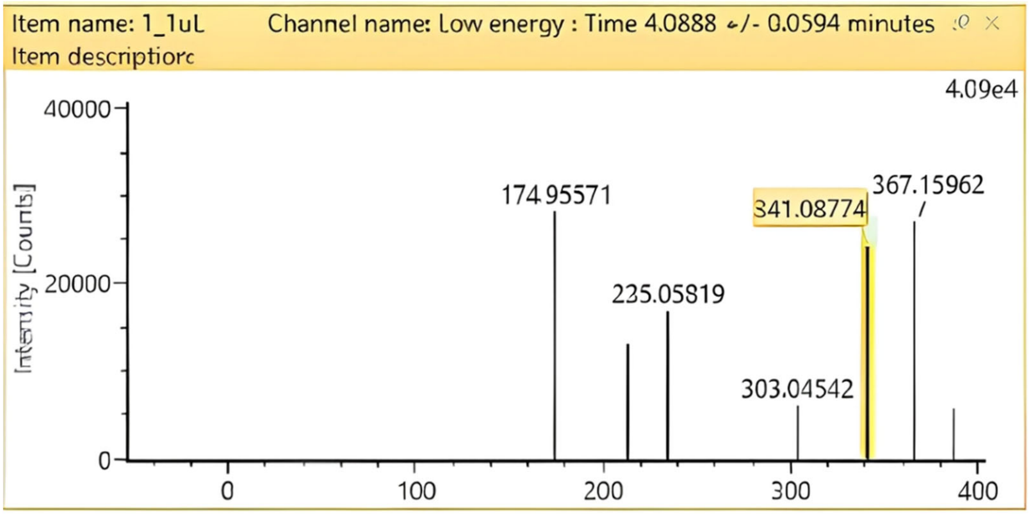
**[M–H]⁻**<br>**(parent ion reported)**<br>341.0873<br>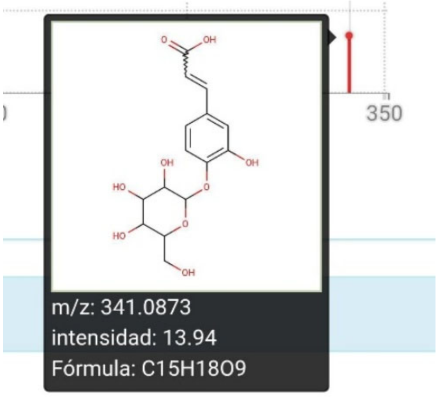<br>https://hmdb.ca/spectra/ms_ms/165427
**[M–H]⁻**

Table S1. Fragmentation Pattern Analysis of Proposed Compounds by UPLC-QTOF-MS

**(fragment 1 observed)**<br><br>297.03008<br><br>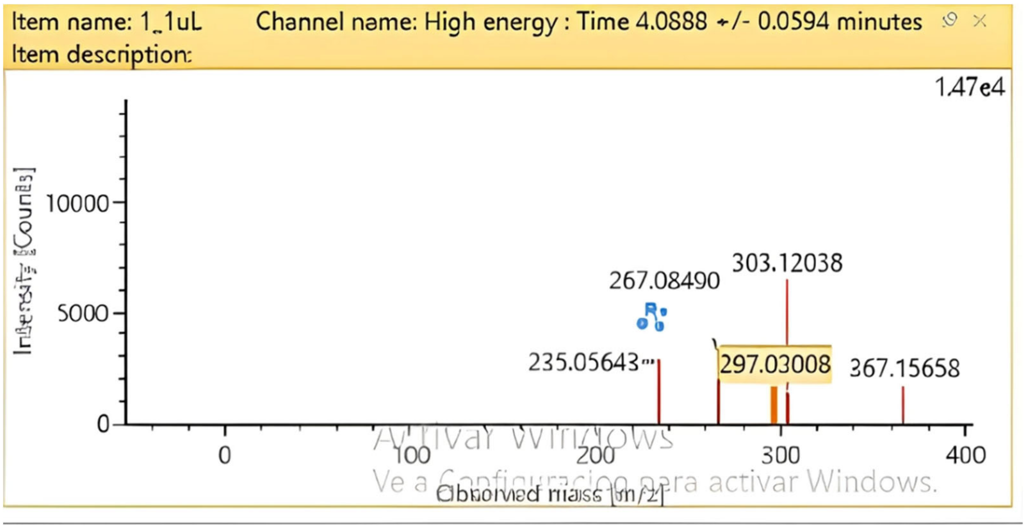
**[M–H]⁻**<br>**(fragment 1 reported)**<br><br>297.098<br><br>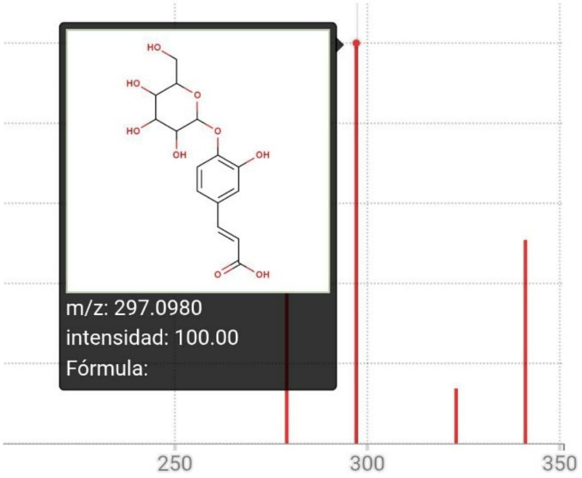<br><br>https://hmdb.ca/spectra/ms_ms/2350603
**[M–H]⁻**<br>**(fragment 2 observed)**<br><br>235.05643

Table S1. Fragmentation Pattern Analysis of Proposed Compounds by UPLC-QTOF-MS

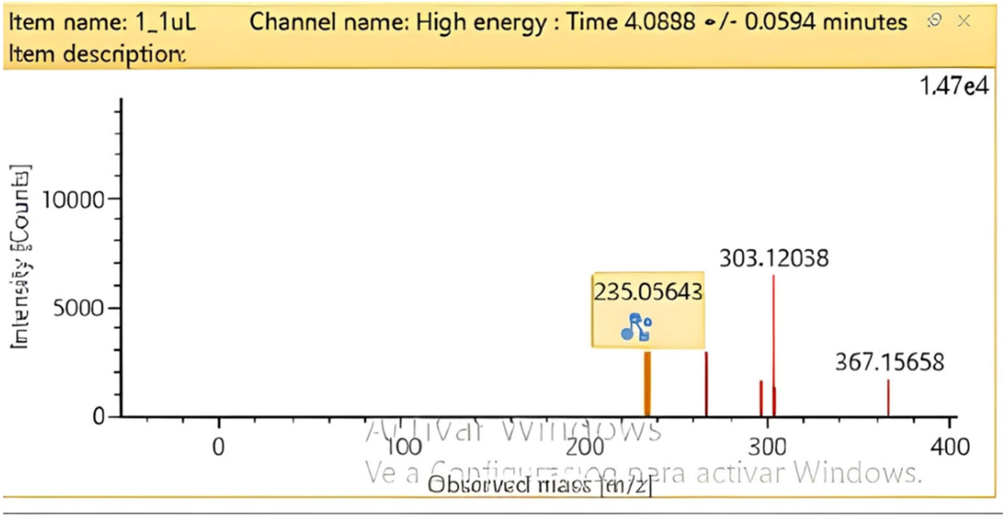
**[M–H]⁻**<br>**(fragment 2 reported)**<br><br>237.0399<br>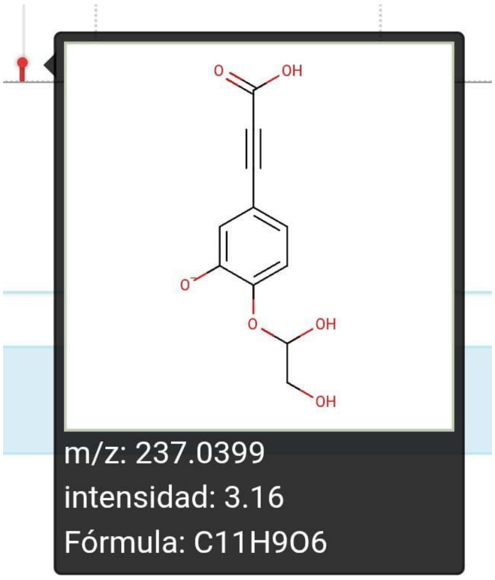<br>https://hmdb.ca/spectra/ms_ms/165427
**Proposed Compound:** Astragalin
**[M–H]⁻**<br><br>**(parent ion observed)**<br><br>447.0933

Table S1. Fragmentation Pattern Analysis of Proposed Compounds by UPLC-QTOF-MS

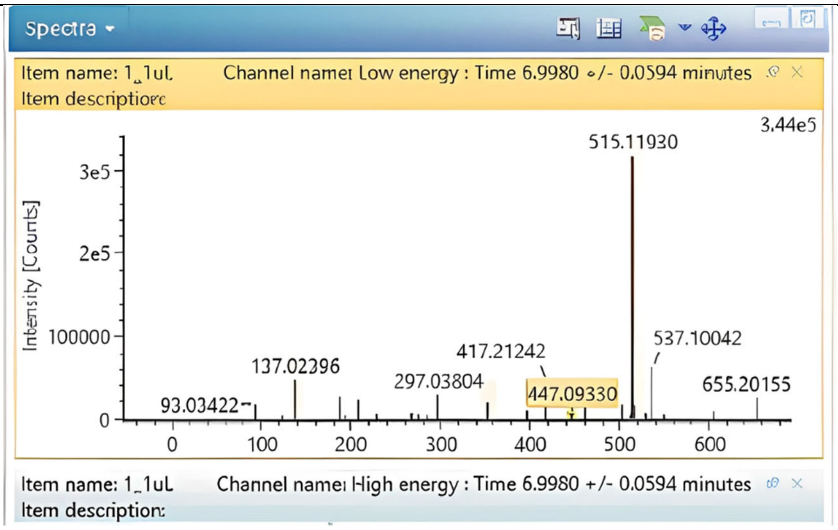

[M–H]⁻

(parent ion reported)

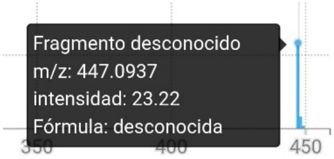

https://hmdb.ca/spectra/ms_ms/373846

[M–H]⁻

(fragment 1 observed)

283.02514

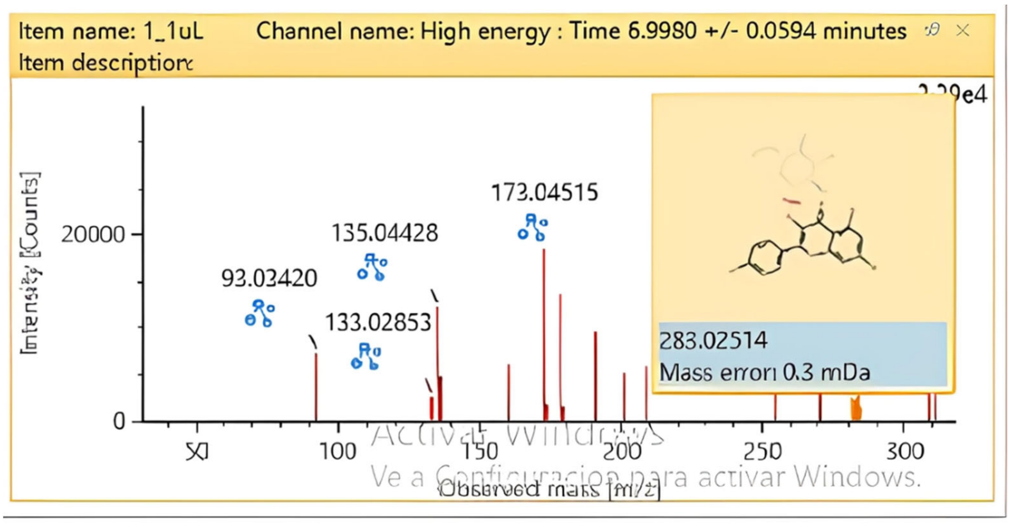

[M–H]⁻

(fragment 1 reported)

283.0605

Table S1. Fragmentation Pattern Analysis of Proposed Compounds by UPLC-QTOF-MS

<table>
<tr><td>
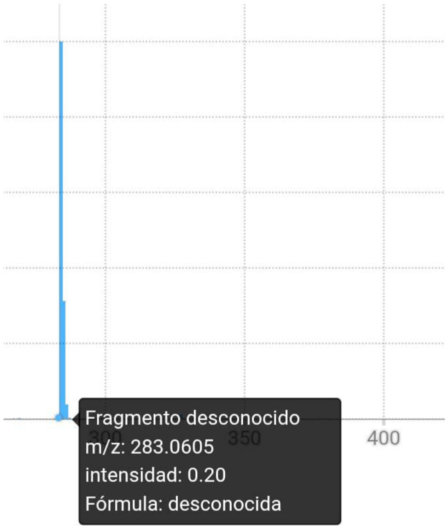

https://hmdb.ca/spectra/ms_ms/285881
</td></tr>
<tr><td>
[M–H]⁻
(fragment 2 observed)

255.02728

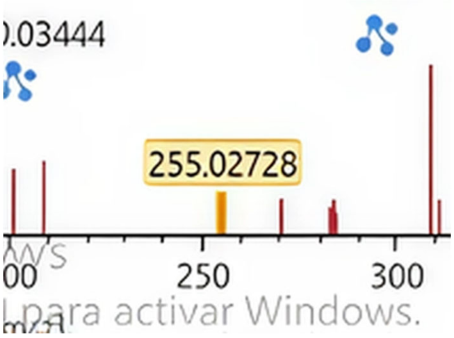

</td></tr>
<tr><td>
[M–H]⁻
(fragment 2 reported)

255.0294
</td></tr>
</table>

Table S1. Fragmentation Pattern Analysis of Proposed Compounds by UPLC-QTOF-MS

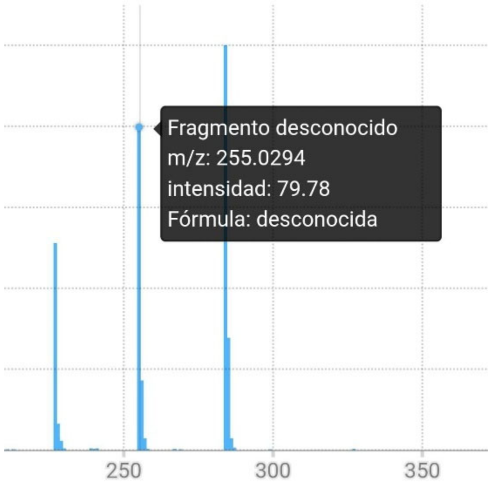

https://hmdb.ca/spectra/ms_ms/285882

[M–H]⁻
(fragment 3 observed)

446.08195

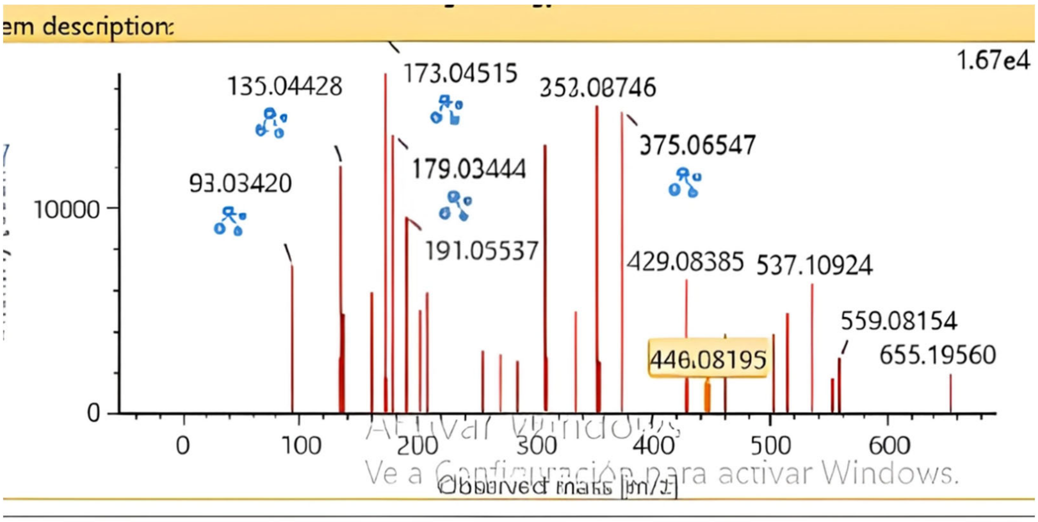

[M–H]⁻
(fragment 3 reported)

447.0937

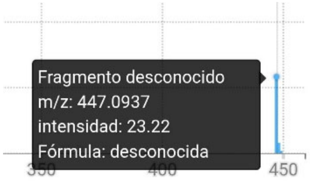

https://hmdb.ca/spectra/ms_ms/373846

Table S1. Fragmentation Pattern Analysis of Proposed Compounds by UPLC-QTOF-MS

**Proposed Compound:** Caffeic acid
**[M–H]⁻**<br>**(parent ion observed)**<br>179.03447<br>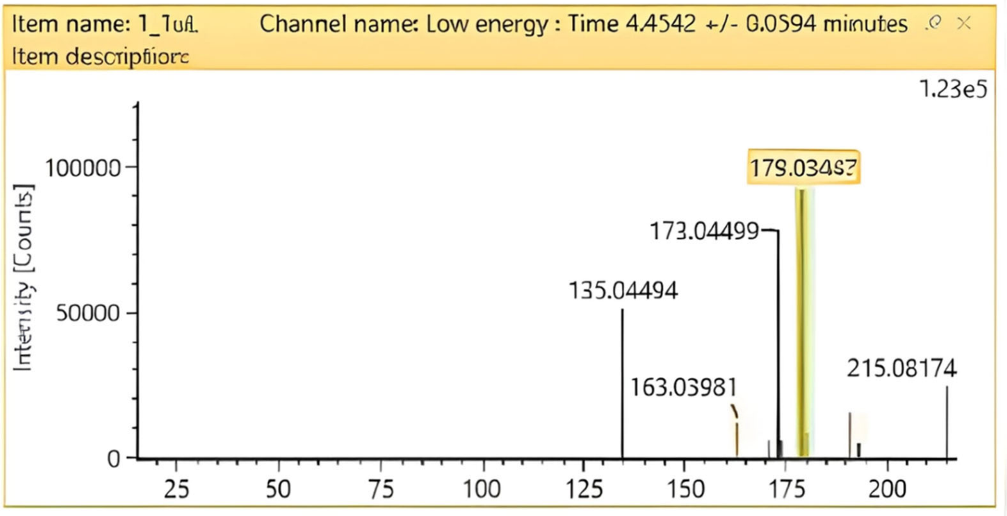
**[M–H]⁻**<br>**(parent ion reported)**<br>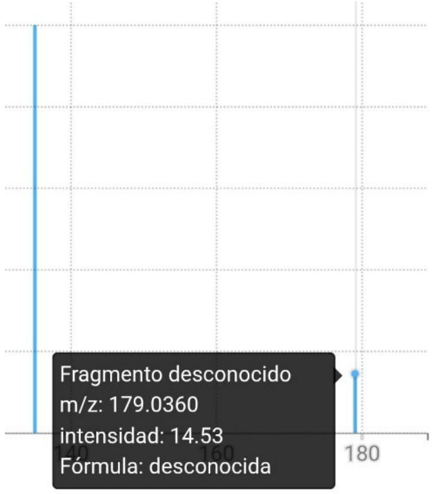<br>https://hmdb.ca/spectra/ms_ms/2256870
**[M–H]⁻**<br>**(fragment 1 observed)**<br>135.04485

Table S1. Fragmentation Pattern Analysis of Proposed Compounds by UPLC-QTOF-MS

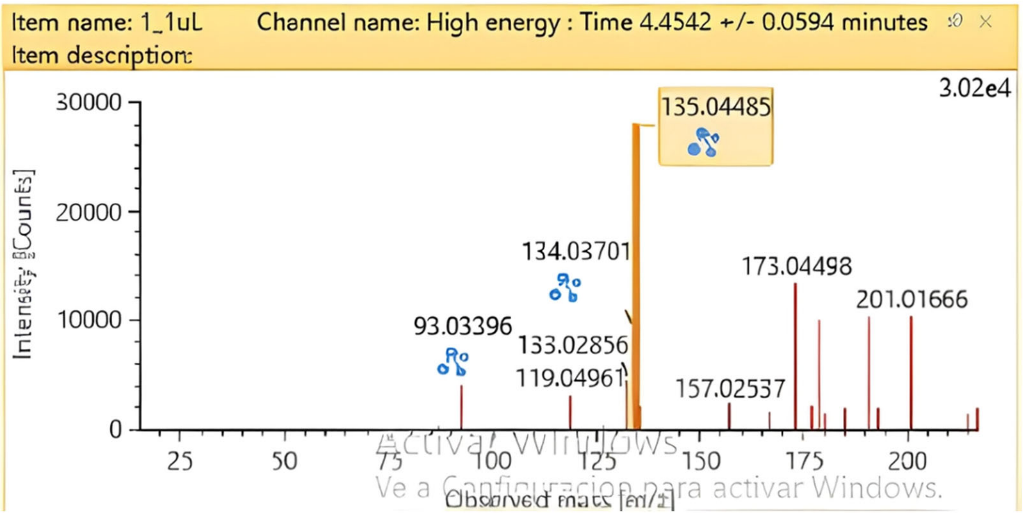

[M–H]⁻
(fragment 1 reported)

135.0458

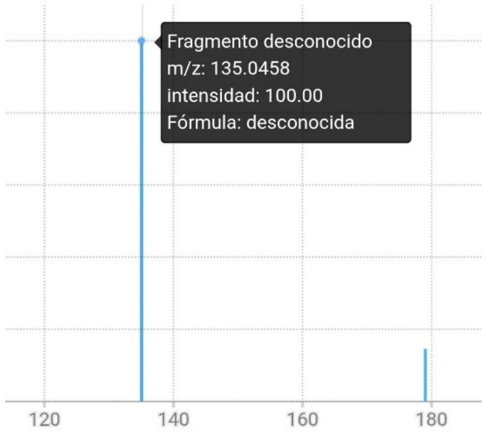

https://hmdb.ca/spectra/ms_ms/2256870

[M–H]⁻
(fragment 2 observed)

133.02856

Table S1. Fragmentation Pattern Analysis of Proposed Compounds by UPLC-QTOF-MS

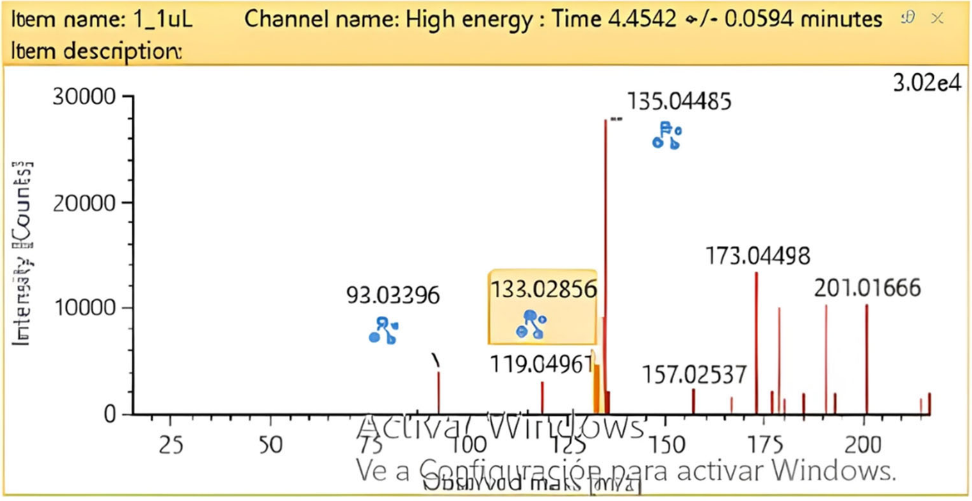
**[M–H]⁻**<br>**(fragment 2 reported)**<br><br>133.0285<br><br>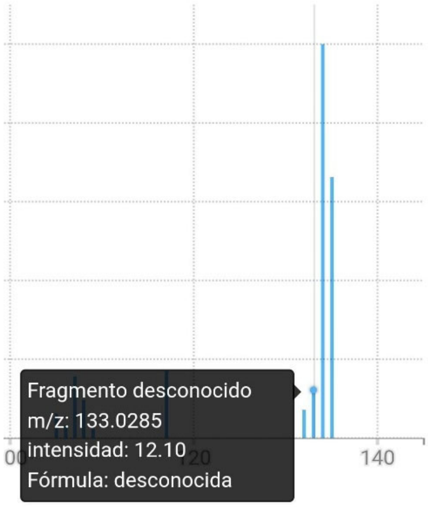<br><br>https://hmdb.ca/spectra/ms_ms/2230277
**[M–H]⁻**<br>**(fragment 3 observed)**<br><br>93.03396

Table S1. Fragmentation Pattern Analysis of Proposed Compounds by UPLC-QTOF-MS

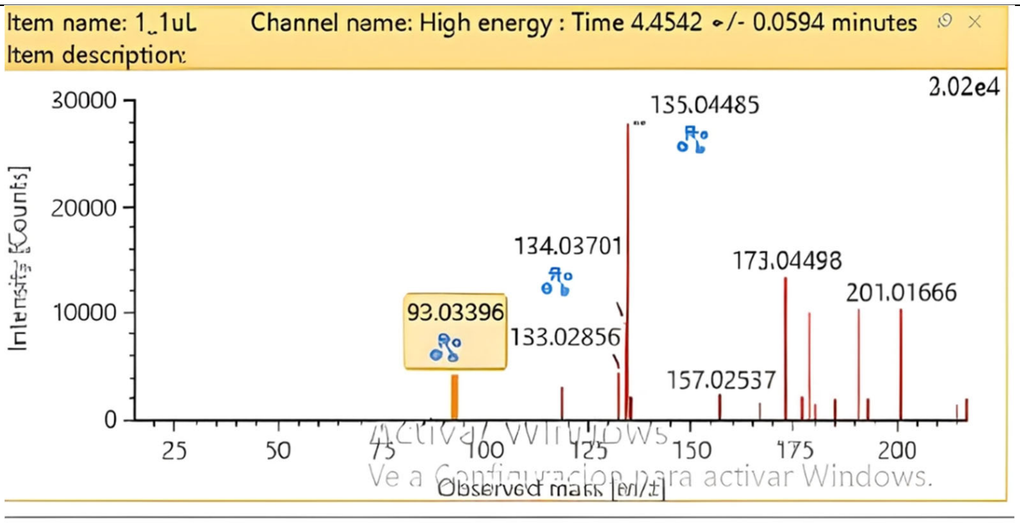

[M–H]⁻
(fragment 3 reported)

93.0349

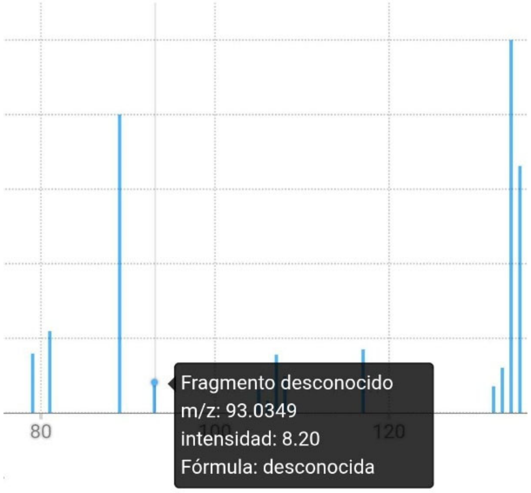

https://hmdb.ca/spectra/ms_ms/2230277

Proposed Compound: Chlorogenic acid

[M–H]⁻

(parent ion observed)

353.06627

Table S1. Fragmentation Pattern Analysis of Proposed Compounds by UPLC-QTOF-MS

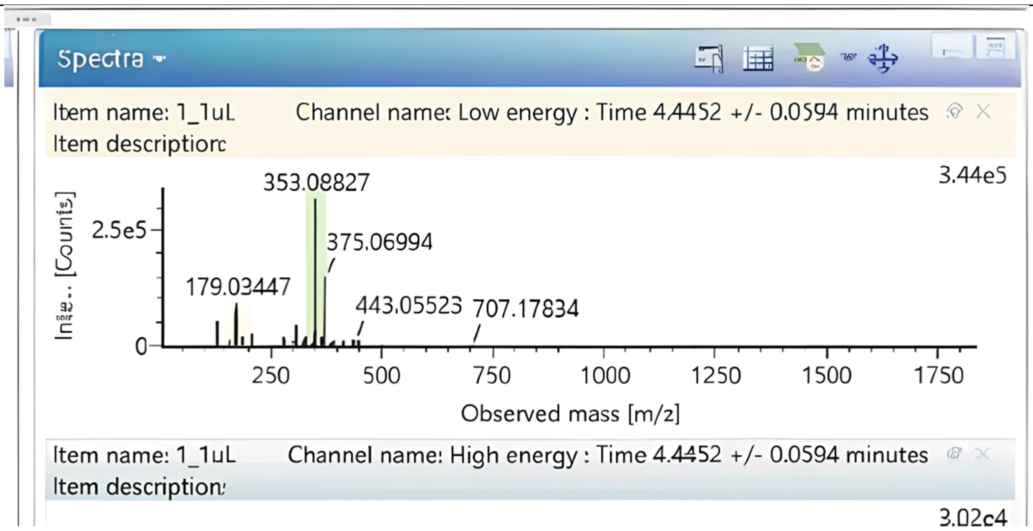

[M–H]⁻
(parent ion reported)

353.0873

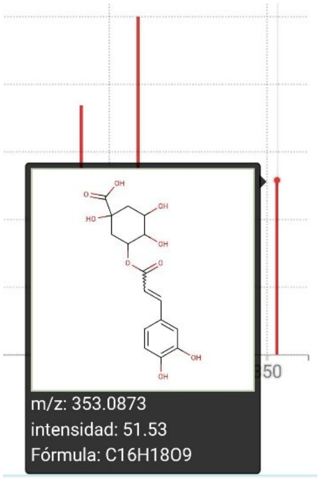

https://hmdb.ca/spectra/ms_ms/278260

[M–H]⁻
(fragment 1 observed)

133.02923

Table S1. Fragmentation Pattern Analysis of Proposed Compounds by UPLC-QTOF-MS

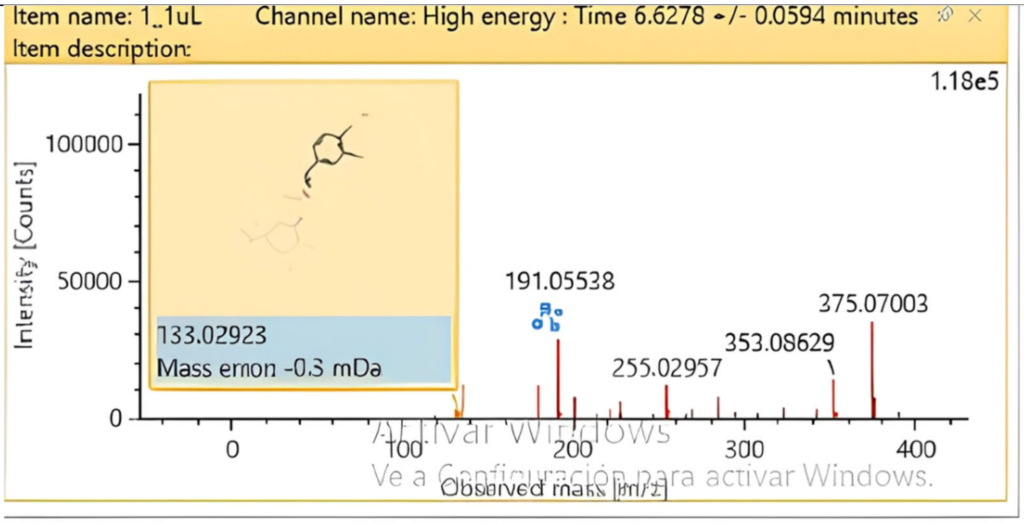
**[M–H]⁻**<br>**(fragment 1 reported)**<br><br>133.0290<br><br>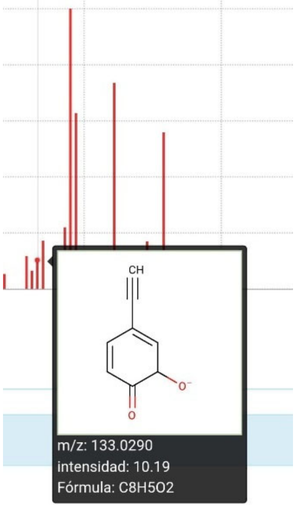<br>https://hmdb.ca/spectra/ms_ms/278261
**[M–H]⁻**<br>**(fragment 2 observed)**<br><br>191.05538

Table S1. Fragmentation Pattern Analysis of Proposed Compounds by UPLC-QTOF-MS

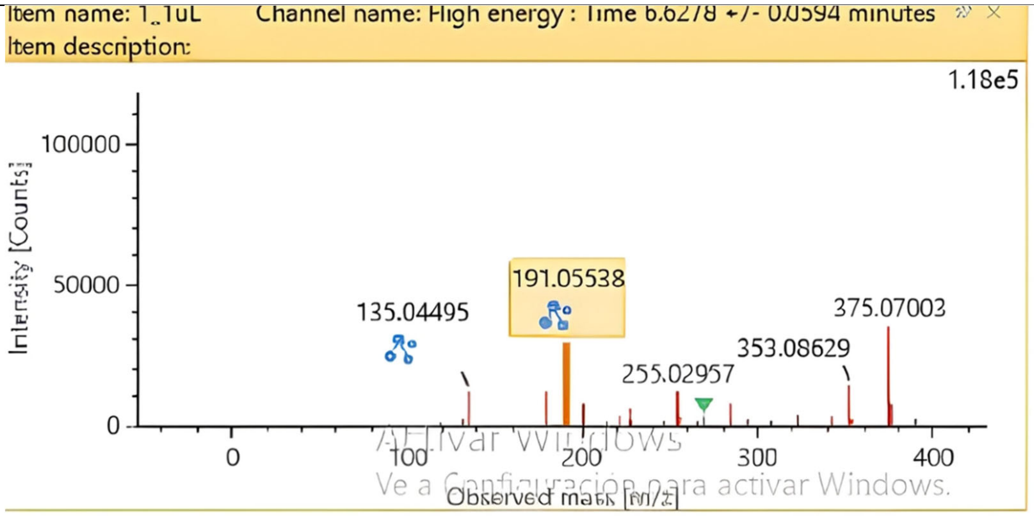
**[M–H]⁻**<br>**(fragment 2 reported)**<br><br>191.0556<br>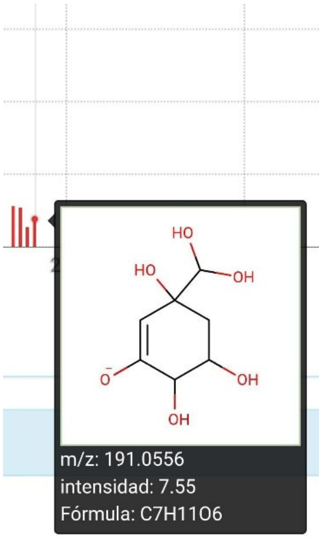<br>https://hmdb.ca/spectra/ms_ms/278261
**[M–H]⁻**<br>**(fragment 3 observed)**<br><br>135.04495

Table S1. Fragmentation Pattern Analysis of Proposed Compounds by UPLC-QTOF-MS

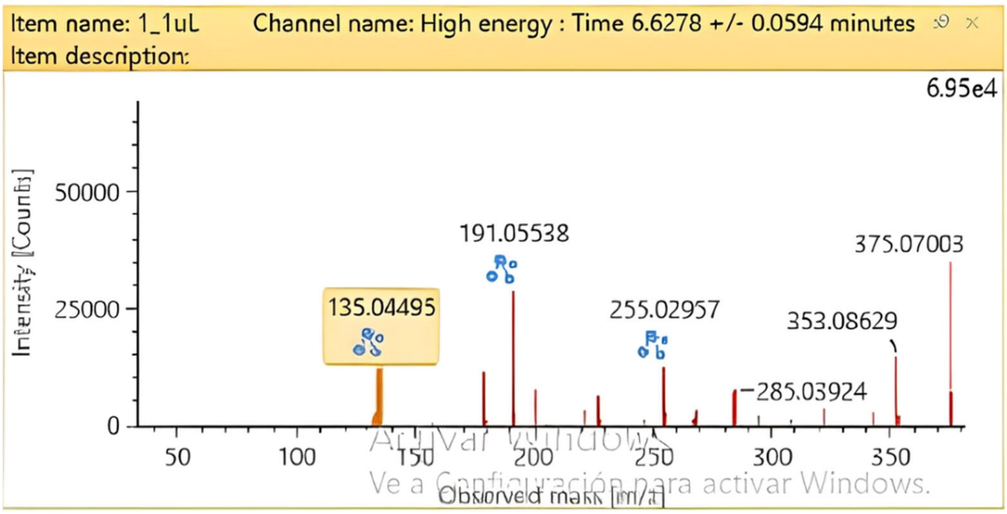

[M–H]⁻
(fragment 3 reported)

135.0446

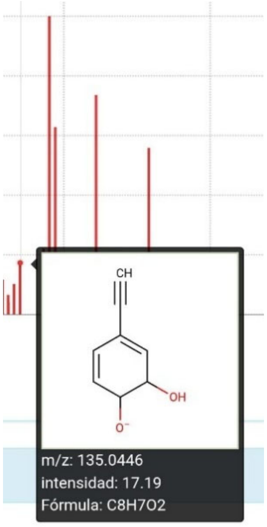

https://hmdb.ca/spectra/ms_ms/278261

Proposed Compound: Ferulic acid

[M–H]⁻

(parent ion observed)

193.05000

Table S1. Fragmentation Pattern Analysis of Proposed Compounds by UPLC-QTOF-MS

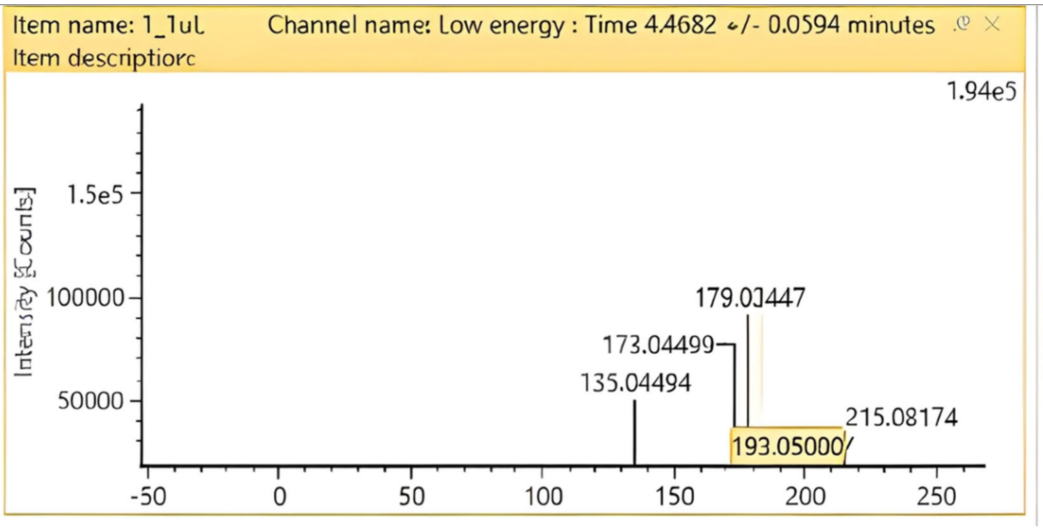
**[M–H]⁻**<br>**(parent ion reported)**<br>193.10<br>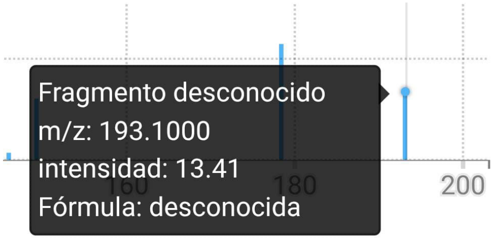<br>https://hmdb.ca/spectra/ms_ms/4947
**[M–H]⁻**<br>**(fragment 1 observed)**<br>177.01853<br>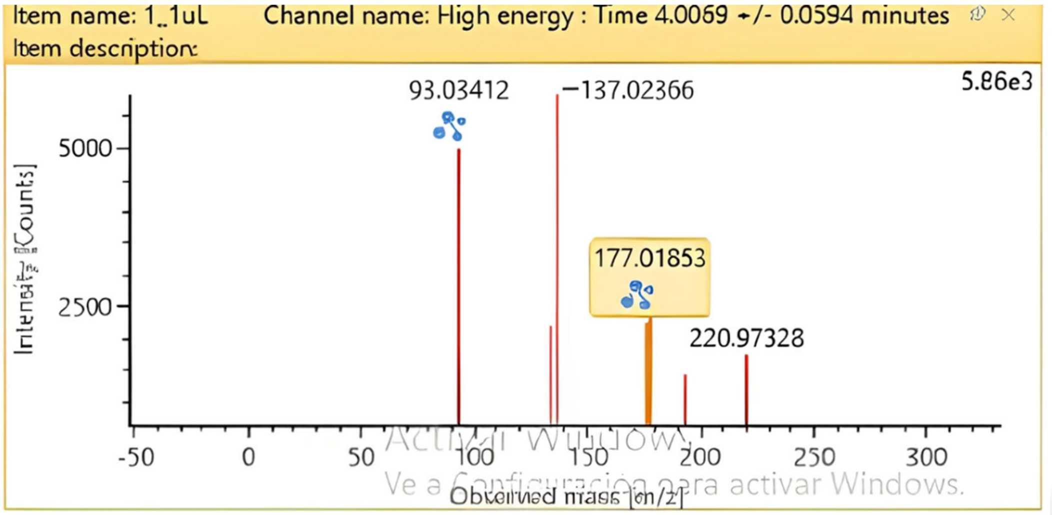
**[M–H]⁻**

Table S1. Fragmentation Pattern Analysis of Proposed Compounds by UPLC-QTOF-MS

**(fragment 1 reported)**<br>177.0188<br>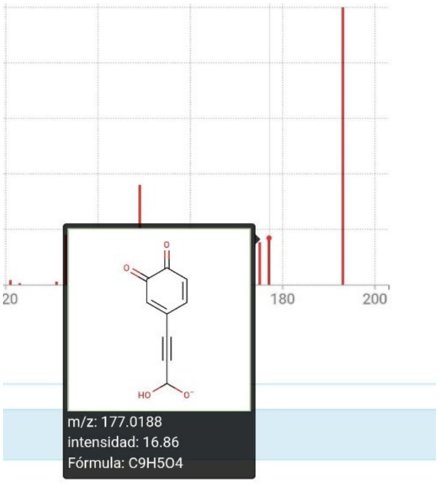<br>https://hmdb.ca/spectra/ms_ms/180289
**[M–H]⁻**<br>**(fragment 2 observed)**<br>93.03412<br>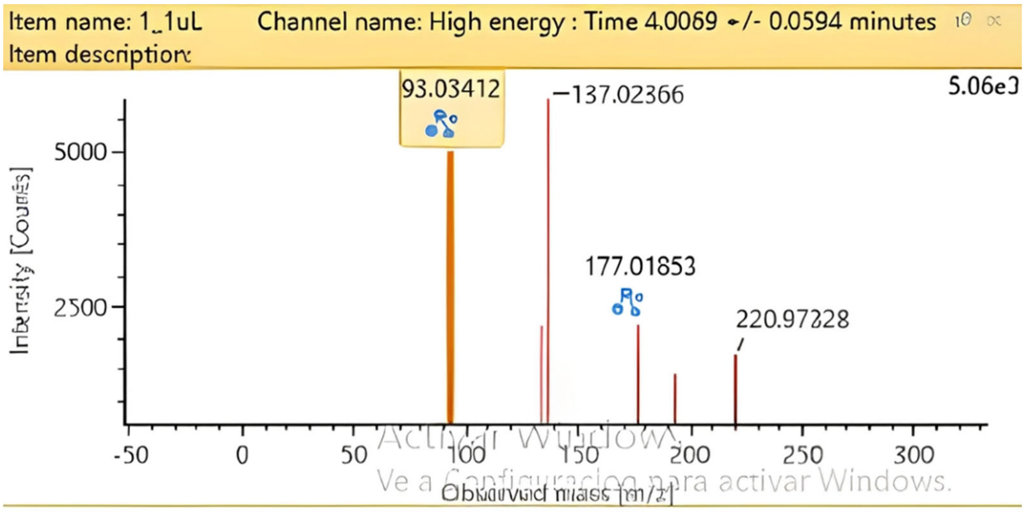
**[M–H]⁻**<br>**(fragment 2 reported)**<br>93.034

Table S1. Fragmentation Pattern Analysis of Proposed Compounds by UPLC-QTOF-MS

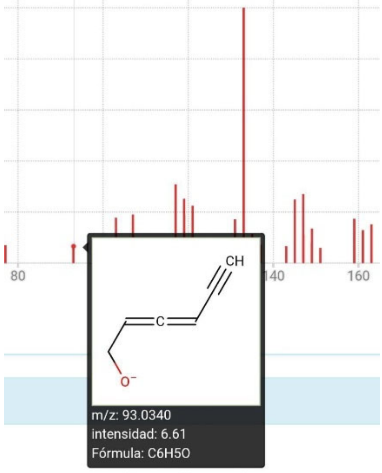<br>https://hmdb.ca/spectra/ms_ms/180290
**[M–H]⁻**<br>**(fragment 3 observed)**<br><br>137.0236<br>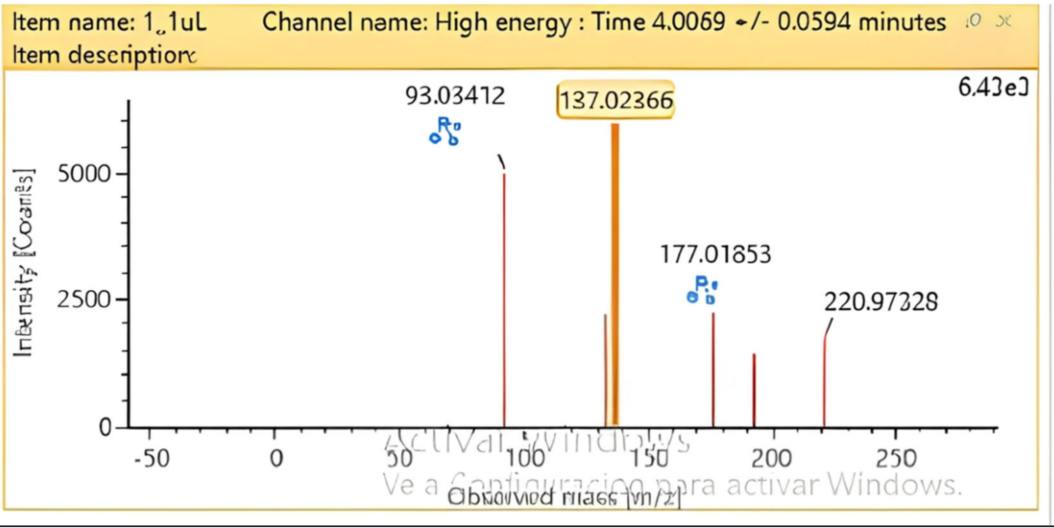
**[M–H]⁻**<br>**(fragment 3 reported)**<br><br>137.0239

Table S1. Fragmentation Pattern Analysis of Proposed Compounds by UPLC-QTOF-MS

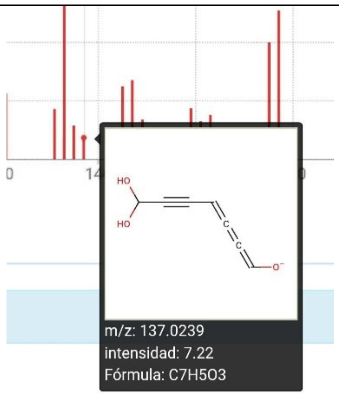

https://hmdb.ca/spectra/ms_ms/180290

Proposed Compound: N-cis-feruloyltyramine

[M–H]⁻

(parent ion observed)

312.12398

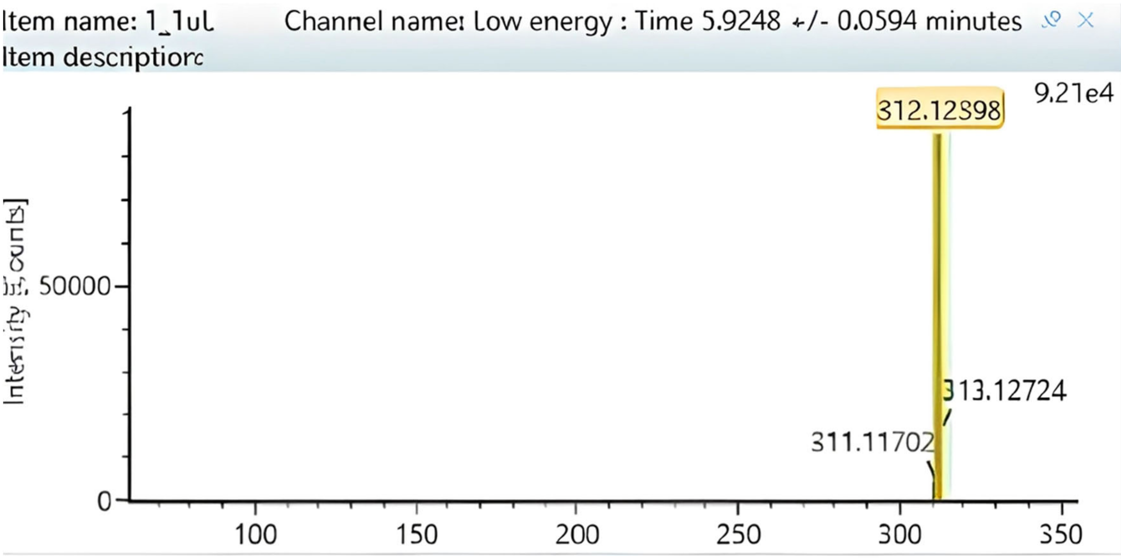

[M–H]⁻

(parent ion reported)

312.1241

Table S1. Fragmentation Pattern Analysis of Proposed Compounds by UPLC-QTOF-MS

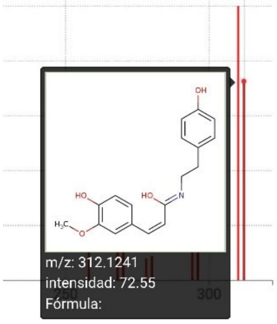

https://hmdb.ca/spectra/ms_ms/2729159

[M–H]⁻
(fragment 1 observed)

135.04415

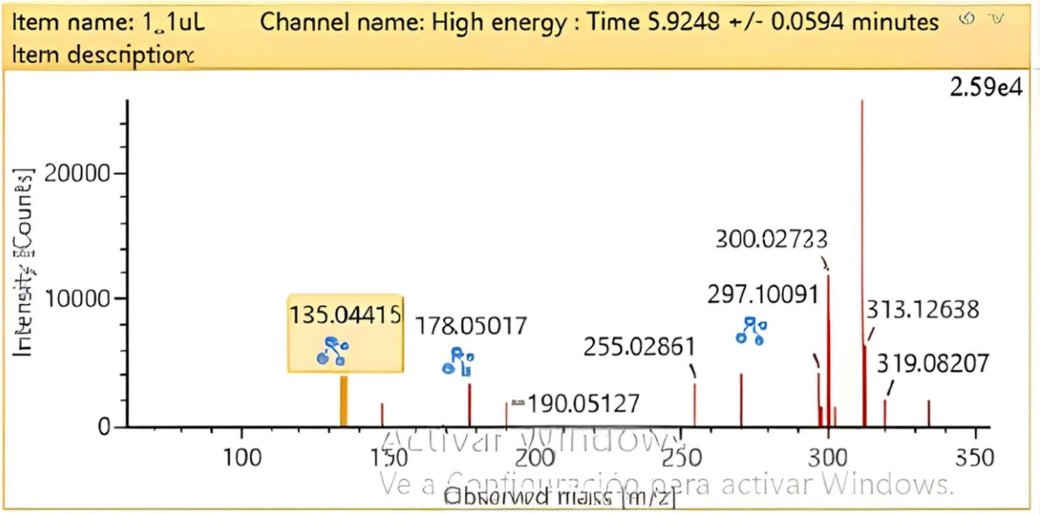

[M–H]⁻
(fragment 1 reported)

135.0446

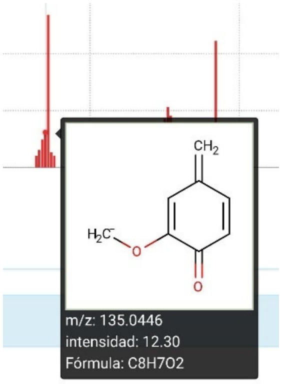

https://hmdb.ca/spectra/ms_ms/147541

Table S1. Fragmentation Pattern Analysis of Proposed Compounds by UPLC-QTOF-MS

**[M–H]⁻**<br>**(fragment 2 observed)**<br><br>178.05017<br>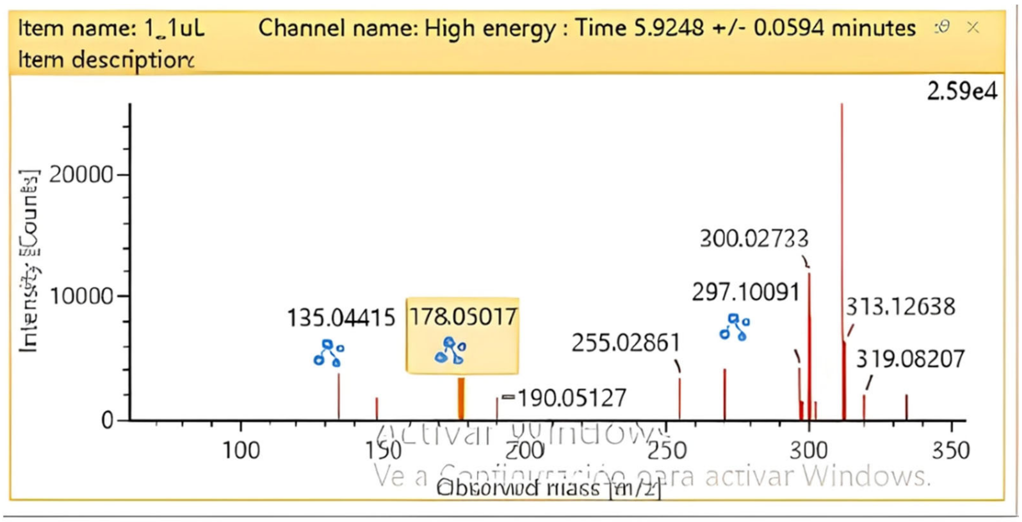
**[M–H]⁻**<br>**(fragment 2 reported)**<br><br>177.0552<br>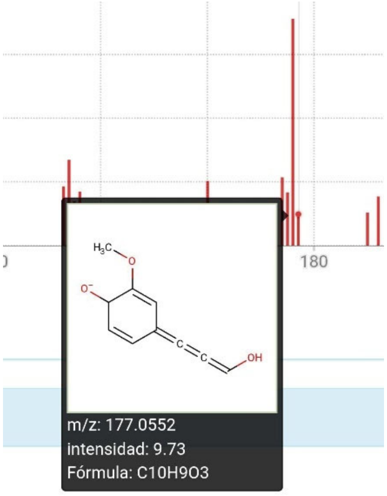<br><br>https://hmdb.ca/spectra/ms_ms/147542
**[M–H]⁻**<br>**(fragment 3 observed)**<br><br>297.1091

Table S1. Fragmentation Pattern Analysis of Proposed Compounds by UPLC-QTOF-MS

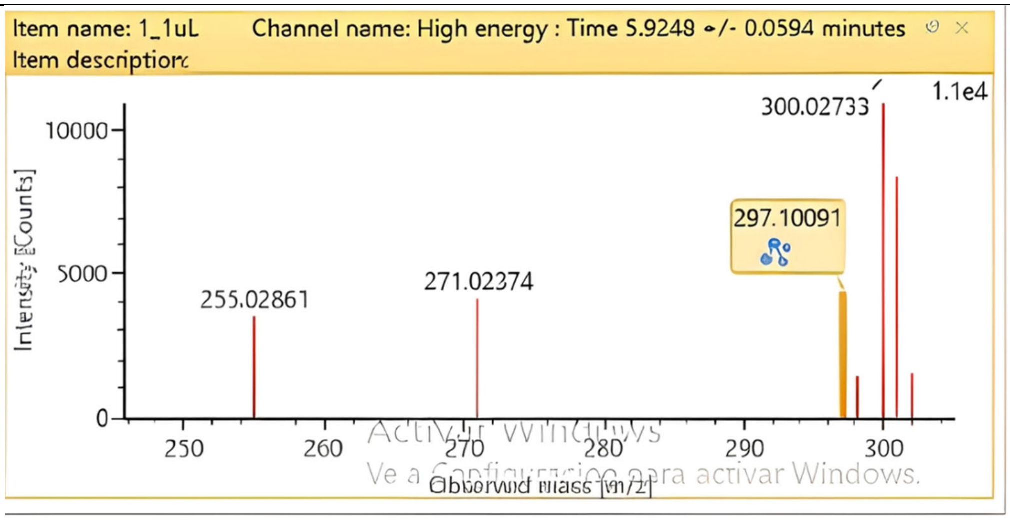
**[M–H]⁻**<br>**(fragment 3 reported)**<br><br>296.0923<br>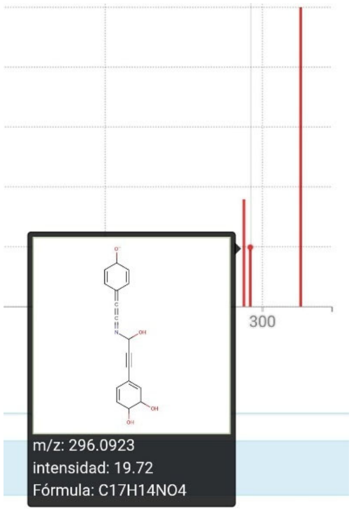<br>https://hmdb.ca/spectra/ms_ms/147541
**Proposed Compound:** Apigenin 7-O-glucoside
**[M–H]⁻**<br><br>**(parent ion observed)**<br><br>431.0987

Table S1. Fragmentation Pattern Analysis of Proposed Compounds by UPLC-QTOF-MS

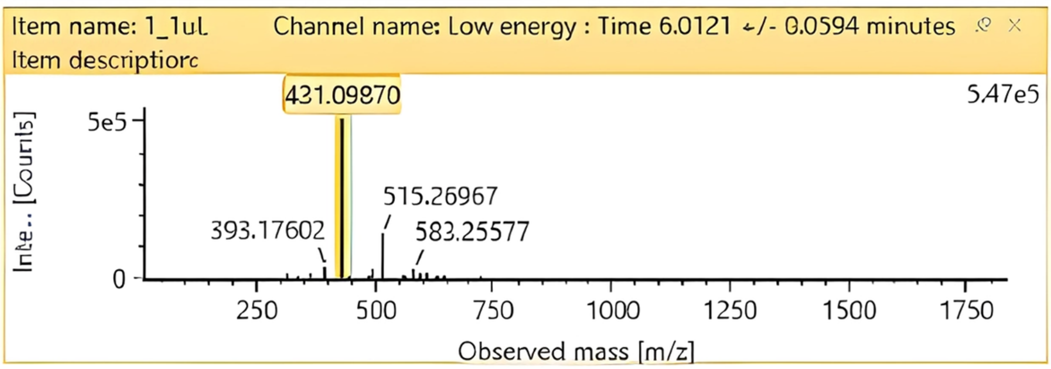
**[M–H]⁻**<br>**(parent ion reported)**<br><br>431.0984<br>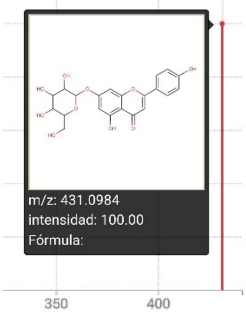<br>https://hmdb.ca/spectra/ms_ms/3606495
**[M–H]⁻**<br>**(fragment 1 observed)**<br><br>**311.05594**<br>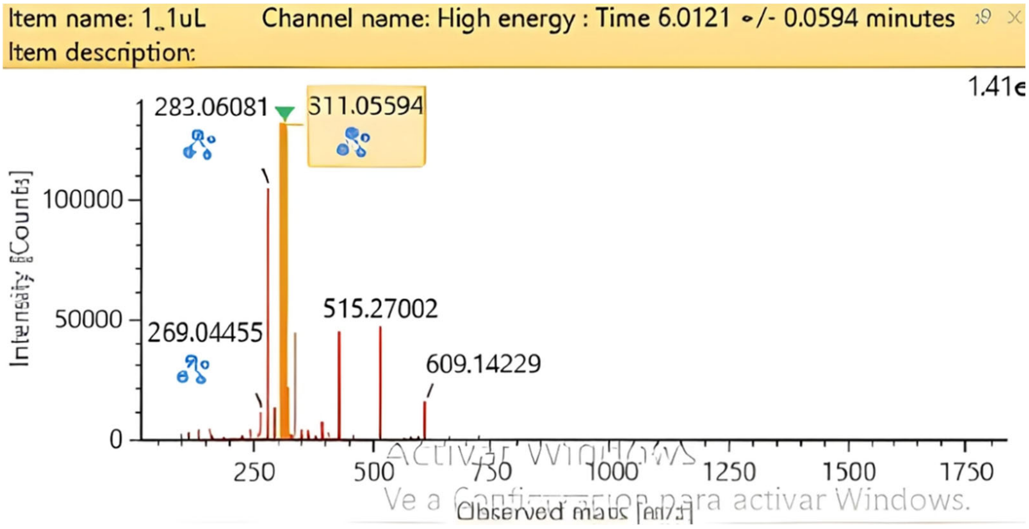

Table S1. Fragmentation Pattern Analysis of Proposed Compounds by UPLC-QTOF-MS

**[M–H]⁻**<br>**(fragment 1 reported)**<br><br>**311.0556**<br>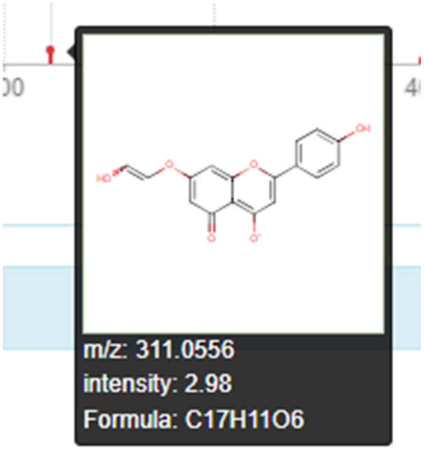<br>https://hmdb.ca/spectra/ms_ms/14101
**[M–H]⁻**<br>**(fragment 2 observed)**<br><br>283.06081<br>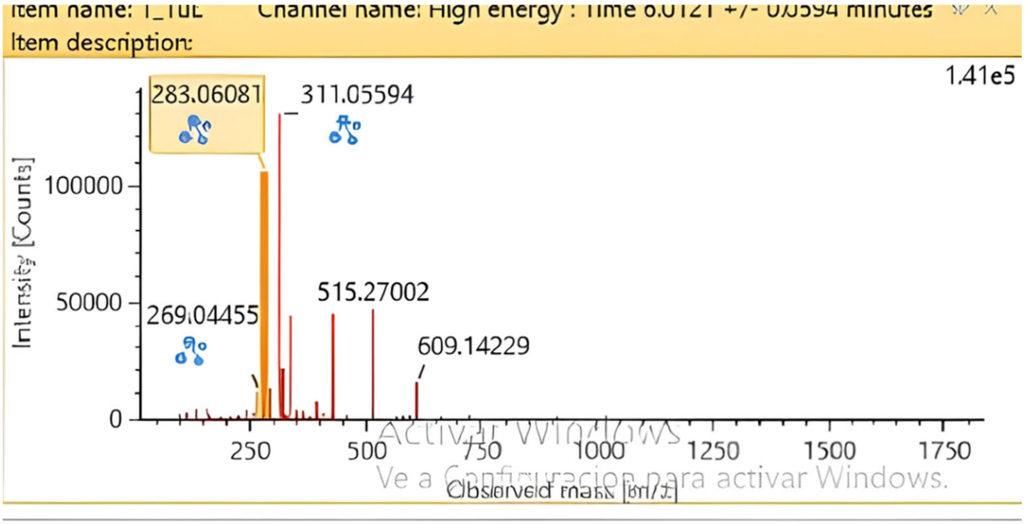
**[M–H]⁻**<br>**(fragment 2 reported)**<br><br>283.0606

Table S1. Fragmentation Pattern Analysis of Proposed Compounds by UPLC-QTOF-MS

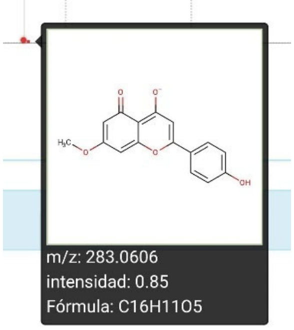

https://hmdb.ca/spectra/ms_ms/15978

[M–H]⁻
(fragment 3 observed)

269.04455

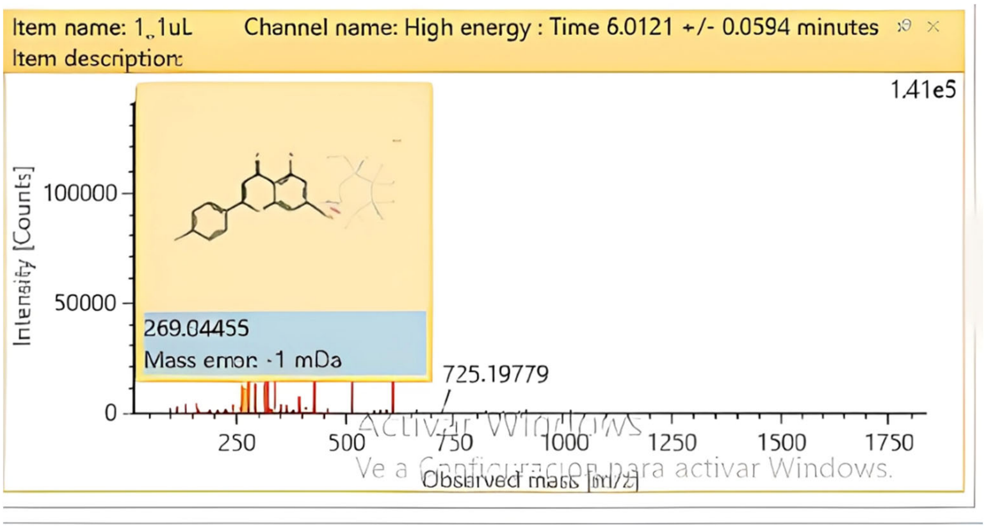

[M–H]⁻
(fragment 3 reported)

269.045

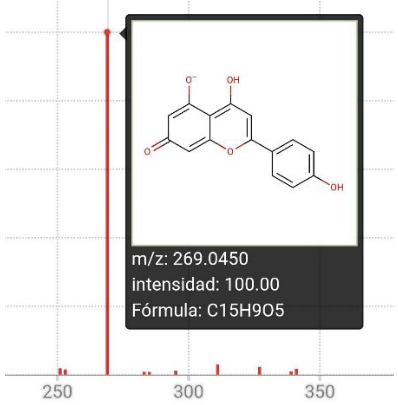

https://hmdb.ca/spectra/ms_ms/15978

Table S1. Fragmentation Pattern Analysis of Proposed Compounds by UPLC-QTOF-MS

Proposed Compound: Apigenin-7-glucuronide

[M–H]⁻

(parent ion observed)

445.0773

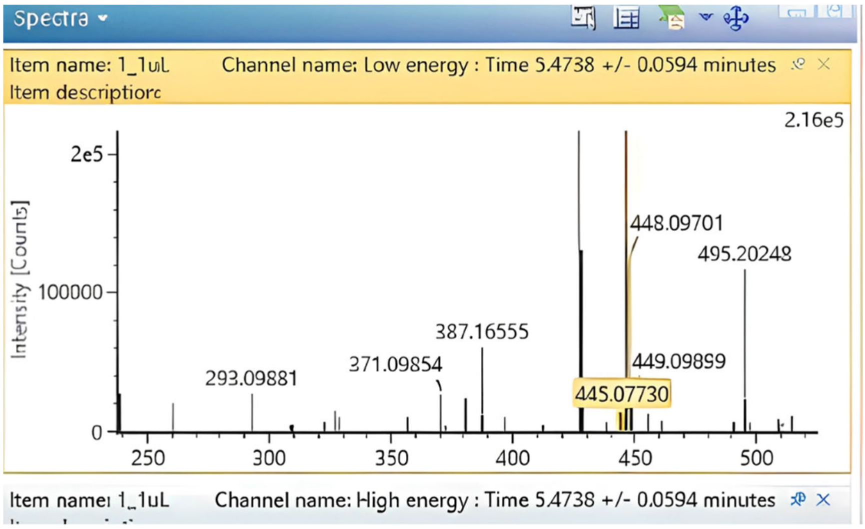

[M–H]⁻
(parent ion reported)

445.0771

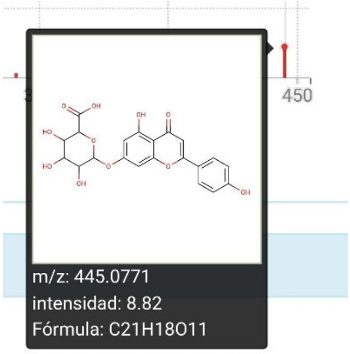

https://hmdb.ca/spectra/ms_ms/116674

[M–H]⁻
(fragment 1 observed)

427.21744

Table S1. Fragmentation Pattern Analysis of Proposed Compounds by UPLC-QTOF-MS

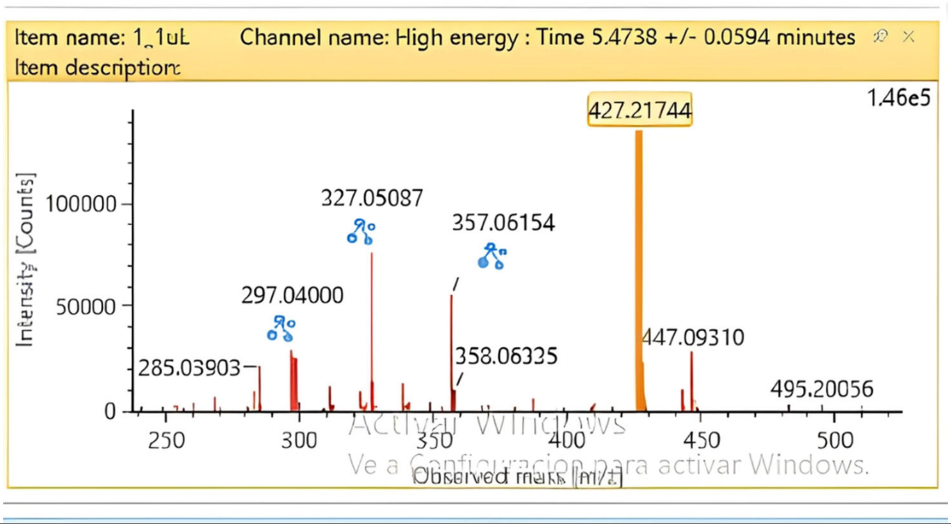
**[M–H]⁻**<br>**(fragment 1 reported)**<br><br>427.0665<br><br>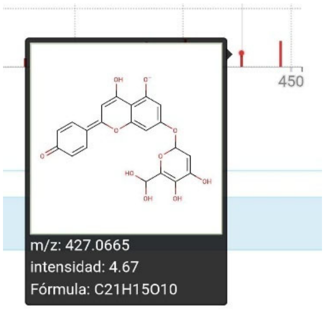<br><br>https://hmdb.ca/spectra/ms_ms/116674
**[M–H]⁻**<br>**(fragment 2 observed)**<br><br>327.05087

Table S1. Fragmentation Pattern Analysis of Proposed Compounds by UPLC-QTOF-MS

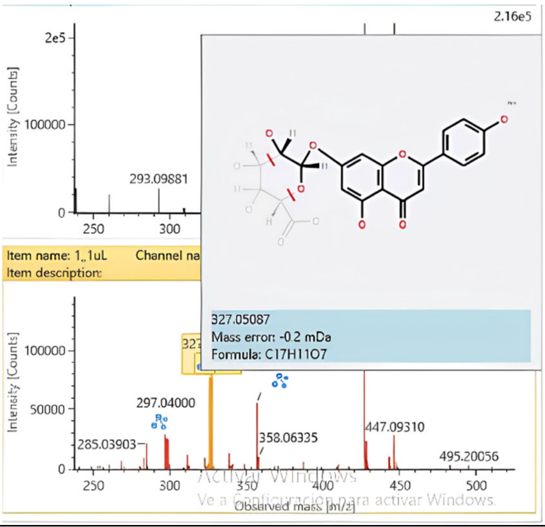

[M–H]⁻
(fragment 2 reported)

327.0505

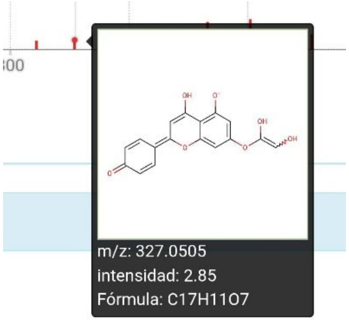

https://hmdb.ca/spectra/ms_ms/116674

[M–H]⁻
(fragment 3 observed)

269.04547

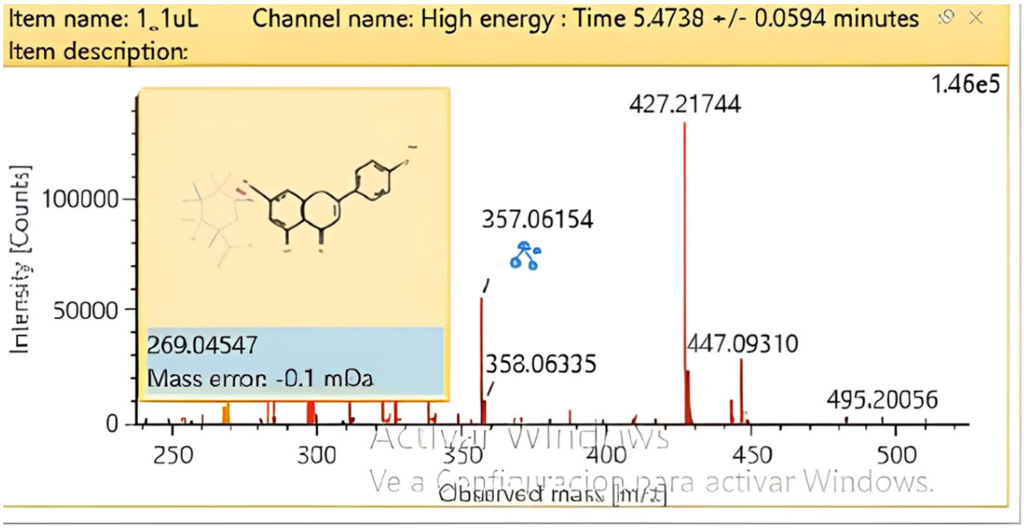

Table S1. Fragmentation Pattern Analysis of Proposed Compounds by UPLC-QTOF-MS

[M–H]⁻
(fragment 3 reported)

269.045

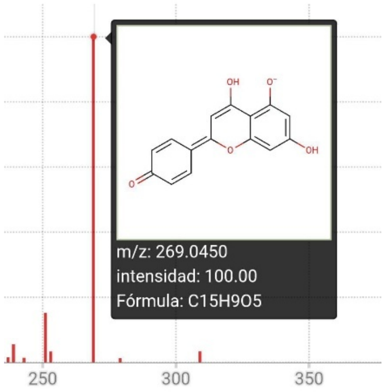

https://hmdb.ca/spectra/ms_ms/116675

Proposed Compound: 3,4-Di-O-caffeoylquinic acid

[M–H]⁻

(parent ion observed)

515.11909

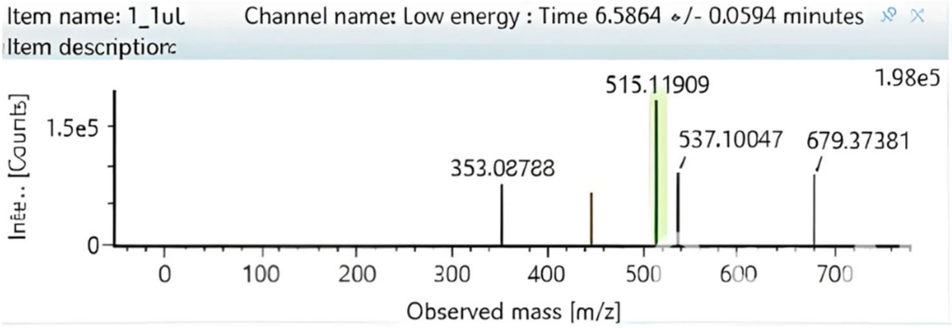

[M–H]⁻
(parent ion reported)

515.1195

Table S1. Fragmentation Pattern Analysis of Proposed Compounds by UPLC-QTOF-MS

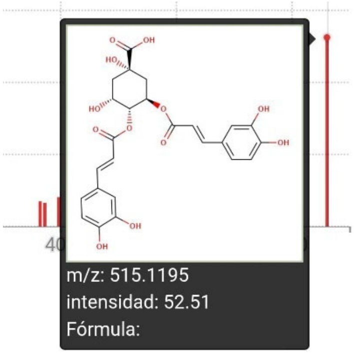<br>https://hmdb.ca/spectra/ms_ms/2839447
**[M–H]⁻**<br>**(fragment 1 observed)**<br>191.05538<br>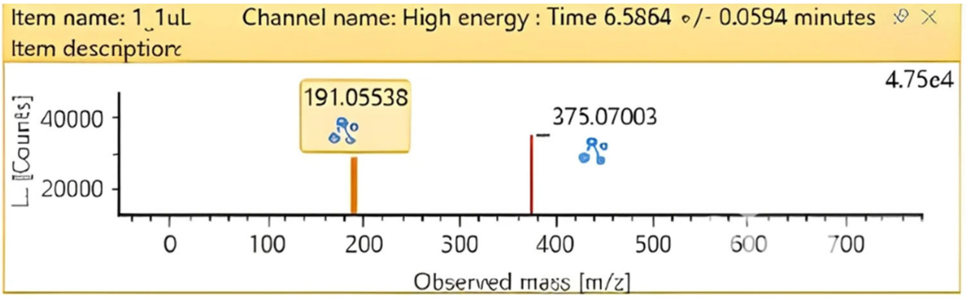
**[M–H]⁻**<br>**(fragment 1 reported)**<br>191.0556<br>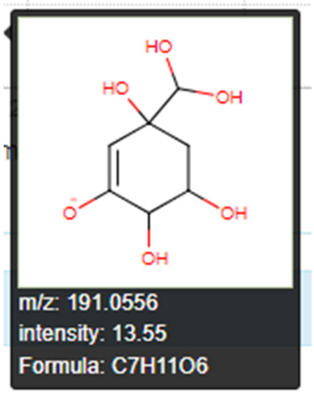<br>https://hmdb.ca/spectra/ms_ms/2839447
**[M–H]⁻**<br>**(fragment 2 observed)**<br>375.07003

Table S1. Fragmentation Pattern Analysis of Proposed Compounds by UPLC-QTOF-MS

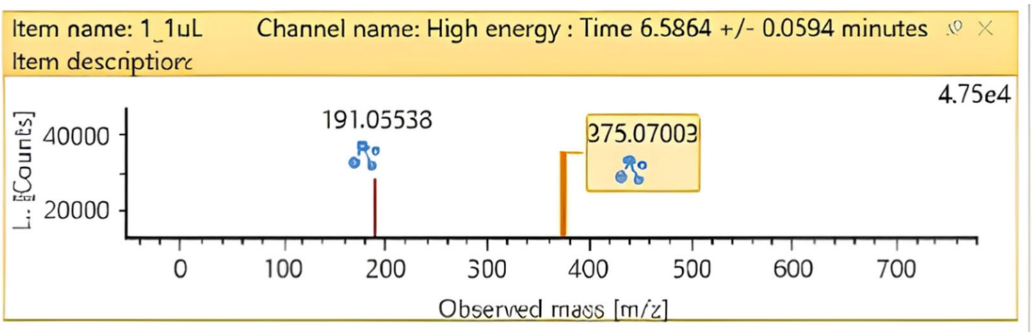
**[M–H]⁻**<br>**(fragment 2 reported)**<br><br>379.0665<br>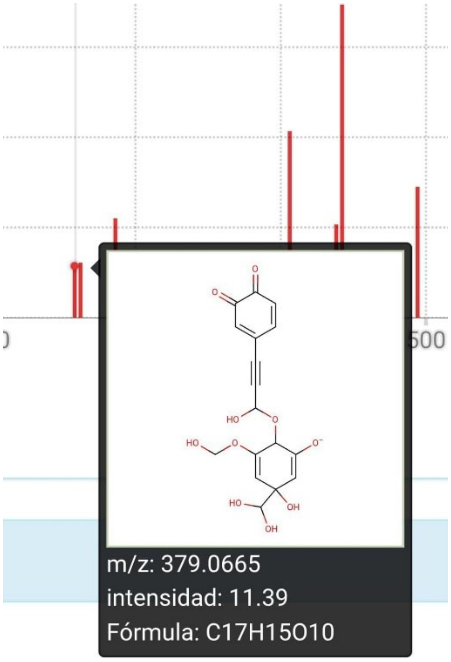<br>https://hmdb.ca/spectra/ms_ms/145900
**[M–H]⁻**<br>**(fragment 3 observed)**<br><br>353.08788

Table S1. Fragmentation Pattern Analysis of Proposed Compounds by UPLC-QTOF-MS

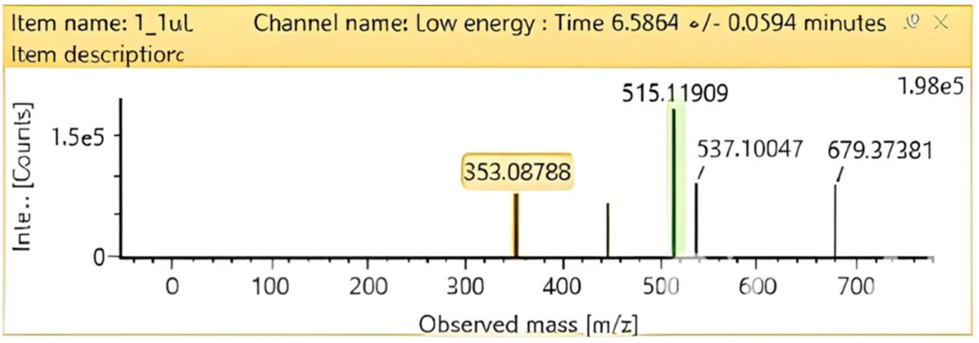
**[M–H]⁻**<br>**(fragment 3 reported)**<br><br>353.0873<br>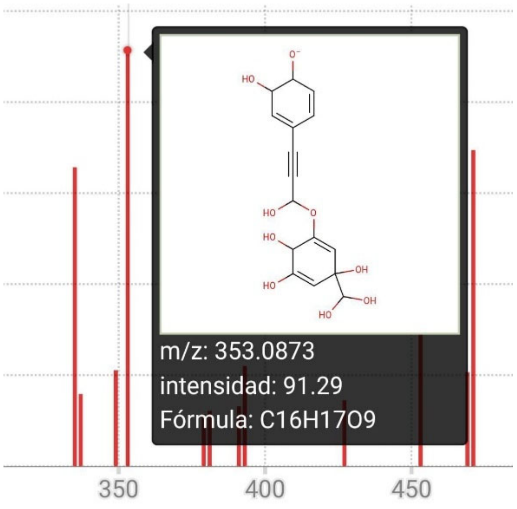<br>https://hmdb.ca/spectra/ms_ms/145900
**Proposed Compound:** 5Z-Caffeoylquinic acid
**[M–H]⁻**<br><br>**(parent ion observed)**<br><br>353.08827<br>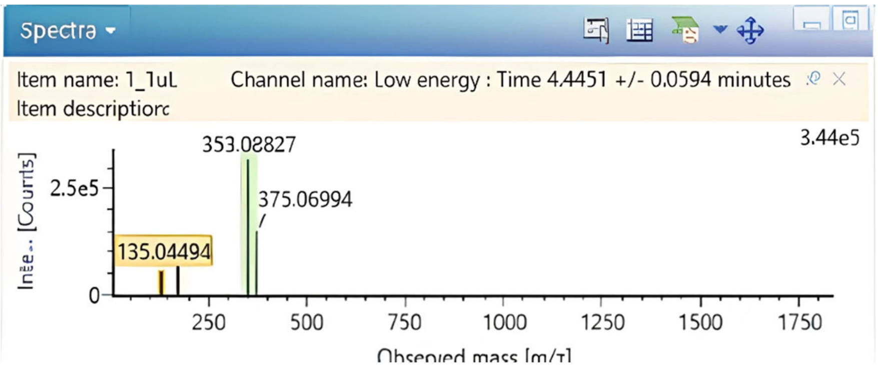

Table S1. Fragmentation Pattern Analysis of Proposed Compounds by UPLC-QTOF-MS

<table>
<tr><td></td></tr>
<tr><td>

[M–H]⁻
(parent ion reported)

353.0878

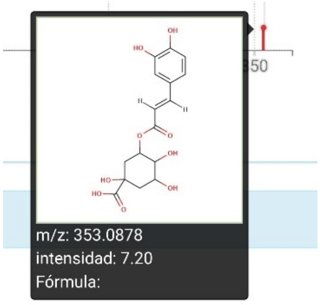

https://hmdb.ca/spectra/ms_ms/2749667

</td></tr>
<tr><td>

[M–H]⁻
(fragment 1 observed)

135.04485

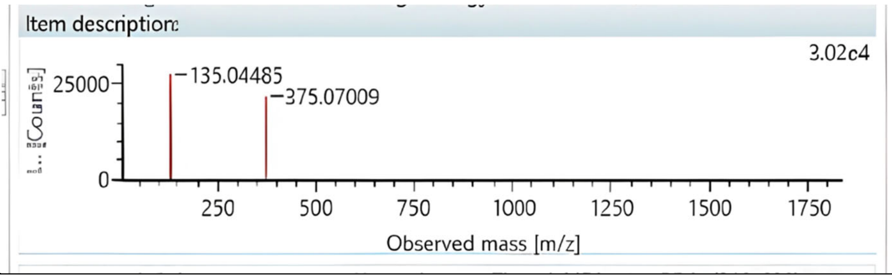

</td></tr>
<tr><td>

[M–H]⁻
(fragment 1 reported)

135.0452

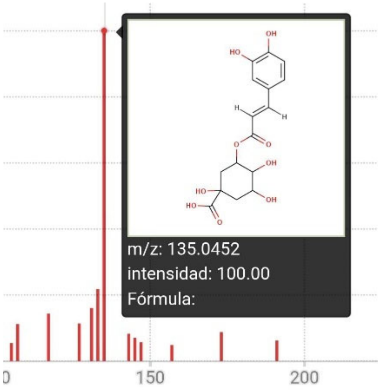

https://hmdb.ca/spectra/ms_ms/2749668

</td></tr>
<tr><td>

[M–H]⁻

</td></tr>
</table>

Table S1. Fragmentation Pattern Analysis of Proposed Compounds by UPLC-QTOF-MS

(fragment 2 observed)

179.03447

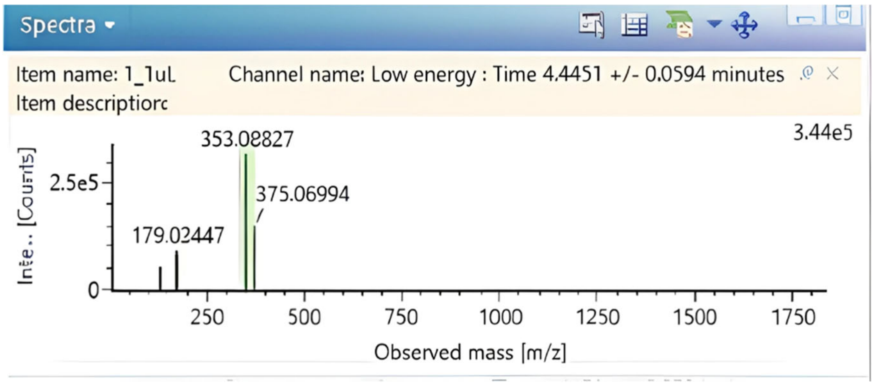

[M–H]⁻

(fragment 2 reported)

179.0344

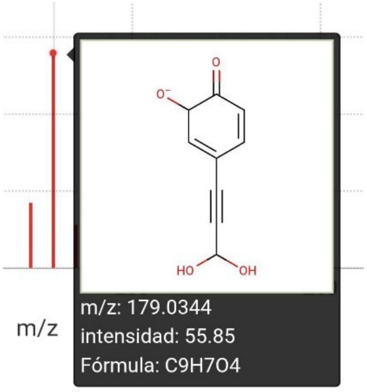

https://hmdb.ca/spectra/ms_ms/138848

Proposed Compound: Diosmetin 7-O-β-D-glucopyranoside

[M–H]⁻

(parent ion observed)

461.10852

Table S1. Fragmentation Pattern Analysis of Proposed Compounds by UPLC-QTOF-MS

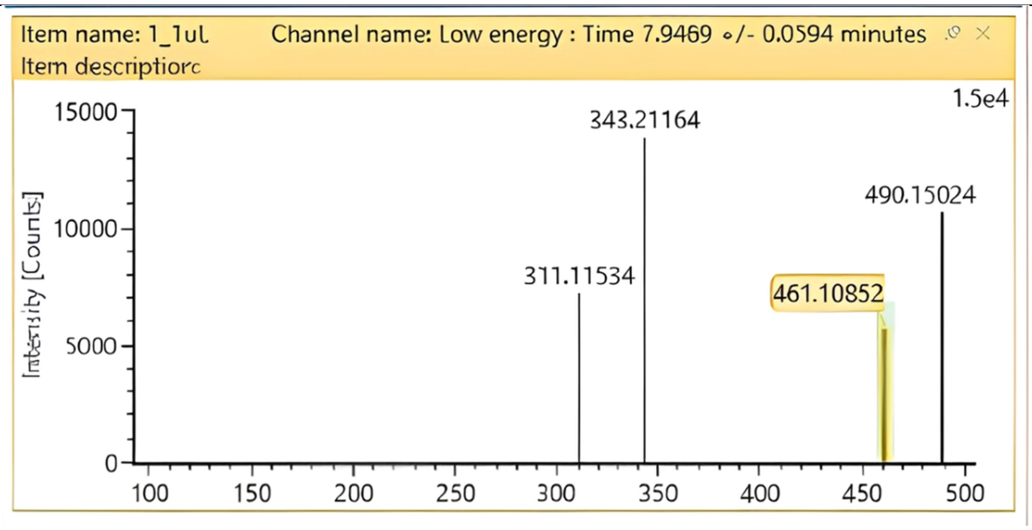
**[M–H]⁻**<br>**(parent ion reported)**<br>461.1089<br>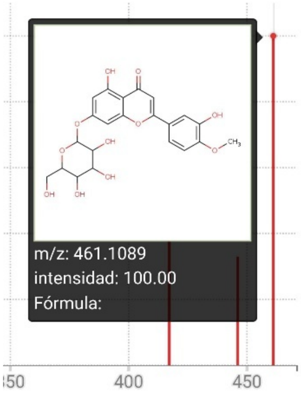 https://hmdb.ca/spectra/ms_ms/3005756
**[M–H]⁻**<br>**(fragment 1 observed)**<br><br>311.05632

Table S1. Fragmentation Pattern Analysis of Proposed Compounds by UPLC-QTOF-MS

<table>
<tr><td>
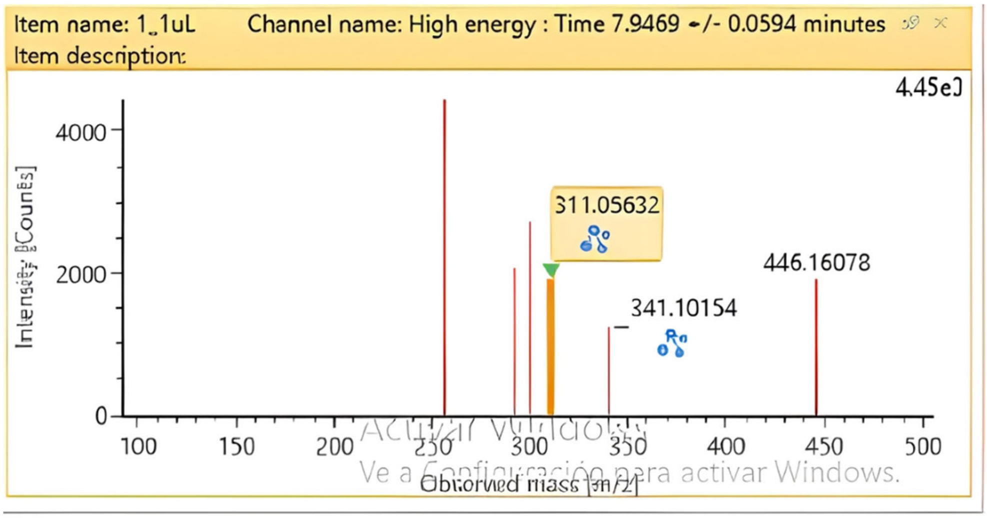

</td></tr>
<tr><td>

[M–H]⁻
(fragment 1 reported)

311.0772

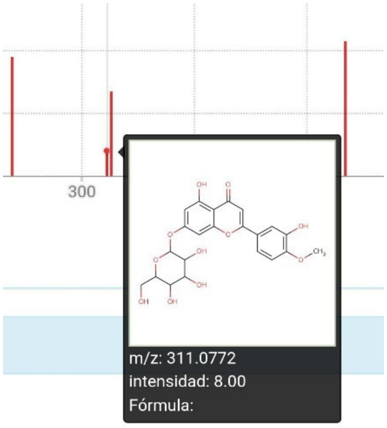

https://hmdb.ca/spectra/ms_ms/3005756
</td></tr>
<tr><td>

[M–H]⁻
(fragment 2 observed)

341.10154
</td></tr>
</table>

Table S1. Fragmentation Pattern Analysis of Proposed Compounds by UPLC-QTOF-MS

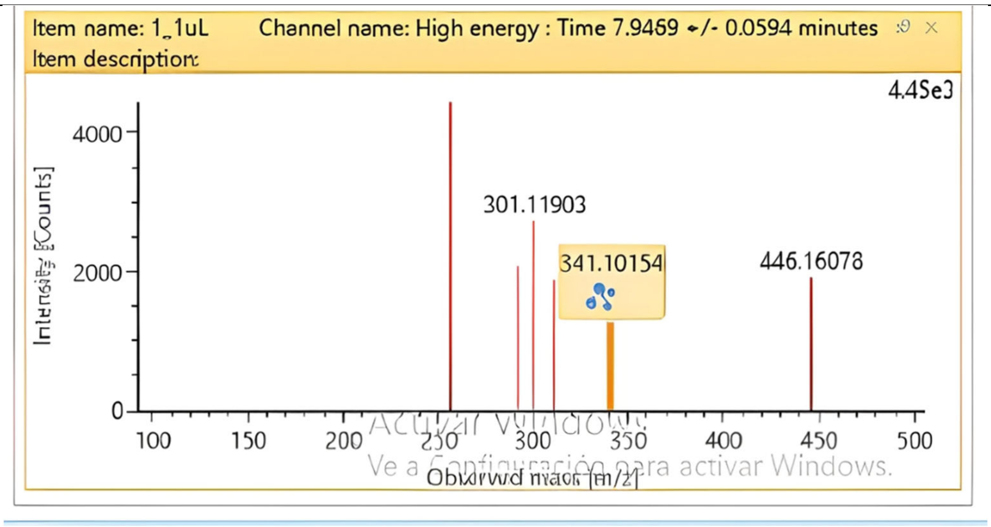
**[M–H]⁻**<br>**(fragment 2 reported)**<br><br>341.0661<br>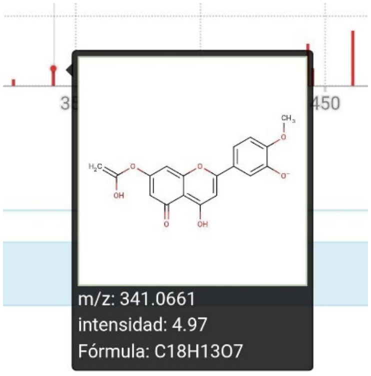<br><br>https://hmdb.ca/spectra/ms_ms/136327
**[M–H]⁻**<br>**(fragment 3 observed)**<br><br>446.16078

Table S1. Fragmentation Pattern Analysis of Proposed Compounds by UPLC-QTOF-MS

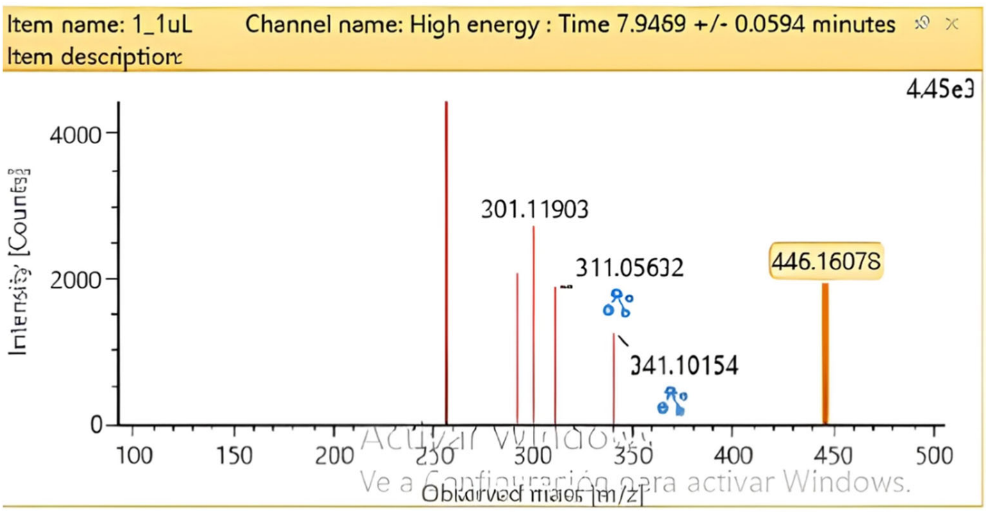

[M–H]⁻

(fragment 3 reported)

446.0854

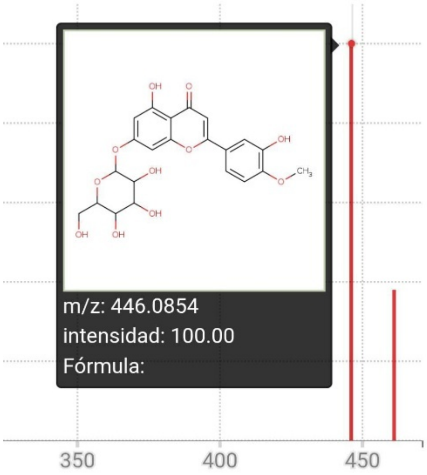

https://hmdb.ca/spectra/ms_ms/3005755

Proposed Compound: (-)-Arctigenin

[M–H]⁻

(parent ion observed)

371.14895

Table S1. Fragmentation Pattern Analysis of Proposed Compounds by UPLC-QTOF-MS

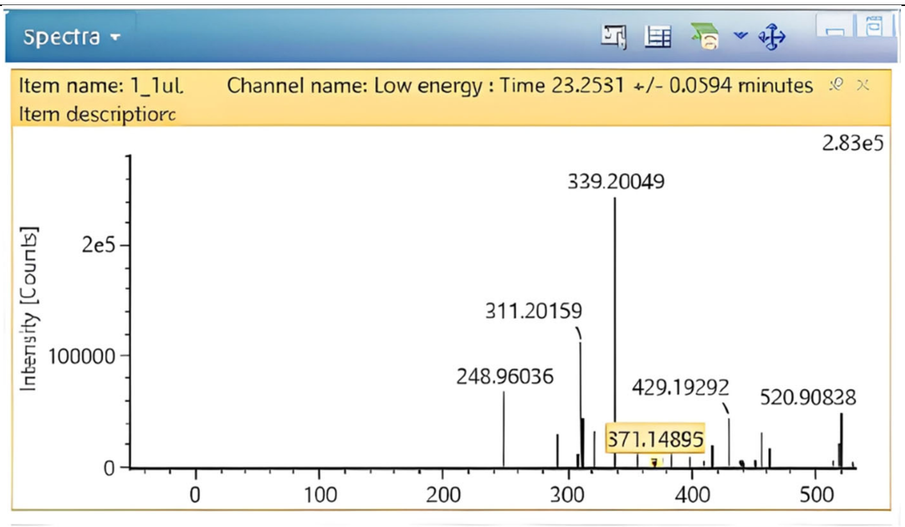
**[M–H]⁻**<br>**(parent ion reported)**<br><br>371.1495<br>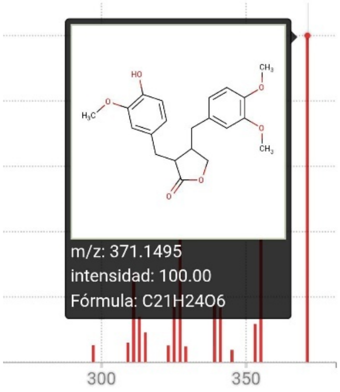<br>https://hmdb.ca/spectra/ms_ms/123871
**[M–H]⁻**<br>**(fragment 1 observed)**<br><br>269.24585

Table S1. Fragmentation Pattern Analysis of Proposed Compounds by UPLC-QTOF-MS

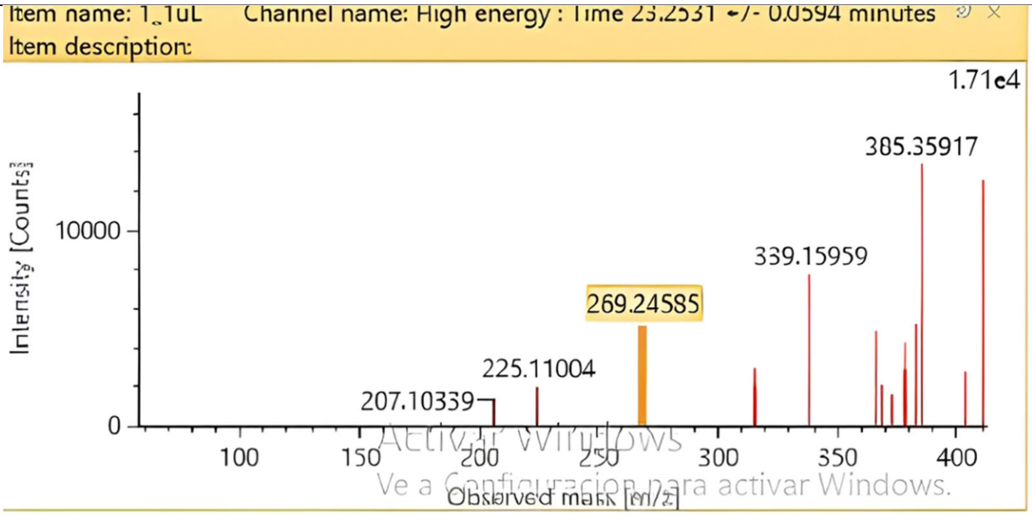
**[M–H]⁻**<br>**(fragment 1 reported)**<br><br>269.1178<br>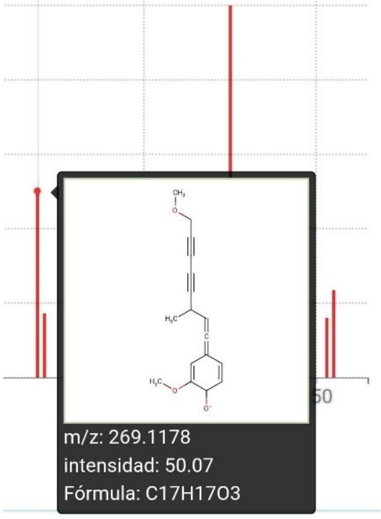<br>https://hmdb.ca/spectra/ms_ms/123872
**[M–H]⁻**<br>**(fragment 2 observed)**<br><br>339.15959

Table S1. Fragmentation Pattern Analysis of Proposed Compounds by UPLC-QTOF-MS

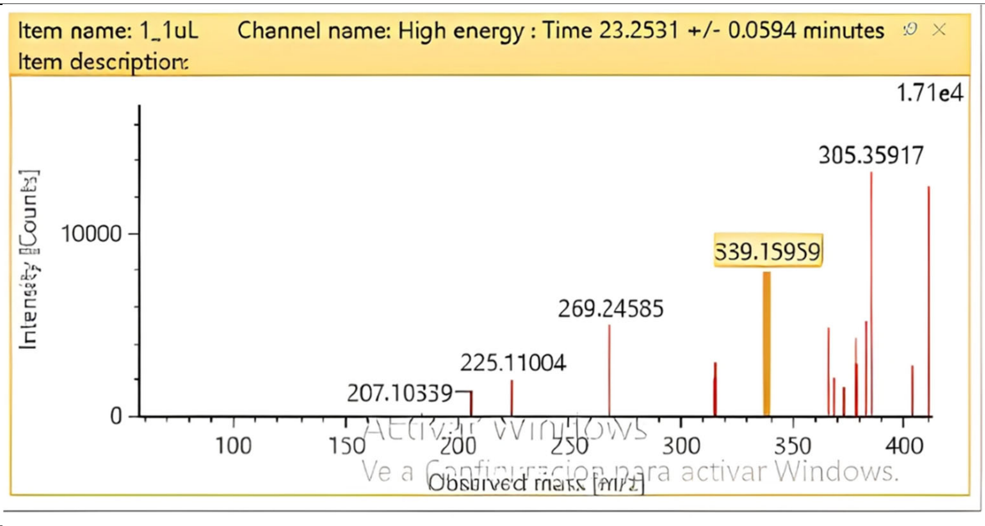

[M–H]⁻

(fragment 2 reported)

339.0869

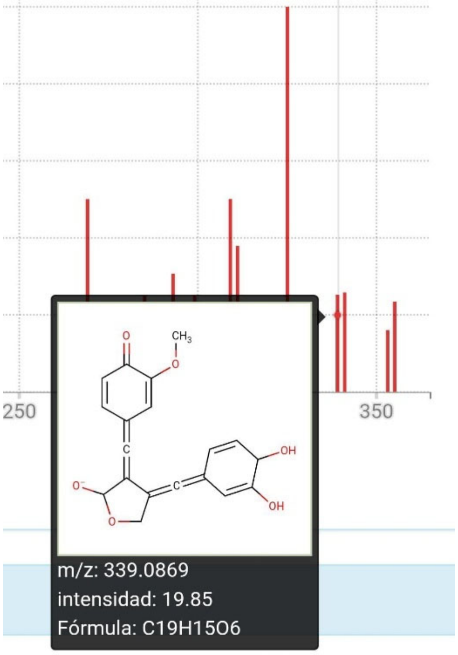

https://hmdb.ca/spectra/ms_ms/123872

Proposed Compound: Betulinic acid

[M–H]⁻

(parent ion observed)

455.35226

Table S1. Fragmentation Pattern Analysis of Proposed Compounds by UPLC-QTOF-MS

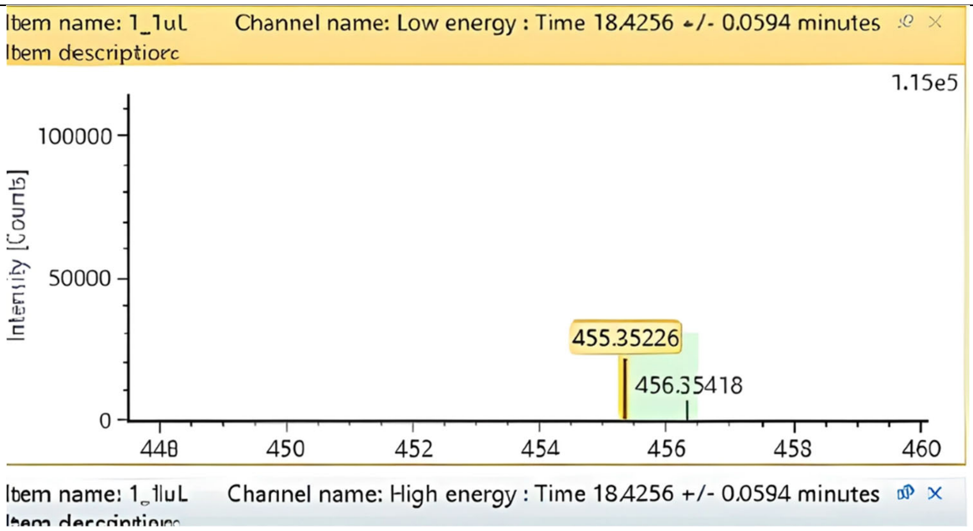
**[M–H]⁻**<br>**(parent ion reported)**<br><br>455.3525<br>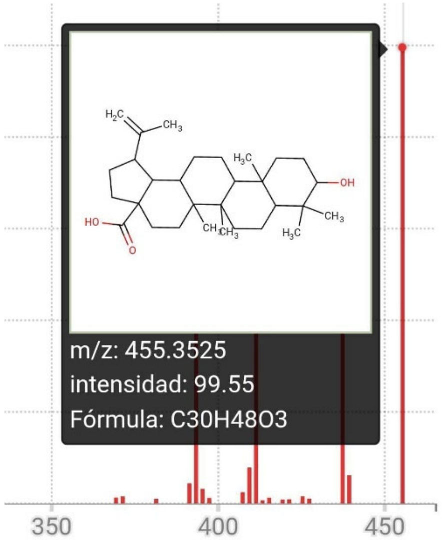<br>https://hmdb.ca/spectra/ms_ms/242951
**[M–H]⁻**<br>**(fragment 1 observed)**<br><br>275.19835

Table S1. Fragmentation Pattern Analysis of Proposed Compounds by UPLC-QTOF-MS

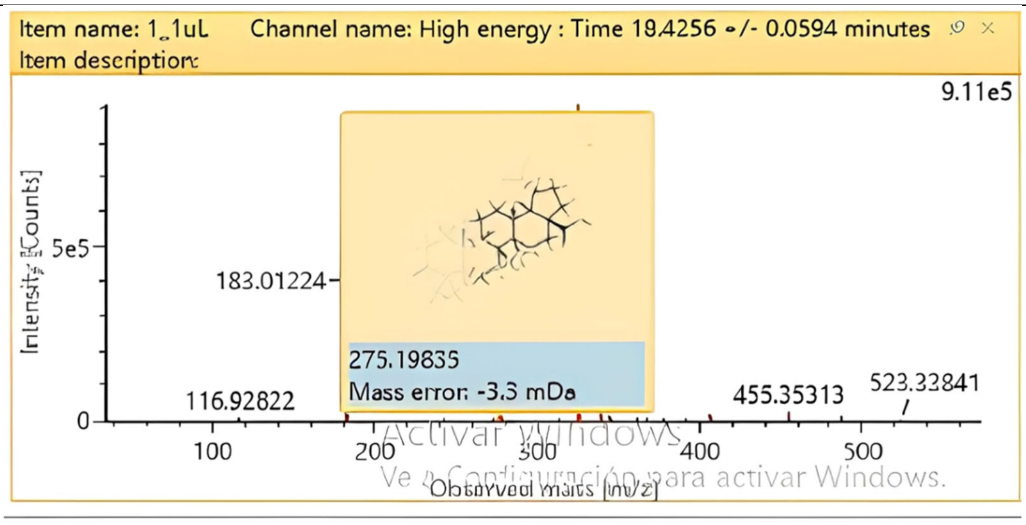

[M–H]⁻
(fragment 1 reported)

275.2575

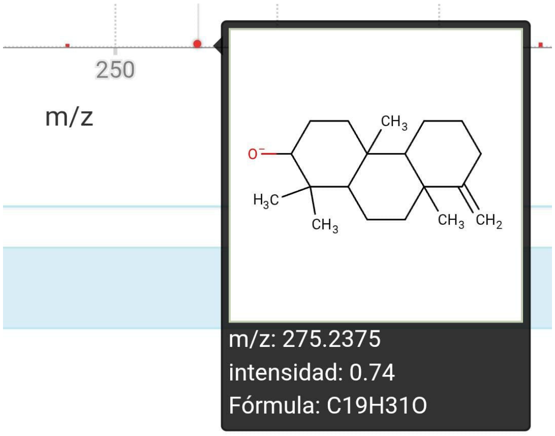

https://hmdb.ca/spectra/ms_ms/242951

[M–H]⁻
(fragment 2 observed)

407.17605

Table S1. Fragmentation Pattern Analysis of Proposed Compounds by UPLC-QTOF-MS

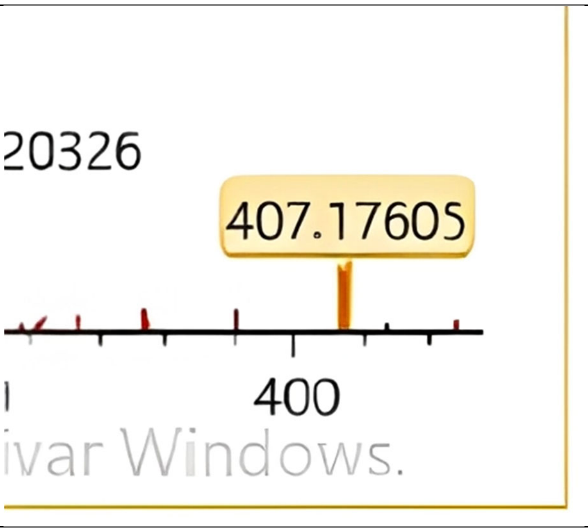
**$[M–H]^-$**<br>**(fragment 2 reported)**<br><br>407.3314<br>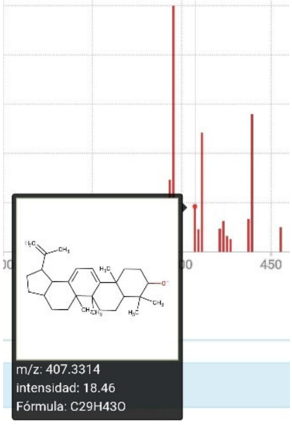<br>https://hmdb.ca/spectra/ms_ms/242952
**$[M–H]^-$**<br>**(fragment 3 observed)**<br><br>425.169<br>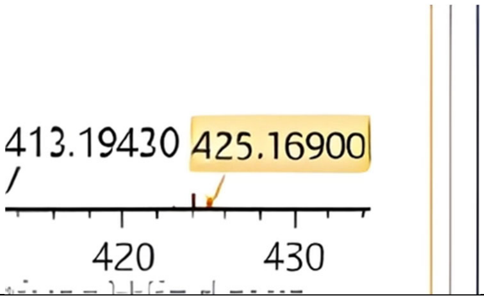
**$[M–H]^-$**<br>**(fragment 3 reported)**<br><br>425.3056

Table S1. Fragmentation Pattern Analysis of Proposed Compounds by UPLC-QTOF-MS

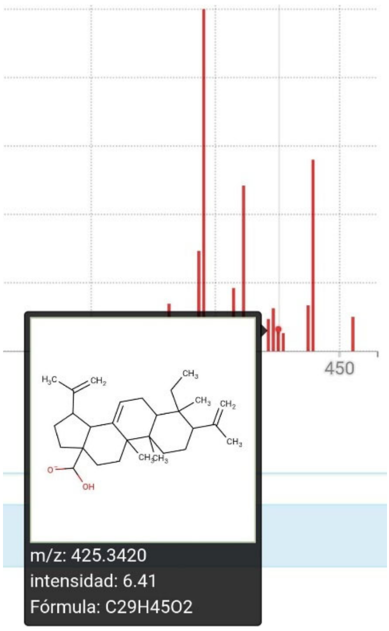

https://hmdb.ca/spectra/ms_ms/242952
